# Ultrasonic-assisted unusual four-component synthesis of 7-azolylamino-4,5,6,7-tetrahydroazolo[1,5-*a*]pyrimidines

**DOI:** 10.3762/bjoc.16.27

**Published:** 2020-02-27

**Authors:** Yana I Sakhno, Maryna V Murlykina, Oleksandr I Zbruyev, Anton V Kozyryev, Svetlana V Shishkina, Dmytro Sysoiev, Vladimir I Musatov, Sergey M Desenko, Valentyn A Chebanov

**Affiliations:** 1 Division of Chemistry of Functional Materials, State Scientific Institution “Institute for Single Crystals” of National Academy of Sciences of Ukraine, Nauky Av. 60, 61072 Kharkiv, Ukraine; 2 Faculty of Chemistry, V. N. Karazin Kharkiv National University, Svobody Sq. 4, 61077 Kharkiv, Ukraine; 3 Institute of Organic Chemistry and Biochemistry of the Czech Academy of Sciences, Flemingovo náměstí 542/2, 166 10 Praha 6, Czech Republic

**Keywords:** 5-amino-1*H*-pyrazole-4-carbonitrile, 3-amino-1,2,4-triazole, 7-azolylaminotetrahydroazolo[1,5-*a*]pyrimidines, heterocycle, multicomponent reaction, ultrasonication

## Abstract

Four-component reactions of 3-amino-1,2,4-triazole or 5-amino-1*H*-pyrazole-4-carbonitrile with aromatic aldehydes and pyruvic acid or its esters under ultrasonication were studied. Unusual for such a reaction type, a cascade of elementary stages led to the formation of 7-azolylaminotetrahydroazolo[1,5-*a*]pyrimidines.

## Introduction

**Scheme 1:**
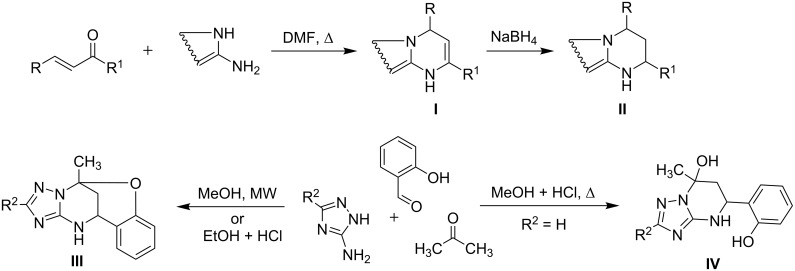
Synthesis of tetrahydroazolopyrimidine derivatives.

**Scheme 2:**
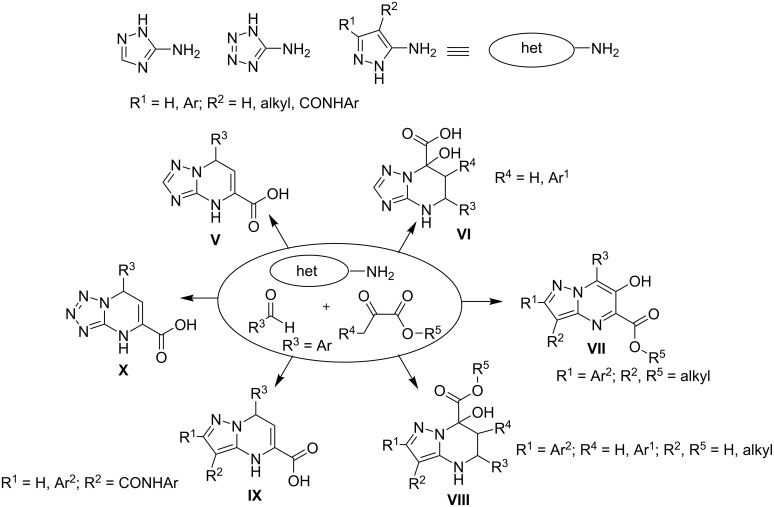
Various multicomponent reactions involving pyruvic acids (pyruvates) and different α-aminoazoles.

Tetrahydropyrimidines are heterocycles of high pharmacological importance, and they have attracted the attention of medicinal chemists because of their various biological activities [1]. Furthermore, tetrahydropyrimidines containing fused azole rings are a privileged class of heterocycles due to their antiviral [2], antitubercular, antitumor [3], antibacterial [4–5], and bone-anabolic activities [6–7]. Previously, some tetrahydroazolopyrimidines **II** (Scheme 1) were synthesized using sequential synthetic routes [8–15], in most cases involving the reduction of the dihydroazolopyrimidines **I** obtained via well-known reactions of aminoazoles with α,β-unsaturated ketones [8–11] or via their multicomponent analogues. On the other hand, multicomponent reactions (MCRs) directly leading to tetrahydroazolopyrimidine heterocyclic systems have also been published [4–5,16–24]. In particular, multicomponent approaches were described for the synthesis of 7-hydroxytetrahydroazolopyrimidines by the reaction of aminoazoles, aromatic aldehydes, and some carbonyl compounds (Scheme 1, **IV** and Scheme 2, **VI** and **VIII**) [4–5,16–23]. Oxygen-bridged tetrahydroazolopyrimidines **III** were also obtained via similar MCRs when using salicylic aldehydes, allowing further intermolecular cyclization [23–24].

One of the promising reagents for the synthesis of diverse types of azoloazines by multicomponent and sequential reactions is pyruvic acid, along with its derivatives (Scheme 2). The selectivity of such interactions can be tuned, for example, by application of a condition-based divergence strategy [25], which is based on the variation of solvents, catalysts, and activation methods [16,26–29].

Thus, using nonclassical activation methods such as ultrasonication and microwave irradiation enabled us to develop highly selective procedures for obtaining triazolo-, tetrazolo-, and pyrazolopyrimidines [4–5,16–20,27–29], as well as oxygen-bridged tetrahydropyrazolopyrimidines [4,19], furanone [17,30], and pyrrolone derivatives [5,16–18].

**Scheme 3:**
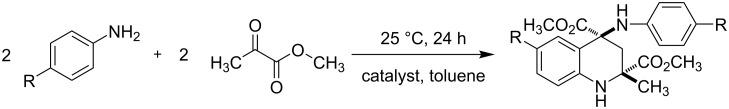
Synthesis of 4-arylamino-substituted tetrahydroquinolines.

Besides the presence of an OH group in the tetrahydropyrimidine moiety (e.g., Scheme 2, **VI** and **VIII**), the formation of heterocycles that are amino-substituted in a similar position is also possible and has been described [31–32]. In particular, the reaction of methyl pyruvate and anilines led to the formation of 4-arylamino-substituted tetrahydroquinolines (Scheme 3). To the best of our knowledge, an analogous reaction has not been described for aminoazoles.

We were interested to study reactions similar to the ones shown in Scheme 3, involving aromatic aldehydes and pyruvic acid but using aminoazoles instead of anilines. Therefore, the present work is devoted to the study of multicomponent reactions involving 3-amino-1,2,4-triazole or 5-aminopyrazoles, aromatic aldehydes, and pyruvic acid or its esters under ultrasonication.

## Results and Discussion

**Scheme 4:**
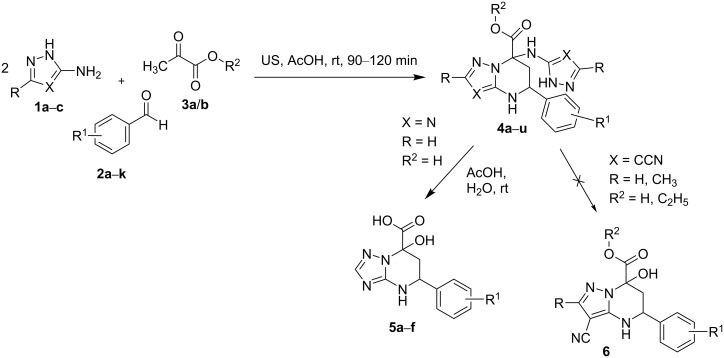
Ultrasound-assisted multicomponent reactions of 3-amino-1,2,4-triazole or 5-amino-1*H*-pyrazole-4-carbonitrile, aldehydes, and pyruvic acid/ethyl pyruvate.

It was discovered that MCRs involving 3-amino-1,2,4-triazole or 5-amino-1*H*-pyrazole-4-carbonitrile with aromatic aldehydes and pyruvic acid or its esters under ultrasonication led to the formation of 4,5,6,7-tetrahydroazolo[1,5-*a*]pyrimidines **4a**–**u** (Scheme 4) containing an azolylamino substituent in the 7-position via an unusual pseudo four-component reaction, rather than two heterocycles of the types **V**–**X** (Scheme 2). It should be noted that such a type of MCR, giving previously undescribed 7-azolylamino-substituted tetrahydroazolopyrimidines, is reported for the first time herein.

Thus, using 2 equivalents of 5-aminopyrazole-4-carbonitrile **1a**/**b** in MCRs with aromatic aldehydes **2a**–**c** and pyruvic acid (**3a**) in acetic acid at room temperature under ultrasonication for 90 min gave 3-cyano-7-((4-cyano-1*H*-pyrazol-5-yl)amino)-5-aryl-4,5,6,7-tetrahydropyrazolo[1,5-*a*]pyrimidine-7-carboxylic acids **4a**–**c**. There is also the possibility of applying ethyl pyruvate (**3b**) instead of pyruvic acid as a reactant in the reaction with 3-substituted-5-aminopyrazole-4-carbonitriles (R = H, CH_3_) **1a**/**b** and aromatic aldehydes **2a**–**f** (ultrasonication in acetic acid at room temperature for 120 min). In this case, the corresponding ethyl 3-cyano-7-((4-cyano-3-substituted-1*H*-pyrazol-5-yl)amino)-2-substituted-5-aryl-4,5,6,7-tetrahydropyrazolo[1,5-*a*]pyrimidine-7-carboxylates (substituents = H, CH_3_) **4d**–**o** were isolated in 45–80% yields (Scheme 4, Table 1).

**Table 1:** Synthesis of compounds **4a**–**u**.

starting material							US reaction time (min)	product	yield (%)
**1**	X	R	R^1^	**2**	R^2^	**3**		**4**	

**1a**	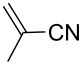	H	H	**2a**	H	**3a**	90	**4a**	76
**1a**	„	H	4-CH_3_O	**2b**	H	**3a**	90	**4b**	86
**1a**	„	H	4-Cl	**2c**	H	**3a**	90	**4c**	75
**1a**	„	H	4-H	**2a**	C_2_H_5_	**3b**	120	**4d**	60
**1a**	„	H	4-CH_3_O	**2b**	C_2_H_5_	**3b**	120	**4e**	73
**1a**	„	H	4-Cl	**2c**	C_2_H_5_	**3b**	120	**4f**	55
**1a**	„	H	4-Br	**2d**	C_2_H_5_	**3b**	120	**4g**	80
**1a**	„	H	4-COOCH_3_	**2e**	C_2_H_5_	**3b**	120	**4h**	64
**1a**	„	H	4-CN	**2f**	C_2_H_5_	**3b**	120	**4i**	53
**1b**	„	CH_3_	H	**2a**	C_2_H_5_	**3b**	120	**4j**	45
**1b**	„	CH_3_	4-CH_3_O	**2b**	C_2_H_5_	**3b**	120	**4k**	68
**1b**	„	CH_3_	4-Cl	**2c**	C_2_H_5_	**3b**	120	**4l**	55
**1b**	„	CH_3_	4-Br	**2d**	C_2_H_5_	**3b**	120	**4m**	63
**1b**	„	CH_3_	4-COOCH_3_	**2e**	C_2_H_5_	**3b**	120	**4n**	55
**1b**	„	CH_3_	4-CN	**2f**	C_2_H_5_	**3b**	120	**4o**	47
**1c**	N	H	4-CH_3_O	**2b**	H	**3a**	120	**4p**	60
**1c**	N	H	3-CH_3_O	**2g**	H	**3a**	120	**4q**	65
**1c**	N	H	2-CH_3_O	**2h**	H	**3a**	120	**4r**	70
**1c**	N	H	4-OH	**2k**	H	**3a**	120	**4s**	76
**1c**	N	H	3-OH	**2j**	H	**3a**	120	**4t**	34
**1c**	N	H	2-OH	**2i**	H	**3a**	120	**4u**	49

The same products were isolated while carrying out this reaction in acetic acid at room temperature with intensive stirring instead of ultrasonic irradiation. However, the reaction time had to be increased to 24 h, and the yields and purity of the compounds **4** decreased (as seen via TLC and NMR analysis), obviously due to the worse homogenization and mass transfer compared to ultrasonication.

Literature data [17,26–27,33–35] indicates that 5-aminopyrazoles bearing an electron-withdrawing substituent in the 4-position, such as a carbonitrile group, often behave similar to 3-amino-1,2,4-triazole; therefore, we studied the latter under the same reaction conditions. We showed that the pseudo four-component heterocyclization of two equivalents of 3-amino-1,2,4-triazole (**1c**) with aromatic aldehydes **2a**–**f** and pyruvic acid (**3a**) carried out in acetic acid at a room temperature under ultrasonication for 120 min gave 7-((1*H*-1,2,4-triazol-5-yl)amino)-5-aryl-4,5,6,7-tetrahydro[1,2,4]triazolo[1,5-*a*]pyrimidine-7-carboxylic acids **4p**–**u** in 34–76% yields (Scheme 4). In contrast to pyrazolyl-substituted pyrazolo[1,5-*a*]pyrimidine-7-carboxylic acids **4d**–**o**, triazolyl-substituted derivatives **4p**–**u** were unstable in protic solvents in the presence of water, and especially upon increasing the temperature gradually transformed into 7-hydroxytriazolo[1,5-*a*]pyrimidines **5a**–**f**, which had been obtained earlier [5] by the three-component reaction of starting materials **1c**, **2a**–**f**, and **3a** (HOAc, 65 °C, 48 h). This instability complicated the isolation and characterization of the heterocycles **4p**–**u**. The condensation of compounds **1c**, **2a**–**f**, and **3a** in dry DMF under otherwise identical conditions was a way to avoid the rapid conversion of triazolyl derivatives **4p**–**u** into 7-hydroxytriazolo[1,5-*a*]pyrimidines **5a**–**f** and to isolate the heterocycles **4p**–**u**, but in lower yields in comparison to the reaction in acetic acid.

We consequently screened various condensation conditions, specifically by applying different temperatures in the range of 0–110 °C (both with the help of conventional heating and microwave irradiation) and by using different solvents and catalysts, such as HOAc, DMSO, primary alcohols with and without the presence of HCl, Yb(OTf)_3_, or Et_3_N. In all these cases, mixtures of triazolo[1,5-*a*]pyrimidines **4p**–**u** and **5a**–**f** in different ratios (the content of compounds **5a**–**f** increased with the elevation of temperature and time) with impurities of starting reagents and unidentified compounds were obtained.

**Scheme 5:**
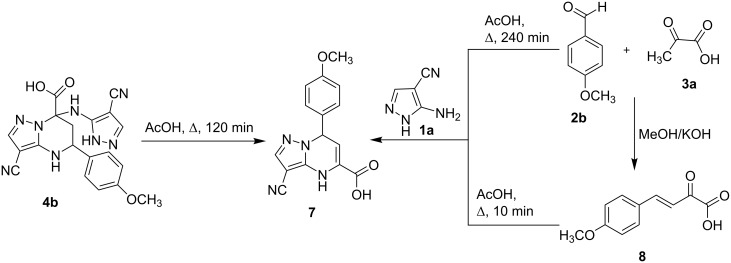
Synthesis of 3-cyano-7-(4-methoxyphenyl)-4,7-dihydropyrazolo[1,5-*a*]pyrimidine-5-carboxylic acid (**7**).

In contrast to tetrahydropyrimidines **4p**–**u**, containing triazolyl fragments, our attempts to obtain compounds **6** from pyrazolyl derivatives **4b**/**c**/**e**/**f** were unsuccessful. In particular, under the same conditions (prolonged stirring in acetic acid at temperatures up to 70 °C), the starting tetrahydropyrazolopyrimidines **4b**/**c**/**e**/**f** remained unchanged. Then, it was established that compound **4e** remained stable under refluxing in acetic acid for 60 min, while heating for 120–180 min led to its decomposition. At the same time, compound **4b**, after refluxing in acetic acid for ca. 120 min, was converted into 3-cyano-7-(4-methoxyphenyl)-4,7-dihydropyrazolo[1,5-*a*]pyrimidine-5-carboxylic acid (**7**, yield 35%). The same carboxylic acid **7** was obtained by counter synthesis through a three-component reaction involving 5-aminopyrazole-4-carbonitrile (**1a**), 4-methoxybenzaldehyde (**2b**), and pyruvic acid (**3a**) under heating at reflux in acetic acid for 240 min (yield 28%) or through two-component heterocyclization of 5-aminopyrazole **1a** and *p*-methoxybenzylidenepyruvic acid (**8**, obtained from *p*-methoxybenzaldehyde and pyruvic acid) under the same conditions in a much shorter reaction time (10 min) with 66% yield (Scheme 5).

The results presented above indicate that the reaction possibly proceeded under kinetic and thermodynamic control, depending on the conditions. In particular, ultrasonication of the starting materials **1a**, **2b**, and **3a** for 90 min at room temperature provided the kinetically controlled azolyltetrahydropyrimidine derivative **4b** (Scheme 4), while high-temperature treatment of the same starting materials (HOAc, refluxing at 118 °C for 240 min) yielded the thermodynamically preferred dihydropyrazolopyrimidine-5-carboxylic acid **7** (Scheme 5).

**Scheme 6:**
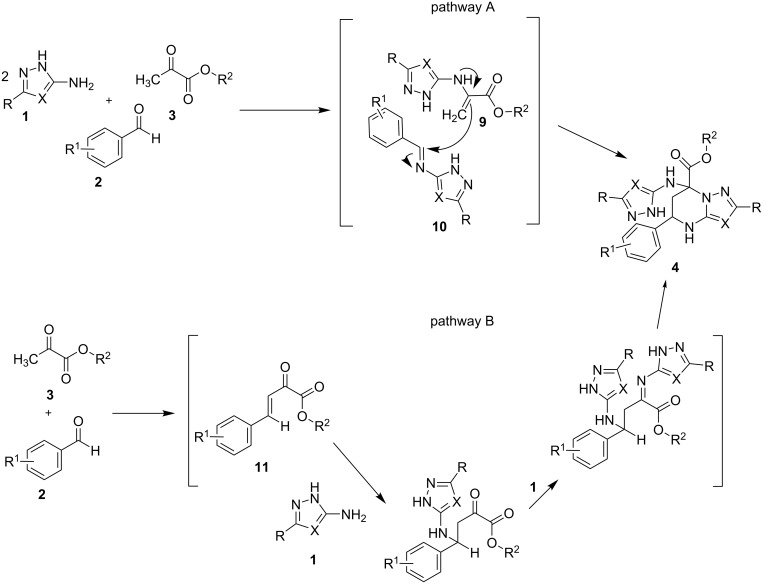
Proposed reaction mechanism.

The most probable pathway A (Scheme 6) to compounds **4** includes initial formation of the corresponding azomethines **9** and **10**. A similar pathway was previously described and discussed in publications [31–32]. We cannot exclude other mechanisms, for example, via arylidenepyruvic acids (esters) **11** formed by water elimination from appropriate aldols (pathway B). However, our attempts to synthesize compound **4** by direct reaction of unsaturated acid **8** and aminoazole **1a** under different conditions (ultrasonic activation and conventional heating) were unsuccessful, and only dihydropyrimidine **7** was obtained.

### Structure elucidation

The purity and structures of the synthesized heterocycles were determined using elemental analysis, mass spectrometry, NMR spectroscopy (1D and 2D NMR experiments), and X-ray diffraction analysis. The signals in the ^1^H and ^13^C NMR spectra of some of the compounds **4** were duplicated. This could have been caused by two reasons, namely by free rotation of the azolyl fragment in the position 7 or by the presence of a second diastereomer. To establish the reason of duplication, NMR spectra were recorded at different temperatures. As the temperature was increased from 25 °C to 130 °C, the peaks were still duplicated, proving the presence of two diastereomers.

The ^1^H NMR spectra of **4a**–**o** exhibited three signals of an AMX system for the tetrahydropyrimidine ring (about 2.27–2.48 ppm, 3.03–3.25 ppm, and 4.52–4.83 ppm), three signals for protons of the NH groups (ca. 7.85–8.13 ppm for the pyrimidine NH, 6.90–7.21 ppm for the NH pyrazolyl moiety, and 11.79–12.07 ppm for the aminopyrazole moiety), a CH signal of the proton at position 2 of the pyrazolopyrimidine, signals of the aryl ring at 7.54–8.34 ppm, and signals corresponding to the alkyl substituent.

The ^1^H NMR spectra of **4a**–**c**/**j**/**k**/**o** showed a double set of signals for the AMX system of the tetrahydropyrimidine ring, the NH protons, and the protons of the CH_2_CH_3_ moiety (in the case of esters) due to the presence of two isomers of **4**.

The ^1^H NMR and ^13^C NMR spectra of triazolyl derivatives **4p**–**u** were more complicated, both due to the duplication of the signals and the fast decomposition of the compounds **4p**–**u** in solutions. Therefore, 2D NMR and, in some cases, ^13^C NMR spectra were overcrowded and uninformative. Particularly, tetrahydropyrimidines **4s**–**u** with hydroxy substituent in the aryl ring happened to be less stable than the derivatives **4p**–**r** with the methoxy group; this explains the absence of ^13^C NMR spectra for compounds **4t**/**u** in our work.

The ^1^H NMR spectra of the products **4p**–**u** exhibited a broad singlet for the triazole NH and carboxyl groups at ca. 13.10–13.30 ppm, a broad singlet for the phenolic OH group at 9.40–9.70 ppm (for compounds **4s**–**u**), singlets of pyrimidine NH and CH groups of the triazolyl and triazole fragments in the interval of 7.31–7.91 ppm, a singlet of the triazolylamino group at 7.23–7.35 ppm, a multiplet for the CH proton in position 5 at 5.10–5.25 ppm (for the derivatives **4r**/**u** with *ortho*-substituent in the aryl ring) or at 4.60–4.90 ppm for the compounds **4p**/**q**/**s**/**t**, multiplets for the CH_2_ group in position 6 at 2.90–3.60 ppm and 2.11–2.50 ppm, and multiplets of aromatic protons at 6.65–7.45 ppm.

**Figure 1:**
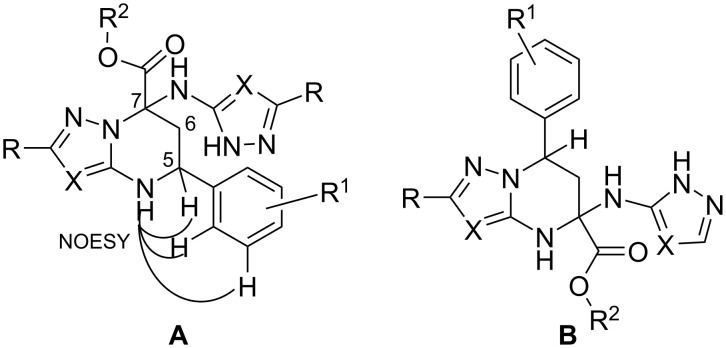
Alternative structures **A** and **B** for the tetrahydroazolopyrimidines **4**.

The spectral data obtained for the tetrahydropyrimidines **4** most likely corresponded to the possible regioisomers **A** and **B** (Figure 1). Additional NOESY experiments, in particular for compound **4k**, showed the presence of cross-peaks between the CH proton at position 5 with the pyrimidine NH group and cross-peaks of the NH group with protons of the aromatic system, thus excluding structure **B** (Figure 1).

**Figure 2:**
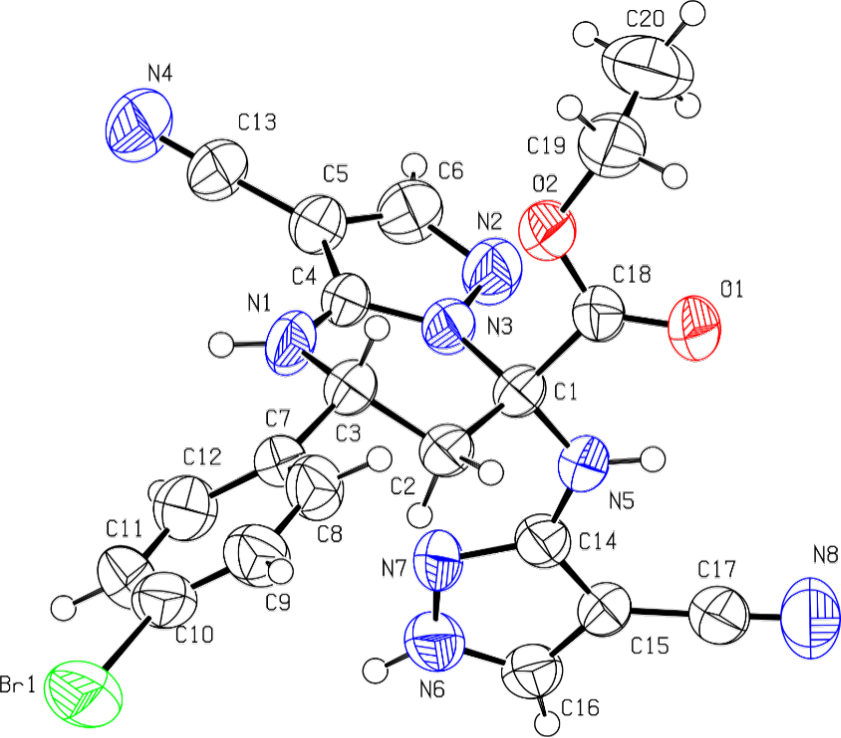
Molecular structure of ethyl 5-(4-bromophenyl)-3-cyano-7-((4-cyano-1*H*-pyrazol-5-yl)amino)-4,5,6,7-tetrahydropyrazolo[1,5-*a*]pyrimidine-7-carboxylate (**4g**) obtained from X-ray diffraction data.

Eventually, the structure of tetrahydropyrimidines **4** was proven by X-ray analysis carried out for a single crystal of one diastereomer of compound **4g**, which allowed assignment of the structure as ethyl 5-(4-bromophenyl)-3-cyano-7-((4-cyano-1*H*-pyrazol-5-yl)amino)-4,5,6,7-tetrahydropyrazolo[1,5-*a*]pyrimidine-7-carboxylate (Figure 2).

Compound **4g** existed as a solvate with acetonitrile in the crystal lattice. The tetrahydropyrimidine ring adopted a half-chair conformation (the puckering parameters [36] were: S = 0.70, Θ = 25.6°, Ψ = 21.6°). Deviations of the C2 and C3 atoms from the mean-plane of the remaining atoms of this ring were −0.26 Å and 0.35 Å, respectively. The phenyl substituent was located in an equatorial position and was slightly turned in relation to the N1–C3 endocyclic bond (the C4–N1–C3–C7 and N1–C3–C7–C12 torsion angles were −163.6(4)° and 19.7(7)°, respectively). Short intramolecular contacts appeared: the H1(N)···H12 distance was 2.10 Å and the van der Waals radii sum [37] was 2.34 Å, the H···N1 distance was 2.55 Å (and the van der Waals radii sum was 2.67 Å), while the H1···C12 distance was 2.60 Å, with a van der Waals radii sum of 2.87 Å. The two vicinal substituents at the C1 atom had different orientations in relation to the partially saturated cycle: the ester substituent was in an axial position, while the other substituent was found in an equatorial position (the C4–N3–C1–C18 and C4–N3–C1–N5 torsion angles were −104.0(5)° and 139.6(5)°, respectively). The carboxylic acid fragment was turned in relation to the N3–C1 endocyclic bond (the N3–C1–C18–O1 torsion angle was −131.4(5)°). The ethyl group was located in an *ap* position to the C1–C18 bond and was almost orthogonal to the C18–O2 bond (the C1–C18–O2–C19 and C18–O2–C19–C20 torsion angles were −175.0(4)° and 85.1(7)°, respectively). The planar cyanopyrazolimino substituent was turned significantly to the N3–C1 endocyclic bond (the N3–C1–N5–C14 torsion angle was −57.5(6)°).

**Figure 3:**
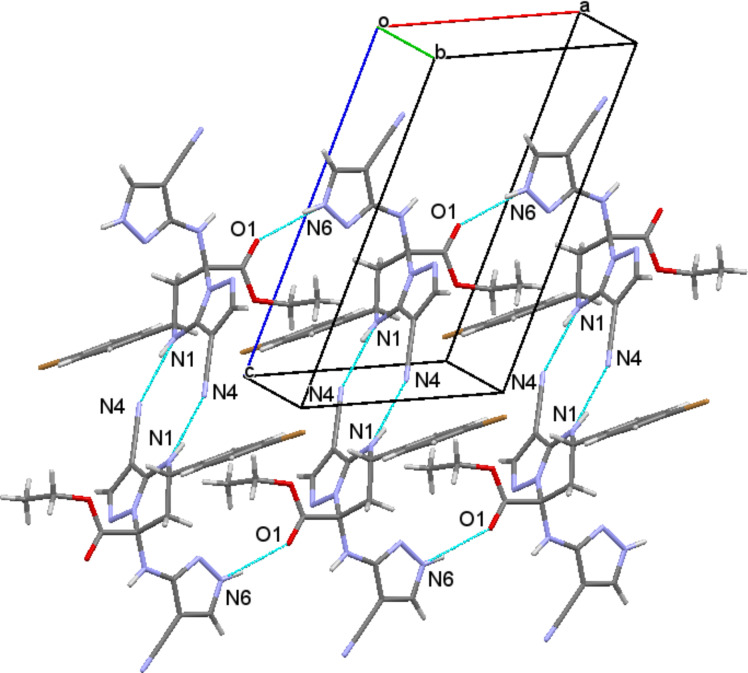
Chains of **4g** molecules in the crystal phase.

In the crystal phase, molecules **4g** formed centrosymmetric dimers due to the N1–H···N4 intermolecular hydrogen bonds (1 x, 1 y, 2 z; H···N 2.22 Å, N–H···N 164°). The dimers were bound by a N6–H···O1 intermolecular hydrogen bond (x 1, y, z; H···O 2.16 Å, N–H···O 162°), forming chains along the [100] crystallographic direction (Figure 3).

## Conclusion

In this study we disclosed a new direction for the multicomponent reaction of 5-aminopyrazole-4-carbonitriles or 3-amino-1,2,4-triazole with pyruvic acid or ethyl pyruvate and aromatic aldehydes under ultrasonication, leading to 7-azolylamino-4,5,6,7-tetrahydroazolo[1,5-*a*]pyrimidines via a cascade of elementary stages that is unusual for such transformations. Thus, we extended the molecular diversity of the compounds obtained by introducing an additional azolyl substituent to the pyrimidine ring.

## Supporting Information

File 1 Experimental and analytical data as well as X-ray crystallographic information.

File 2 ^1^H and ^13^C NMR spectra.
