# Supplementary material for: Ultrasonic-assisted unusual four-component synthesis of 7-azolylamino-4,5,6,7-tetrahydroazolo[1,5-a]pyrimidines
Source: Beilstein J Org Chem. 2020 Feb 27;16:281–9. doi: 10.3762/bjoc.16.27 (PMC7059450; doi:10.3762/bjoc.16.27)
Supplement: File 1 — Experimental and analytical data as well as X-ray crystallographic information. [file Beilstein_J_Org_Chem-16-281-s001.pdf]

BEILSTEIN JOURNAL OF ORGANIC CHEMISTRY

Supporting Information

for

Ultrasonic-assisted unusual four-component synthesis of 7-azolylamino-4,5,6,7-tetrahydroazolo[1,5-*a*]pyrimidines

Yana I. Sakhno, Maryna V. Murlykina, Oleksandr I. Zbruyev, Anton V. Kozyryev, Svetlana V. Shishkina, Dmytro Sysoiev, Vladimir I. Musatov, Sergey M. Desenko and Valentyn A. Chebanov

Beilstein J. Org. Chem. **2020,** *16,* 281–289. doi:10.3762/bjoc.16.27

Experimental and analytical data as well as X-ray crystallographic information

License and Terms: This is a supporting information file under the terms of the Creative Commons Attribution License (https://creativecommons.org/licenses/by/4.0). Please note that the reuse, redistribution and reproduction in particular requires that the authors and source are credited.
The license is subject to the *Beilstein Journal of Organic Chemistry* terms and conditions: (https://www.beilstein-journals.org/bjoc)

General

All solvents were obtained from standard commercial vendors and used without additional purification. Starting 5-aminopyrazoles **1a**/**b** and (*p*-methoxybenzylidene)pyruvic acid (**8**) were synthesized according to described procedures [1,2]. 3-Amino-1,2,4-triazole (**1c**), pyruvic acid (**3a**), and ethyl 2-oxopropanoate (**3b**) are commercially available.

Ultrasonication was carried out with the help of a standard US bath (SELDI, Ukraine), producing irradiation at 44.2 kHz in a round-bottom flask equipped with a condenser. Melting points of all the compounds synthesized were determined with a Kofler melting point apparatus and are uncorrected. The ^{1}H, ^{13}C, and 2D NMR experiments were performed in DMSO-d_6 at 400 MHz (100 MHz for ^{13}C) with a Varian MR-400 spectrometer, at 300 MHz (75 MHz for ^{13}C) with a Bruker Advance 300 spectrometer, and at 600 MHz (150 MHz for ^{13}C) with a Bruker Advance DRX600 spectrometer. The mass spectra were measured on a GS-MS Varian 1200L (direct input of the sample, ionizing voltage 70 eV) instrument and on a LCQ Advantage system for LC–MS. Elemental analysis was performed on a Euro Vector EA-3000 and Heraeus (CHN-O-RAPID) CHN analyzer. Samples for elemental analysis and for measuring the melting temperature were prepared separately.

Chemistry

3-Cyano-7-((4-cyano-1H-pyrazol-5-yl)amino)-5-phenyl-4,5,6,7-tetrahydropyrazolo[1,5-a]pyrimidine-7-carboxylic acid (**4a**). 5-Amino-1*H*-pyrazole-4-carbonitrile (**1a**, 0.92 mmol) was dissolved in 1.5 mL of acetic acid, followed by benzaldehyde (**2a**, 0.46 mmol) and pyruvic acid (**3a**, 0.46 mmol). The mixture was ultrasonicated for 90 min and allowed to stand until a precipitate formed. The precipitate was filtered off and dried in vacuum.

White solid (0.13 g, 76%); [Found: C, 57.52; H, 3.81; N, 29.99. $C_{18}H_{14}N_8O_2$ requires C, 57.57; H, 3.77; N, 29.93%]; The ^{1}H NMR, δ_H, (400 MHz, DMSO-*d6*) (two stereoisomers): **A** stereoisomer (70% mol.): 2.33-2.40 (m, 1H, CH), 3.16-3.25 (m, 1H, CH), 4.69-4.78 (m, 1H, CH), 6.71 (br s, 1H, NH), 7.29-7.47 (m, 5H, Ar), 7.55 (s, 1H, CH), 7.99 (s, 1H, NH), 8.19 (s, 1H, CH), 12.76 (br s, 1H, NH); **B** stereoisomer (30% mol.): 2.40-2.49 (m, 1H, CH), 3.03-3.11 (m, 1H, CH), 4.69-4.78 (m, 1H, CH), 6.96 (br s, 1H, NH), 7.29-7.47 (m, 5H, Ar), 7.69 (s, 1H, CH), 7.99 (s, 1H, NH), 8.34 (s, 1H, CH), 12.76 (br s, 1H, NH); The ^{13}C NMR, δ_C (100 MHz, DMSO-*d6*) **A** stereoisomer: 170.3, 153.3, 152.1, 141.3, 141.2, 135.4, 129.0, 128.4, 127.2, 115.2, 114.5, 79.0, 73.4, 73.0, 53.4, 37.8; **B** stereoisomer: 169.2, 152.9, 151.5, 142.0, 141.0, 135.2, 129.0, 128.4, 127.3, 115.0, 114.8, 78.9, 71.6, 71.2, 51.3, 38.8. ESI MS, m/z (%): negative mode: 373 (100) $[M-H]^-$.

The compounds **4b**/**c** were obtained according to the procedure for **4a**:

3-Cyano-7-((4-cyano-1H-pyrazol-5-yl)amino)-5-(4-methoxyphenyl)-4,5,6,7-tetrahydropyrazolo[1,5-a]pyrimidine-7-carboxylic acid (**4b**). White solid (0.16 g, 86%); [Found: C, 56.37; H, 4.11; N, 27.76. $C_{19}H_{16}N_8O_3$ requires C, 56.43; H, 3.99; N, 27.71%]; The ^{1}H NMR, δ_H, (400 MHz, DMSO-*d6*) (two stereoisomers): **A** stereoisomer (70% mol.): 2.27-2.34 (m, 1H, CH), 3.12-3.23 (m, 1H, CH), 3.75 (s, 3H, OCH_3), 4.61-4.74 (m, 1H, CH), 6.69 (br s, 1H, NH), 6.90-7.38 (m, 4H, Ar), 7.54 (s, 1H, CH), 7.91 (s, 1H, NH), 8.19 (s, 1H, CH), 12.76 (br s, 1H, NH); **B** stereoisomer (30% mol.): 2.41-2.47 (m, 1H, CH), 2.96-3.06 (m, 1H, CH), 3.86 (s, 3H, OCH_3), 4.61-4.74 (m, 1H, CH), 6.90-7.38 (m, 4H, Ar), 7.68 (s, 1H, CH), 7.91 (s, 1H, NH), 8.26 (s, 1H, CH), 12.77 (br s, 1H, NH); The ^{13}C NMR, δ_C (100 MHz, DMSO-*d6*) **A** stereoisomer: 169.5, 158.8, 152.5, 141.3, 140.6, 135.6, 134.4, 132.4, 127.8, 127.7, 113.7, 81.6, 72.8, 72.4, 55.0, 52.2, 37.2; **B** stereoisomer: 169.2, 158.7, 152.5, 151.5, 150.9, 141.3, 140.8, 135.5, 132.1, 127.9, 127.7, 114.3, 72.4, 71.0, 55.0, 50.0, 38.4. ESI MS, m/z (%): negative mode: 403 (100) $[M-H]^-$.

5-(4-Chlorophenyl)-3-cyano-7-((4-cyano-1H-pyrazol-5-yl)amino)-4,5,6,7-tetrahydropyrazolo[1,5-a]pyrimidine-7-carboxylic acid (**4c**). White solid (0.14 g, 75%); [Found: C, 52.78; H, 3.27; N, 27.50. $C_{18}H_{13}ClN_8O_2$ requires C, 52.89; H, 3.21; N, 27.41%]; The ^{1}H NMR, δ_H, (400 MHz, DMSO-*d6*) (two stereoisomers): **A** stereoisomer (75% mol.): 2.33-2.42 (m, 1H, CH), 3.12-3.23 (m, 1H, CH), 4.69-4.83 (m, 1H, CH), 6.69 (br s, 1H, NH), 7.35-7.53 (m, 4H, Ar), 7.56 (s, 1H, CH), 7.99 (s, 1H, NH), 8.19 (s, 1H, CH), 12.75 (br s, 1H, NH); **B** stereoisomer (25% mol.): 2.42-2.48 (m, 1H, CH), 3.03-3.11 (m, 1H, CH), 4.69-4.83 (m, 1H, CH), 6.92 (br s, 1H, NH), 7.35-7.53 (m, 4H, Ar), 7.69 (s, 1H, CH), 7.99 (s, 1H, NH), 8.33 (s, 1H, CH), 12.75 (br s, 1H, NH); The ^{13}C NMR, δ_C (100 MHz, DMSO-*d6*) **A** stereoisomer: 169.6, 151.5, 140.7, 139.7, 134.9, 134.6, 132.3, 128.6, 128.3, 114.5, 113.9, 81.6, 72.7, 72.4, 52.2, 36.9; **B** stereoisomer: 168.6, 152.7, 141.4, 139.5, 134.8, 134.7, 132.1, 128.8, 128.7, 114.3, 114.1, 78.5, 71.1, 71.0, 50.2, 38.1. ESI MS, m/z (%): negative mode: 407 (100) $[M-H]^-$.

Ethyl 3-cyano-7-((4-cyano-1H-pyrazol-5-yl)amino)-5-phenyl-4,5,6,7-tetrahydropyrazolo[1,5-a]pyrimidine-7-carboxylate (**4d**). 5-Amino-1*H*-pyrazole-4-carbonitrile (**1a**, 0.92 mmol) was dissolved in 1.5 mL of acetic acid, followed by benzaldehyde (**2a**, 0.46 mmol) and ethyl pyruvate (**3b**, 0.46 mmol). The mixture was ultrasonicated for 2 hours and allowed to stand until a precipitate formed. The precipitate was filtered off and dried in vacuum.

White solid (0.11 g, 60%), m.p. 198-200 °C; [Found: C, 59.60; H, 4.55; N, 27.91. $C_{20}H_{18}N_8O_2$ requires C, 59.69; H, 4.51; N, 27.85%]; The ^{1}H NMR, δ_H, (400 MHz, DMSO-*d6*): 1.25 (t, *J* 7.3 Hz, 3H, $OCH_2C\underline{H}_3$), 2.38-2.48 (m, 1H, CH), 3.11-3.25 (m, 1H, CH), 4.29 (q, *J* 7.3 Hz, 2H, $C\underline{H}_2CH_3$), 4.55-4.63 (m, 1H, CH), 7.14 (br s, 1H, NH), 7.28-7.50 (m, 5H, Ar), 7.59 (s, 1H, CH), 8.06 (s, 1H, NH), 8.21 (s, 1H, CH) 12.35 (br s, 1H,

NH); The ^{13}C NMR, δ_C (150 MHz, DMSO-*d6*): 168.7, 153.8, 151.8, 141.3, 141.0, 135.3, 129.1, 128.5, 127.2, 115.3, 114.7, 78.9, 74.1, 71.5, 63.1, 53.2, 38.7, 14.3. MS, m/z (%): negative mode: 401 (100) $[M-H]^-$.

The compounds **4e**–**o** were obtained similarly to the **4d**:

Ethyl 3-cyano-7-((4-cyano-1H-pyrazol-5-yl)amino)-5-(4-methoxyphenyl)-4,5,6,7-tetrahydropyrazolo[1,5-a]pyrimidine-7-carboxylate (**4e**). White solid (0.15 g, 73%), m.p. 144-146 °C; [Found: C, 58.29; H, 4.72; N, 25.98. $C_{21}H_{20}N_8O_3$ requires C, 58.33; H, 4.66; N, 25.91%]; The ^1H NMR, δ_H, (400 MHz, DMSO-*d6*): 1.23 (t, *J* 7.3 Hz, 3H, $OCH_2C\underline{H}_3$), 2.39 (dd, ^2J 2.2 Hz, ^3J 12.2 Hz, 1H, CH), 3.11-3.21 (m, 1H, CH), 3.77 (s, 3H, OCH_3), 4.21-4.23 (m, 2H, $C\underline{H}_2CH_3$), 4.52 (dd, ^3J 2.2 Hz, ^3J 12.2 Hz, 1H, CH), 6.92-7.38 (m, 4H, Ar), 7.13 (br s, 1H, NH), 7.57 (s, 1H, CH), 7.97 (s, 1H, NH), 8.20 (s, 1H, CH) 12.71 (br s, 1H, NH); The ^{13}C NMR, δ_C (150 MHz, DMSO-*d6*): 168.7, 159.6, 151.7, 141.3, 141.2, 135.3, 132.8, 128.4, 115.3, 114.7, 114.4, 78.9, 74.1, 71.3, 63.0, 55.7, 52.6, 38.7, 14.3. MS, m/z (%): negative mode: 431 (100) $[M-H]^-$.

Ethyl 5-(4-chlorophenyl)-3-cyano-7-((4-cyano-1H-pyrazol-5-yl)amino)-4,5,6,7-tetrahydropyrazolo[1,5-a]pyrimidine-7-carboxylate (**4f**). White solid (0.11 g, 55%), m.p. 146-148 °C; [Found: C, 54.91; H, 3.95; N, 25.72. $C_{20}H_{17}ClN_8O_2$ requires C, 54.99; H, 3.92; N, 25.65%]; The ^1H NMR, δ_H, (400 MHz, DMSO-*d6*): 1.24 (t, *J* 7.1 Hz, 3H, $OCH_2C\underline{H}_3$), 2.37-2.48 (m, 1H, CH), 3.09-3.24 (m, 1H, CH), 4.22-4.34 (m, 2H, $C\underline{H}_2CH_3$), 4.55-4.66 (m, 1H, CH), 7.14 (br s, 1H, NH), 7.40-7.53 (m, 4H, Ar), 7.59 (s, 1H, CH), 8.06 (s, 1H, NH), 8.20 (s, 1H, CH) 12.73 (br s, 1H, NH); The ^{13}C NMR, δ_C (150 MHz, DMSO-*d6*): 168.6, 153.7, 151.6, 141.3, 140.0, 135.5, 132.9, 129.1, 129.0, 115.2, 114.7, 78.9, 73.9, 71.5, 63.1, 52.5, 38.3, 14.3. MS, m/z (%):negative mode: 435 (100) $[M-H]^-$.

Ethyl 5-(4-bromophenyl)-3-cyano-7-((4-cyano-1H-pyrazol-5-yl)amino)-4,5,6,7-tetrahydropyrazolo[1,5-a]pyrimidine-7-carboxylate (**4g**). White solid (0.18 g, 80%), m.p. 152-154 °C; [Found: C, 49.82; H, 3.62; N, 23.33. $C_{20}H_{17}BrN_8O_2$ requires C, 49.91; H, 3.56; N, 23.28%]; The ^{1}H NMR, δ_H, (400 MHz, DMSO-*d6*): 1.24 (t, *J* 7.1 Hz, 3H, $OCH_2C\underline{H}_3$), 2.38-2.48 (m, 1H, CH), 3.11-3.23 (m, 1H, CH), 4.19-4.36 (m, 2H, $C\underline{H}_2CH_3$), 4.55-4.64 (m, 1H, CH), 7.14 (br s, 1H, NH), 7.32-7.61 (m, 4H, Ar), 7.62 (s, 1H, CH), 8.06 (s, 1H, NH), 8.19 (s, 1H, CH) 12.71 (br s, 1H, NH); The ^{13}C NMR, δ_C (150 MHz, DMSO-*d6*): 168.6, 153.7, 151.6, 141.3, 140.0, 135.5, 131.9, 129.1, 129.5, 121.4, 115.2, 114.7, 78.9, 73.9, 71.5, 63.1, 52.5, 38.2, 14.3. MS, m/z (%): 479 (100) $[M-H]^-$.

Ethyl 3-cyano-7-((4-cyano-1H-pyrazol-5-yl)amino)-5-(4-(methoxycarbonyl)phenyl)-4,5,6,7-tetrahydropyrazolo[1,5-a]pyrimidine-7-carboxylate (**4h**). White solid (0.135 g, 64%), m.p. 205-207 °C; [Found: C, 57.30; H, 4.46; N, 24.39. $C_{22}H_{20}N_8O_4$ requires C, 57.39; H, 4.38; N, 24.34%]; The ^{1}H NMR, δ_H, (400 MHz, DMSO-*d6*): 1.24 (t, *J* 7.3 Hz, 3H, $OCH_2C\underline{H}_3$), 2.38 (dd, ^3J 2.2 Hz, ^2J 12.2 Hz, 1H, CH), 3.07-3.25 (m, 1H, CH), 3.77 (s, 3H, OCH_3), 4.18-4.36 (m, 2H, $C\underline{H}_2CH_3$), 4.52 (dd, ^3J 2.2 Hz, ^3J 12.2 Hz, 1H, CH), 6.92-7.41 (m, 4H, Ar), 7.12 (br s, 1H, NH), 7.56 (s, 1H, CH), 7.97 (s, 1H, NH), 8.21 (s, 1H, CH) 12.72 (br s, 1H, NH); The ^{13}C NMR, δ_C (150 MHz, DMSO-*d6*): 172.5, 168.7, 159.5, 153.8, 151.7, 141.2, 135.3, 132.8, 128.4, 115.3, 114.7, 114.4, 78.9, 74.1, 71.3, 63.0, 55.7, 52.6, 38.7, 14.3. ESI MS, m/z (%): negative mode: 431 (100) $[M-C_2H_5]^-$.

Ethyl 3-cyano-7-((4-cyano-1H-pyrazol-5-yl)amino)-5-(4-cyanophenyl)-4,5,6,7-tetrahydropyrazolo[1,5-a]pyrimidine-7-carboxylate (**4i**). White solid (0.1 g, 53%), m.p. 159-161 °C; [Found: C, 58.96; H, 4.11; N, 29.53. $C_{21}H_{17}N_9O_2$ requires C, 59.01; H, 4.01; N, 29.49%]; The ^{1}H NMR, δ_H, (400 MHz, DMSO-*d6*): 1.23 (t, *J* 7.1 Hz, 3H, $OCH_2C\underline{H}_3$), 2.45-2.48 (m, 1H, CH), 3.13-3.25 (m, 1H, CH), 4.20-4.34 (m, 2H, $C\underline{H}_2CH_3$), 4.64-4.76 (m,

1H, CH), 7.12 (br s, 1H, NH), 7.61 (s, 1H, CH), 7.62-7.91 (m, 4H, Ar), 8.15 (s, 1H, NH), 8.19 (s, 1H, CH) 12.71 (br s, 1H, NH); The ^{13}C NMR, δ_C (150 MHz, DMSO-*d6*): 168.4, 153.6, 151.5, 146.7, 141.3, 135.3, 133.0, 128.3, 119.1, 115.1, 114.6, 111.2, 78.9, 73.8, 71.7, 63.1, 52.7, 37.7, 14.3. MS (EI, 70 eV), m/z (%): 222 (85), 246 (54), 427 (5) $[M]^+$.

Ethyl 3-cyano-7-((4-cyano-3-methyl-1H-pyrazol-5-yl)amino)-2-methyl-5-phenyl-4,5,6,7-tetrahydropyrazolo[1,5-a]pyrimidine-7-carboxylate (**4j**). White solid (0.08 g, 45%); [Found: C, 61.22; H, 5.25; N, 26.05. $C_{22}H_{22}N_8O_2$ requires C, 61.38; H, 5.15; N, 26.03%]; The ^{1}H NMR, δ_H, (400 MHz, DMSO-*d6*) (two stereoisomers): **A** stereoisomer (80% mol.): 1.07 (t, *J* 7.1 Hz, 3H, $OCH_2C\underline{H}_3$), 2.12 (s, 1H, CH_3), 2.27 (s, 1H, CH_3), 2.41-2.47 (m, 1H, CH), 3.22-3.29 (m, 1H, CH), 4.04 (q, *J* 7.1 Hz, 2H, $C\underline{H}_2CH_3$), 4.61-4.68 (m, 1H, CH), 7.04 (br s, 1H, NH), 7.29-7.45 (m, 5H, Ar), 8.25 (s, 1H, NH), 12.40 (br s, 1H, NH); **B** stereoisomer (20% mol.): 1.24 (t, *J* 6.9 Hz, 3H, $OCH_2C\underline{H}_3$), 2.06 (s, 1H, CH_3), 2.23 (s, 1H, CH_3), 2.32-2.39 (m, 1H, CH), 3.14-3.20 (m, 1H, CH), 4.22-4.30 (m, 2H, $C\underline{H}_2CH_3$), 4.55-4.61 (m, 1H, CH), 6.89 (br s, 1H, NH), 7.29-7.45 (m, 5H, Ar), 7.95 (s, 1H, NH), 12.46 (br s, 1H, NH); The ^{13}C NMR, δ_C (150 MHz, DMSO-*d6*) **A** stereoisomer: 167.6, 153.7, 151.5, 150.2, 145.9, 141.1, 129.0, 128.3, 127.3, 115.3, 115.1, 79.1, 73.4, 71.5, 62.2, 50.9, 37.2, 14.2, 13.2, 10.5. MS, m/z (%): negative mode 429 (100) $[M-H]^-$.

Ethyl 3-cyano-7-((4-cyano-3-methyl-1H-pyrazol-5-yl)amino)-5-(4-methoxyphenyl)-2-methyl-4,5,6,7-tetrahydropyrazolo[1,5-a]pyrimidine-7-carboxylate (**4k**). White solid (0.13 g, 68%); [Found: C, 59.82; H, 5.37; N, 24.23. $C_{23}H_{24}N_8O_3$ requires C, 59.99; H, 5.25; N, 24.33%]; The ^{1}H NMR, δ_H, (400 MHz, DMSO-*d6*) (two stereoisomers): **A** stereoisomer (65% mol.): 1.07 (t, *J* 7.3 Hz, 3H, $OCH_2C\underline{H}_3$), 2.12 (s, 1H, CH_3), 2.26 (s, 1H, CH_3), 2.43 (dd, ^2J 13.2 Hz, ^3J 2.6 Hz, 1H, CH), 3.15 (dd, ^2J 13.2 Hz, ^3J 11.4 Hz, 1H, CH), 3.75 (s, 3H, OCH_3), 4.07 (q, *J* 7.3 Hz, 2H, $C\underline{H}_2CH_3$), 4.59 (dd, ^3J 2.6 Hz, ^3J 11.4 Hz, 1H, CH),

6.91-7.30 (m, 4H, Ar), 7.06 (br s, 1H, NH), 8.20 (s, 1H, NH), 12.35 (br s, 1H, NH); **B** stereoisomer (35% mol.): 1.23 (t, *J* 7.3 Hz, 3H, $OCH_2C\underline{H}_3$), 2.06 (s, 1H, CH_3), 2.23 (s, 1H, CH_3), 2.34 (dd, ^2J 12.9 Hz, ^3J 2.9 Hz, 1H, CH), 3.15 (dd, ^2J 12.9 Hz, ^3J 12.4 Hz, 1H, CH), 3.76 (s, 3H, OCH_3), 4.23-4.28 (m, 2H, $C\underline{H}_2CH_3$), 4.53 (dd, ^3J 2.9 Hz, ^3J 12.4 Hz, 1H, CH), 6.91 (br s, 1H, NH), 6.95-7.33 (m, 4H, Ar), 7.87 (s, 1H, NH), 12.35 (br s, 1H, NH); The ^{13}C NMR, δ_C (150 MHz, DMSO-*d6*) **A** stereoisomer: 167.7, 159.4, 153.4, 151.5, 150.3, 146.2, 132.8, 128.5, 115.3, 115.1, 114.3, 79.0, 73.5, 71.4, 62.3, 55.6, 50.3, 37.3, 14.2, 13.2, 10.6; **B** stereoisomer: 168.8, 159.5, 153.4, 152.2, 150.4, 145.8, 133.0, 128.4, 115.5, 114.9, 114.4, 78.9, 73.5, 71.8, 63.0, 54.7, 52.5, 38.4, 14.3, 13.3, 10.8. MS, m/z (%): negative mode 459 (100) $[M-H]^-$.

Ethyl 5-(4-chlorophenyl)-3-cyano-7-((4-cyano-3-methyl-1H-pyrazol-5-yl)amino)-2-methyl-4,5,6,7-tetrahydropyrazolo[1,5-a]pyrimidine-7-carboxylate (**4l**). White solid (0.105 g, 55%), m.p. 151-153 °C; [Found: C, 56.75; H, 4.62; N, 24.18. $C_{22}H_{21}ClN_8O_2$ requires C, 56.84; H, 4.55; N, 24.10%]; The ^{1}H NMR, δ_H, (400 MHz, DMSO-*d6*): 1.23 (t, *J* 7.1 Hz, 3H, $OCH_2C\underline{H}_3$), 2.07 (s, 1H, CH_3), 2.23 (s, 1H, CH_3), 2.39 (dd, ^3J 2.7 Hz, ^2J 12.2 Hz, 1H, CH), 3.09-3.22 (m, 1H, CH), 4.26 (q, *J* 7.1 Hz, 2H, $C\underline{H}_2CH_3$), 4.61 (dd, ^3J 2.7 Hz, ^3J 12.2 Hz, 1H, CH), 6.90 (br s, 1H, NH), 7.38-7.50 (m, 4H, Ar), 7.96 (s, 1H, NH), 12.44 (br s, 1H, NH); The ^{13}C NMR, δ_C (150 MHz, DMSO-*d6*): 168.7, 153.3, 152.1, 149.7, 145.7, 140.1, 132.9, 129.1, 129.0, 115.4, 114.8, 79.1, 73.3, 72.0, 63.1, 52.4, 37.9, 14.3, 13.3, 10.6. MS, m/z (%): negative mode 463 (100) $[M-H]^-$.

Ethyl 5-(4-bromophenyl)-3-cyano-7-((4-cyano-3-methyl-1H-pyrazol-5-yl)amino)-2-methyl-4,5,6,7-tetrahydropyrazolo[1,5-a]pyrimidine-7-carboxylate (**4m**). White solid (0.13 g, 63%), m.p. 162-164 °C; [Found: C, 51.81; H, 4.12; N, 22.11. $C_{22}H_{21}BrN_8O_2$ requires C, 51.88; H, 4.16; N, 22.0%]; The ^{1}H NMR, δ_H, (400 MHz, DMSO-*d6*): 1.23 (t, *J* 7.1 Hz, 3H,

$OCH_2C\underline{H}_3$), 2.07 (s, 1H, CH_3), 2.23 (s, 1H, CH_3), 2.41 (dd, ^3J 2.7 Hz, ^2J 11.7 Hz, 1H, CH), 3.10-3.21 (m, 1H, CH), 4.26 (q, *J* 7.1 Hz, 2H, $C\underline{H}_2CH_3$), 4.59 (dd, ^3J 2.7 Hz, ^3J 11.7 Hz, 1H, CH), 6.88 (br s, 1H, NH), 7.33-7.63 (m, 4H, Ar), 7.96 (s, 1H, NH), 12.45 (br s, 1H, NH); The ^{13}C NMR, δ_C (150 MHz, DMSO-*d6*): 168.7, 153.4, 152.1, 149.6, 145.7, 140.6, 131.9, 129.4, 121.4, 115.4, 114.8, 79.1, 73.3, 72.0, 63.1, 52.4, 37.8, 14.3, 13.3, 10.5. MS, m/z (%): 509 (100) $[M+H]^+$.

Ethyl 3-cyano-7-((4-cyano-3-methyl-1H-pyrazol-5-yl)amino)-5-(4-(methoxycarbonyl)phenyl)-2-methyl-4,5,6,7-tetrahydropyrazolo[1,5-a]pyrimidine-7-carboxylate (**4n**). White solid (0.11 g, 55%), m.p. 160-162 °C; [Found: C, 58.92; H, 5.11; N, 23.07. $C_{24}H_{24}N_8O_4$ requires C, 59.01; H, 4.95; N, 22.94%]; The ^1H NMR, δ_H, (400 MHz, DMSO-*d6*): 1.24 (t, *J* 7.1 Hz, 3H, $OCH_2C\underline{H}_3$), 2.07 (s, 1H, CH_3), 2.22 (s, 1H, CH_3), 2.40-2.48 (m, 1H, CH), 3.12-3.24 (m, 1H, CH), 3.87 (s, 3H, OCH_3), 4.27 (q, *J* 7.1 Hz, 2H, $C\underline{H}_2CH_3$), 4.65-4.73 (m, 1H, CH), 6.91 (br s, 1H, NH), 7.52-8.02 (m, 4H, Ar), 8.04 (s, 1H, NH),12.42 (br s, 1H, NH); The ^{13}C NMR, δ_C (150 MHz, DMSO-*d6*): 168.6, 166.4, 153.3, 152.1, 149.7, 146.6, 145.8, 129.9, 129.7, 127.5, 115.4, 114.8, 79.1, 73.3, 72.1, 63.1, 52.8, 52.6, 37.8, 14.3, 13.3, 10.5. ESI MS, m/z (%): 318 (100), 489 (69) $[M+H]^+$.

Ethyl 3-cyano-7-((4-cyano-3-methyl-1H-pyrazol-5-yl)amino)-5-(4-cyanophenyl)-2-methyl-4,5,6,7-tetrahydropyrazolo[1,5-a]pyrimidine-7-carboxylate (**4o**). White solid (0.09 g, 47%); [Found: C, 60.58; H, 4.57; N, 27.73. $C_{23}H_{21}N_9O_2$ requires C, 60.65; H, 4.65; N, 27.68%]; The ^1H NMR, δ_H, (400 MHz, DMSO-*d6*) (two stereoisomers): **A** stereoisomer (70% mol.): 1.05 (t, *J* 7.1 Hz, 3H, $OCH_2C\underline{H}_3$), 2.13 (s, 1H, CH_3), 2.26 (s, 1H, CH_3), 2.46 (dd, ^3J 2.3 Hz, ^2J 10.2 Hz, 1H, CH), 3.23-3.31 (m, 1H, CH), 3.99 (q, *J* 7.1 Hz, 2H, $C\underline{H}_2CH_3$), 4.78 (dd, ^3J 2.3 Hz, ^3J 10.1 Hz, 1H, CH), 7.16 (br s, 1H, NH), 7.51-7.90 (m, 4H, Ar), 8.34 (s, 1H, NH), 12.26 (br s, 1H, NH); **B** stereoisomer (30% mol.): 1.24 (t, *J* 7.0 Hz, 3H,

$OCH_2C\underline{H}_3$), 2.08 (s, 1H, CH_3), 2.22 (s, 1H, CH_3), 2.47 (dd, ^3J 2.5 Hz, ^2J 11.5 Hz, 1H, CH), 3.15-3.24 (m, 1H, CH), 4.26 (q, J 7.0 Hz, 2H, $C\underline{H}_2CH_3$), 4.72 (dd, ^3J 2.5 Hz, ^3J 11.5 Hz, 1H, CH), 6.90 (br s, 1H, NH), 7.51-7.90 (m, 4H, Ar), 8.06 (s, 1H, NH), 12.26 (br s, 1H, NH); The ^{13}C NMR, δ_C (150 MHz, DMSO-*d6*) **A** stereoisomer: 167.4, 153.4, 151.2, 150.2, 146.8, 146.4, 132.9, 128.4, 119.1, 115.2, 115.0, 111.0, 78.9, 73.2, 71.7, 62.4, 50.7, 36.8, 14.1, 13.2, 10.6; **B** stereoisomer: 168.5, 153.2, 151.9, 149.7, 147.6, 145.1, 132.9, 128.2, 119.1, 115.3, 114.7, 112.6, 79.0, 72.6, 72.1, 63.1, 52.6, 37.2, 14.3, 12.5, 10.5. MS, m/z (%): negative mode: 454 (100) $[M-H]^-$.

7-(1H-1,2,4-Triazol-5-ylamino)-5-(4-methoxyphenyl)-4,5,6,7-tetrahydro-[1,2,4]triazolo[1,5-a]pyrimidine-7-carboxylic acid (**4p**). 3-Amino-1,2,4-triazole (**1c**, 2 mmol) was dissolved in 2.5 mL of acetic acid, followed by 4-methoxybenzaldehyde (**2b**, 1 mmol) and pyruvic acid (**3a**, 1 mmol). The mixture was ultrasonicated for 2 hours. The solvent was evaporated and 6 mL of acetone were added to the crude product. The formed precipitate has to be quickly filtered off using pleated paper filter avoiding water and then dried under vacuum.

White solid (60%); [Found: C, 49.31; H, 4.69; N, 30.67. $C_{15}H_{16}N_8O_3{\cdot}0.5H_2O$ requires: C, 50.02; H, 4.87; N, 30.95 %]; The ^1H NMR, δ_H, (300 MHz, DMSO-*d6*): 2.22-2.43, 3.01-3.24 (m, 2H, CH_2), 3.73 (s, 3H, $C\underline{H_3}O$), 4.69-4.80 (m, 1H, CH), 6.90-7.30 (m, 4H, ArH), 7.35 (s, 1H, NH), 7.51-7.82 (s, 1H, NH; s, 1H, CH; s, 1H, CH); The ^{13}C NMR, δ_c, (75MHz, DMSO-*d6*): 170.2, 169.3, 158.8, 155.3, 154.9, 148.2, 133.2, 127.7, 113.9, 72.0, 55.1, 52.4, 50.7. LC/MS (ESI): 357 $[M+H]^+$.

The compounds **4q**–**u** were obtained similarly to **4p**:

7-(1H-1,2,4-Triazol-5-ylamino)-5-(3-methoxyphenyl)-4,5,6,7-tetrahydro-[1,2,4]triazolo[1,5-a]pyrimidine-7-carboxylic acid (**4q**). White solid (65%); [Found.: C,

45.92; H, 5.14; N, 28.56. $C_{15}H_{16}N_8O_3{\cdot}2H_2O$ requires C, 47.13; H, 4.65; N, 28.68 %]; The ^{1}H NMR, δ_H, (300 MHz, DMSO-*d6*): 3.07-3.27, 2.26-2.45 (m, 2H, CH_2), 3.75 (s, 3H, $C\underline{H_3}O$), 4.74-4.86 (m, 1H, CH), 6.85-7.32 (m, 4H, ArH), 7.35(s, 1H, NH), 7.51-7.91(s, 1H, NH; c, 1H, CH; c, 1H, CH), 12.67 (s, 1H, NH); The ^{13}C NMR, δ_C, (75MHz, DMSO-*d6*): 170.2, 169.2, 159.3, 155.2, 148.1, 142.9, 129.6, 118.7, 113.3, 113.1, 112.2, 111.9, 72.0, 55.0, 52.9, 51.2, 40.0. LC/MS (ESI): 357 $[M+H]^+$.

7-(1H-1,2,4-Triazol-5-ylamino)-5-(2-methoxyphenyl)-4,5,6,7-tetrahydro-[1,2,4]triazolo[1,5-a]pyrimidine-7-carboxylic acid (**4r**). White solid (70%); [Found: C, 48.13; H, 4.85; N, 29.93. $C_{15}H_{16}N_8O_3{\cdot}H_2O$ requires C, 48.21; H, 4.74; N, 26.95 %]; The ^{1}H NMR, δ_H, (300 MHz, DMSO-*d6*): 2.97-3.25, 2.17-2.38 (m, 2H, CH_2), 3.77 (s, 3H, $C\underline{H_3}O$), 5.05-5.13 (m, 1H, CH), 6.93-7.49 (m, 4H, ArH), 7.33 (s, 1H, NH), 7.36-7.74 (s, 1H, CH; s, 1H, NH; c, 1H, CH); The ^{13}C NMR, δ_C, (150MHz, DMSO-*d6*): 172.5, 170.8, 156.6, 156.5, 156.1, 148.6, 129.4, 129.1, 126.8, 121.1, 111.4, 72.8, 56.1, 46.9, 37.4. LC/MS (ESI): 357 $[M+H]^+$.

7-(1H-1,2,4-Triazol-5-ylamino)-5-(4-hydroxyphenyl)-4,5,6,7-tetrahydro-[1,2,4]triazolo[1,5-a]pyrimidine-7-carboxylic acid (**4s**). White solid (76%); [Found: C, 42.42; H, 5.09; N, 28.27. $C_{14}H_{14}N_8O_3{\cdot}3H_2O$ requires C, 40.98; H, 4.92; N, 26.87 %]; The ^{1}H NMR, δ_H, (300 MHz, DMSO-*d6*): 3.01-3.23, 2.22-2.43 (s, 2H, CH_2), 4.64-4.75 (m, 1H, CH), 6.74-7.22 (m, 4H, ArH), 7.34 (s, 1H, NH), 7.45-7.77 (s, 1H, NH; s, 1H, CH; s, 1H, CH), 9.43 (s, 1H, OH), 12.97 (s, 1H, NH); The ^{13}C NMR, δ_C, (150MHz, DMSO-*d6*): 170.7, 169.8, 157.5, 155.7, 155.4, 148.4, 131.8, 128.2, 115.7, 72.6, 53.0, 51.3. LC/MS (ESI): 343 $[M+H]^+$.

7-(1H-1,2,4-Ttriazol-5-ylamino)-5-(3-hydroxyphenyl)-4,5,6,7-tetrahydro-[1,2,4]triazolo[1,5-a]pyrimidine-7-carboxylic acid (**4t**). White solid (34%); [Found: C, 47.15; H, 4.37; N, 31.81. $C_{14}H_{14}N_8O_3{\cdot}H_2O$ requires C, 46.67; H, 4.48; N, 31.10 %]; The ^{1}H NMR, δ_H, (300 MHz, DMSO-*d6*): 2.99-3.24, 2.14-2.39 (m, 2H, CH_2), 4.67-4.78 (m, 1H, CH), 6.65-7.19 (m, 4H, ArH), 7.31 (s, 1H, NH), 7.48-7.81 (s, 1H, NH; s, 1H, CH; s, 1H, CH), 9.45 (s, 1H, OH); LC/MS (ESI): 343 $[M+H]^+$.

7-(1H-1,2,4-Triazol-5-ylamino)-5-(2-hydroxyphenyl)-4,5,6,7-tetrahydro-[1,2,4]triazolo[1,5-a]pyrimidine-7-carboxylic acid (**4u**). White solid (49%); [Found: C, 45.85; H, 4.76; N, 29.89. $C_{14}H_{14}N_8O_3{\cdot}H_2O$ requires C, 46.67; H, 4.48; N, 31.10 %]; The ^{1}H NMR, δ_H, (300 MHz, DMSO-*d6*): 2.93-3.10, 2.33-2.39 (m, 2H, CH_2), 5.09-5.17 (m, 1H, CH), 6.75-7.41 (m, 4H, ArH), 7.30 (s, 1H, NH), 7.45-7.74 (s, 1H, CH; s, 1H, NH; s, 1H, CH), 9.62 (s, 1H, OH); LC/MS (ESI): 343 $[M+H]^+$.

3-Cyano-7-(4-methoxyphenyl)-4,7-dihydropyrazolo[1,5-a]pyrimidine-5-carboxylic acid (**7**):

Reaction of **1a** and **8**: A mixture of 5-aminopyrazole-4-carbonitrile (**1a**, 0.1 g, 0.95 mmol) and ethyl (*E*)-4-(4-methoxyphenyl)-2-oxobut-3-enoate (**8**, 0.2 g, 0.95 mmol) in 2 mL of acetic acid was heated at reflux for 10 min. After cooling, the precipitate formed was filtered off and dried in vacuum.

Reaction of **1a**, **2b**, and **3a**: A mixture of 5-aminopyrazole-4-carbonitrile (**1a**, 0.1 g, 0.95 mmol), 4-methoxybenzaldehyde (**2b**, 0.13 g, 0.95 mmol), and pyruvic acid (**3a**, 0.08 g, 0.95 mmol) was refluxed in 2 mL of acetic acid for 4 h. The mixture was allowed to stand until a precipitate formed. The precipitate formed was filtered off and dried in vacuum.

Synthesis of **7** from tetrahydro derivative **4b**: Compound **4b** (40 mg) was refluxed for 2 h in 2 mL of acetic acid. After cooling, the precipitate was filtered off and dried in vacuum.

Yellow solid (0.08 g, 28% - from reaction of **1a**+**8**; 0.18 g, 66% - from reaction of **1a**, **2b**, and **3a**; 0.01 g, 35% - from tetrahydro derivative **4b**), m.p. 247-249 °C. [Found: C, 60.77; H, 4.15; N, 18.98. $C_{15}H_{12}N_4O_3$ requires C, 60.81; H, 4.08; N, 18.91%]; The ^{1}H NMR, δ_H, (400 MHz, DMSO-*d6*): 3.70 (s, 1H, OCH_3), 5.82 (dd, ^3J 4.3 Hz, ^4J 1.5 Hz, 1H, CH), 6.14 (d, ^3J 4.3 Hz, 1H, CH), 6.84-7.14 (m, 4H, Ar), 7.76 (s, 1H, CH), 10.14 (d, ^4J 1.5 Hz, 1H, NH), 12.42 (br s, 1H, COOH); The ^{13}C NMR, δ_C (100 MHz, DMSO-*d6*): 163.2, 159.7, 150.4, 143.4, 142.7, 133.3, 128.6, 126.9, 114.6, 107.9, 74.1, 59.4, 55.7. ESI MS, m/z (%): negative mode 295 (100) $[M\text{-}H]^-$.

X-ray analysis

The colorless crystals of **4g** ($C_{22}H_{20}N_9O_2Br$) are triclinic. At 293 K *a* = 8.6432(6), *b* = 10.8530(8), *c* = 14.1860(9) Å, α = 96.470(6)°, β = 107.447(6)°, γ = 107.557(7)°, *V* = 1179.78(15) Å^3, M_r = 522.38, Z = 2, space group $P\bar{1}$, d_{calc} = 1.471 g/cm^3, $\mu(\text{Mo } K_\alpha)$ = 1.781 mm^{-1}, F(000) = 532. Intensities of 24215 reflections (4649 independent, R_{int} = 0.078) were measured on the Xcalibur-3 diffractometer (graphite monochromated Mo K_α radiation, CCD detector, ω-scaning, $2\Theta_{max}$ = 52°). The structure was solved by direct method using SHELXTL package [3]. The absorption was taken into account using an analytical approach (T_{min} = 0.740, T_{max} = 0.871). The positions of the hydrogen atoms were located from electron density difference maps and refined by a riding model with $U_{iso} = n \cdot U_{eq}$ (*n* = 1.5 for methyl groups and *n* = 1.2 for other hydrogen atoms) of the carrier atom. Full-matrix least-squares refinement against F^2 in anisotropic approximation for nonhydrogen atoms using 4649 reflections was converged to wR_2 = 0.208 (R_1 = 0.083 for 2628 reflections, with $F > 4\sigma(F)$, S = 0.988). The final atomic coordinates and crystallographic data for molecule **4g** have been deposited with the Cambridge Crystallographic Data Centre, 12 Union Road, CB2 1EZ, UK (fax: +44-1223-336033;

email: deposit@ccdc.cam.ac.uk), and are available on request quoting the deposition numbers CCDC 1970864).

References

1. Srivastava, B. K.; Joharapurkar, A.; Raval, S.; Patel, J. Z.; Soni, R.; Raval, P.; Gite, A.; Jain, M. R.; Patel, P. R. *J. Med. Chem.* **2007**, *50,* 5951 **-** 5966. doi:org/10.1021/jm061490u

2. Singla, P.; Luxami, V.; Singh, R.; Tandon, V.; Paul, K. *Eur. J. Med. Chem.*, **2017**, *126*, 24 – 35. doi:org/10.1016/j.ejmech.2016.09.093

3. Sheldrick, G.M. *Acta Crystallogr., Sect. A* **2008**, *A64*, 112.
