# Supplementary material for: Ultrasonic-assisted unusual four-component synthesis of 7-azolylamino-4,5,6,7-tetrahydroazolo[1,5-a]pyrimidines
Source: Beilstein J Org Chem. 2020 Feb 27;16:281–9. doi: 10.3762/bjoc.16.27 (PMC7059450; doi:10.3762/bjoc.16.27)
Supplement: File 2 — 1H and 13C NMR spectra. [file Beilstein_J_Org_Chem-16-281-s002.pdf]

BEILSTEIN JOURNAL OF ORGANIC CHEMISTRY

Supporting Information

for

Ultrasonic-assisted unusual four-component synthesis of 7-azolylamino-4,5,6,7-tetrahydroazolo[1,5-*a*]pyrimidines

Yana I. Sakhno, Maryna V. Murlykina, Oleksandr I. Zbruyev, Anton V. Kozyryev, Svetlana V. Shishkina, Dmytro Sysoiev, Vladimir I. Musatov, Sergey M. Desenko and Valentyn A. Chebanov

Beilstein J. Org. Chem. **2020,** *16,* 281–289. doi:10.3762/bjoc.16.27

^{1}H and ^{13}C NMR spectra

License and Terms: This is a supporting information file under the terms of the Creative Commons Attribution License (https://creativecommons.org/licenses/by/4.0). Please note that the reuse, redistribution and reproduction in particular requires that the authors and source are credited.
The license is subject to the *Beilstein Journal of Organic Chemistry* terms and conditions: (https://www.beilstein-journals.org/bjoc)

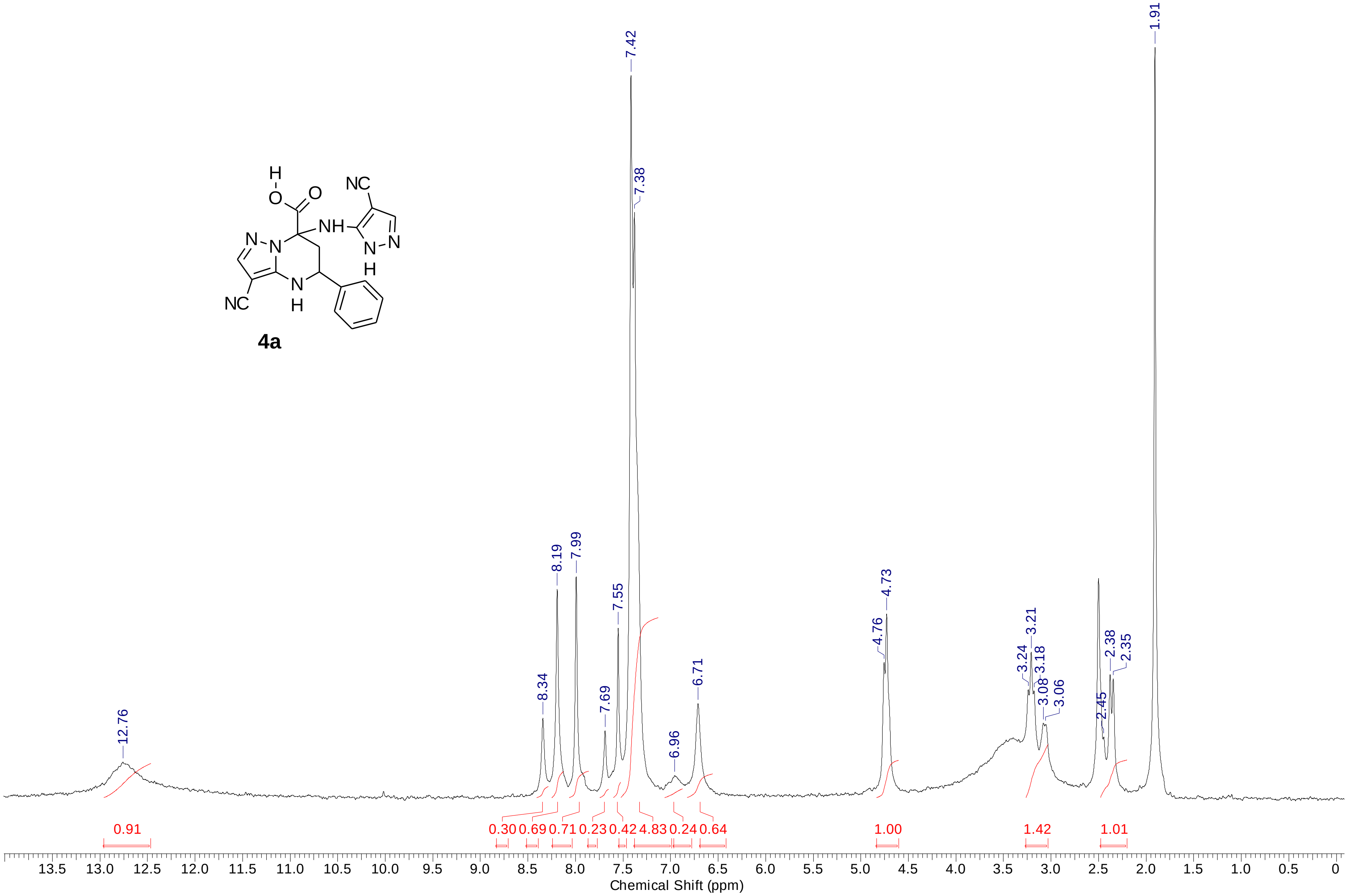
4a
12.76
8.34
8.19
7.99
7.69
7.55
7.42
7.38
6.96
6.71
4.76
4.73
3.24
3.21
3.18
3.08
3.06
2.45
2.38
2.35
1.91
0.91
0.30
0.69
0.71
0.23
0.42
4.83
0.24
0.64
1.00
1.42
1.01
13.5 13.0 12.5 12.0 11.5 11.0 10.5 10.0 9.5 9.0 8.5 8.0 7.5 7.0 6.5 6.0 5.5 5.0 4.5 4.0 3.5 3.0 2.5 2.0 1.5 1.0 0.5 0
Chemical Shift (ppm)

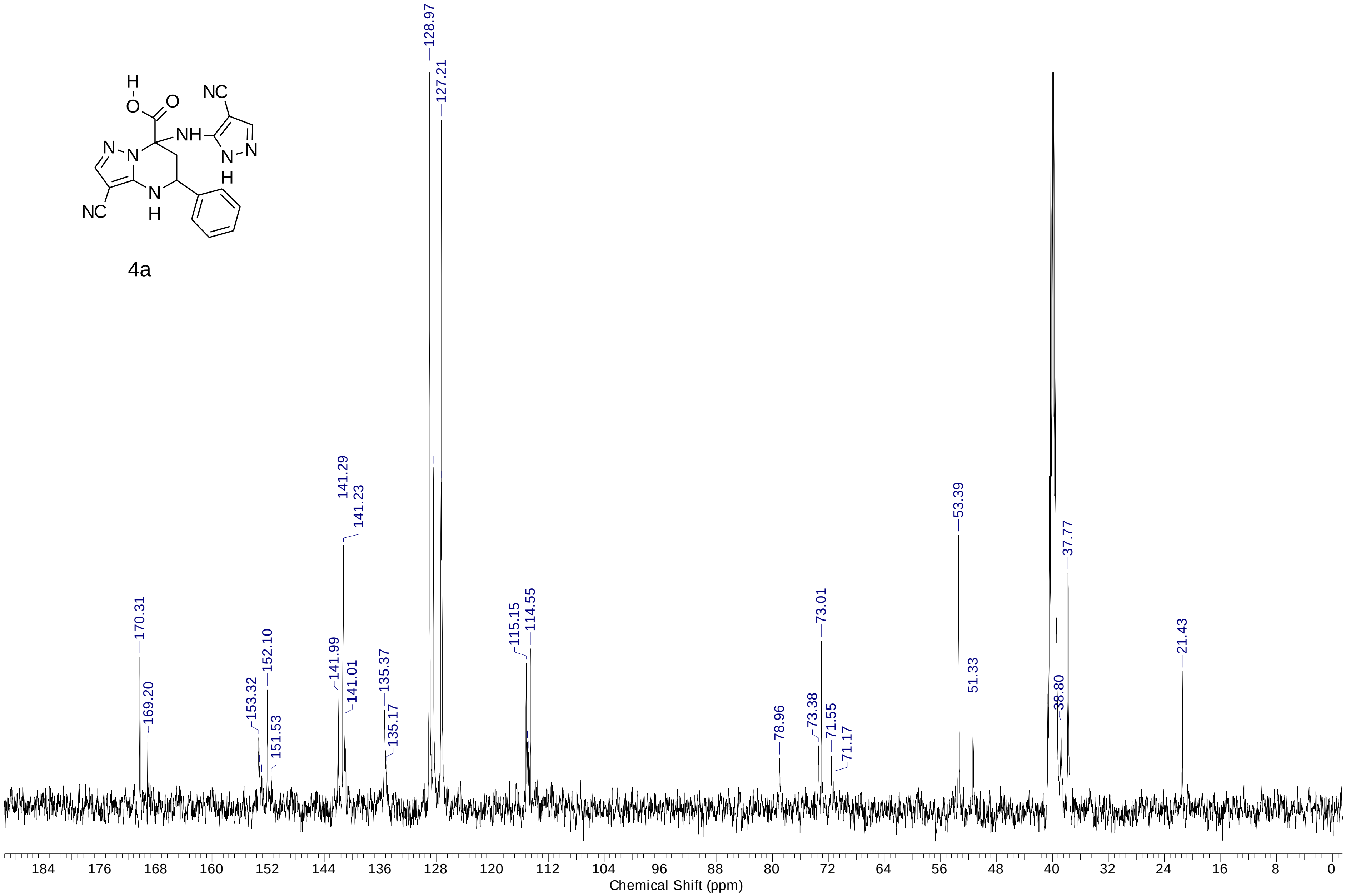
H
O
O
NC
N
N
NH
N
N
H
NC
N
H
4a
128.97
127.21
141.29
141.23
53.39
37.77
170.31
169.20
153.32
152.10
151.53
141.99
141.01
135.37
135.17
115.15
114.55
78.96
73.38
73.01
71.55
71.17
51.33
38.80
21.43
184 176 168 160 152 144 136 128 120 112 104 96 88 80 72 64 56 48 40 32 24 16 8 0
Chemical Shift (ppm)

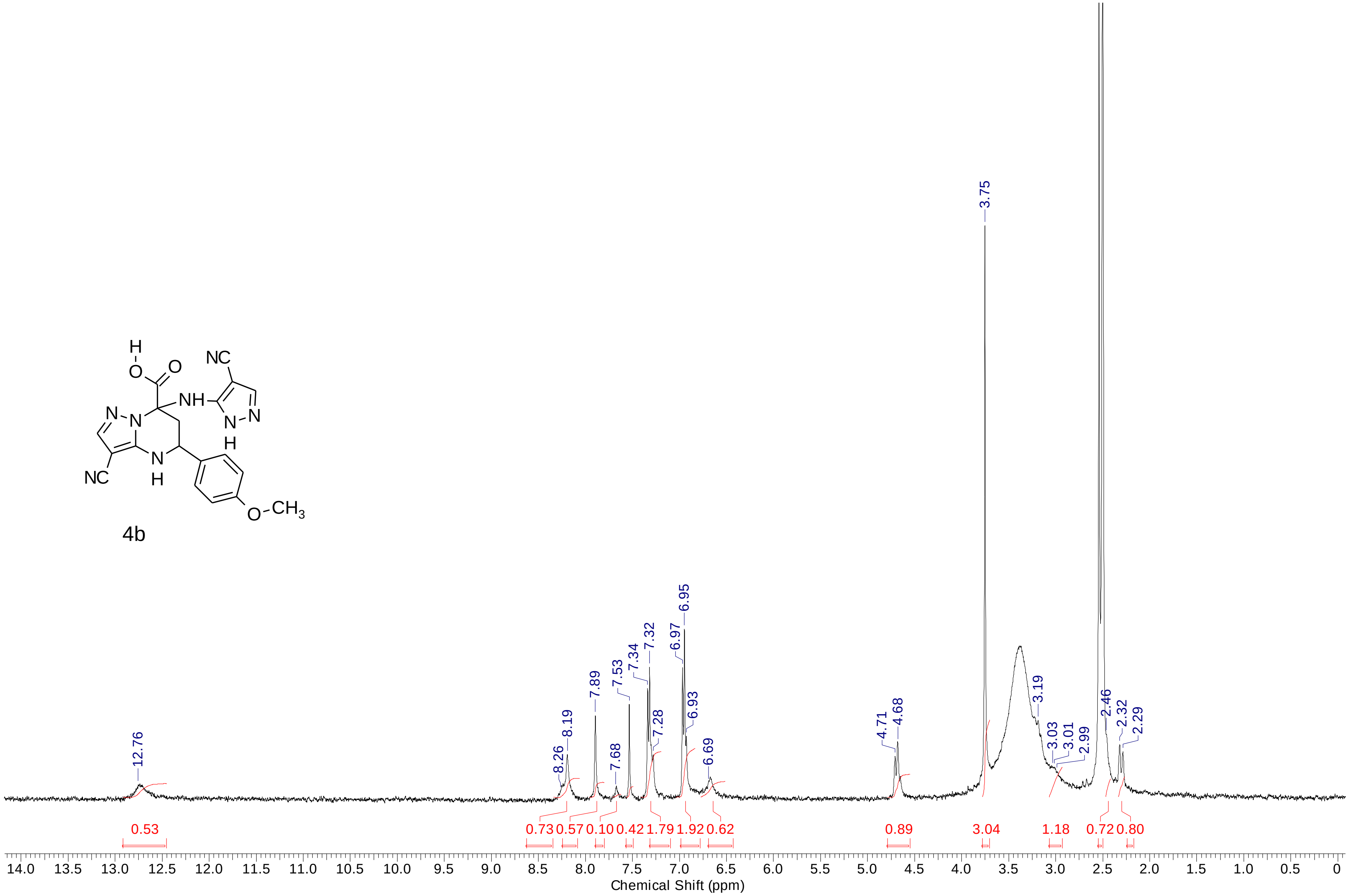
4b
NC
H
O
O
NH
N
N
N
N
H
N
H
NC
O
CH_3
12.76
8.26
8.19
7.89
7.68
7.53
7.34
7.32
7.28
6.97
6.95
6.93
6.69
4.71
4.68
3.75
3.19
3.03
3.01
2.99
2.46
2.32
2.29
0.53
0.73
0.57
0.10
0.42
1.79
1.92
0.62
0.89
3.04
1.18
0.72
0.80
14.0 13.5 13.0 12.5 12.0 11.5 11.0 10.5 10.0 9.5 9.0 8.5 8.0 7.5 7.0 6.5 6.0 5.5 5.0 4.5 4.0 3.5 3.0 2.5 2.0 1.5 1.0 0.5 0
Chemical Shift (ppm)

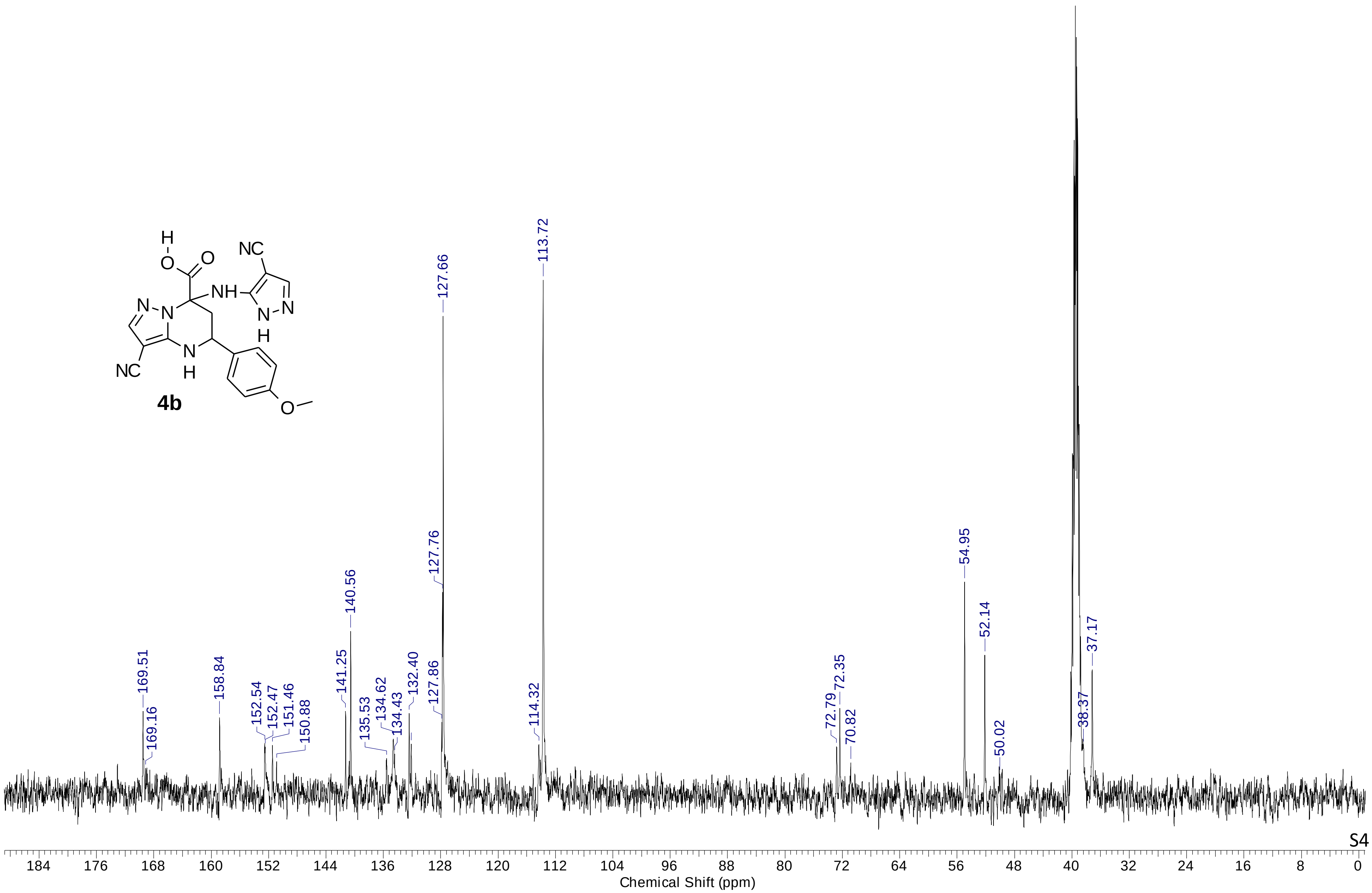
4b
NC
NH
N
H
O
169.51
169.16
158.84
152.54
152.47
151.46
150.88
141.25
140.56
135.53
134.62
134.43
132.40
127.86
127.76
127.66
114.32
113.72
72.79
72.35
70.82
54.95
52.14
50.02
38.37
37.17
184
176
168
160
152
144
136
128
120
112
104
96
88
80
72
64
56
48
40
32
24
16
8
0
Chemical Shift (ppm)

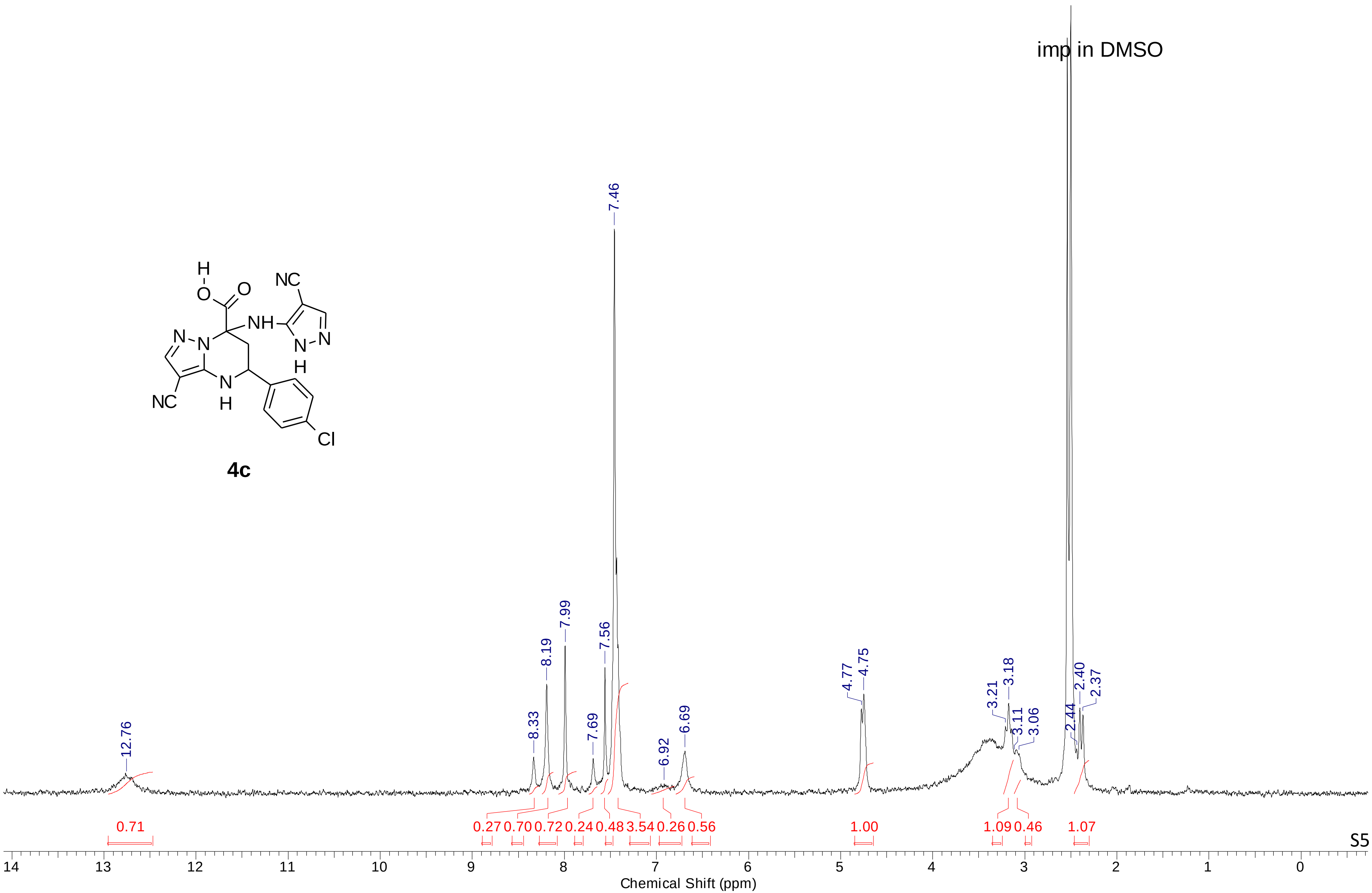
4c
imp in DMSO
12.76
8.33
8.19
7.99
7.69
7.56
7.46
6.92
6.69
4.77
4.75
3.21
3.18
3.11
3.06
2.44
2.40
2.37
0.71
0.27 0.70 0.72 0.24 0.48 3.54 0.26 0.56
1.00
1.09 0.46
1.07
14 13 12 11 10 9 8 7 6 5 4 3 2 1 0
Chemical Shift (ppm)

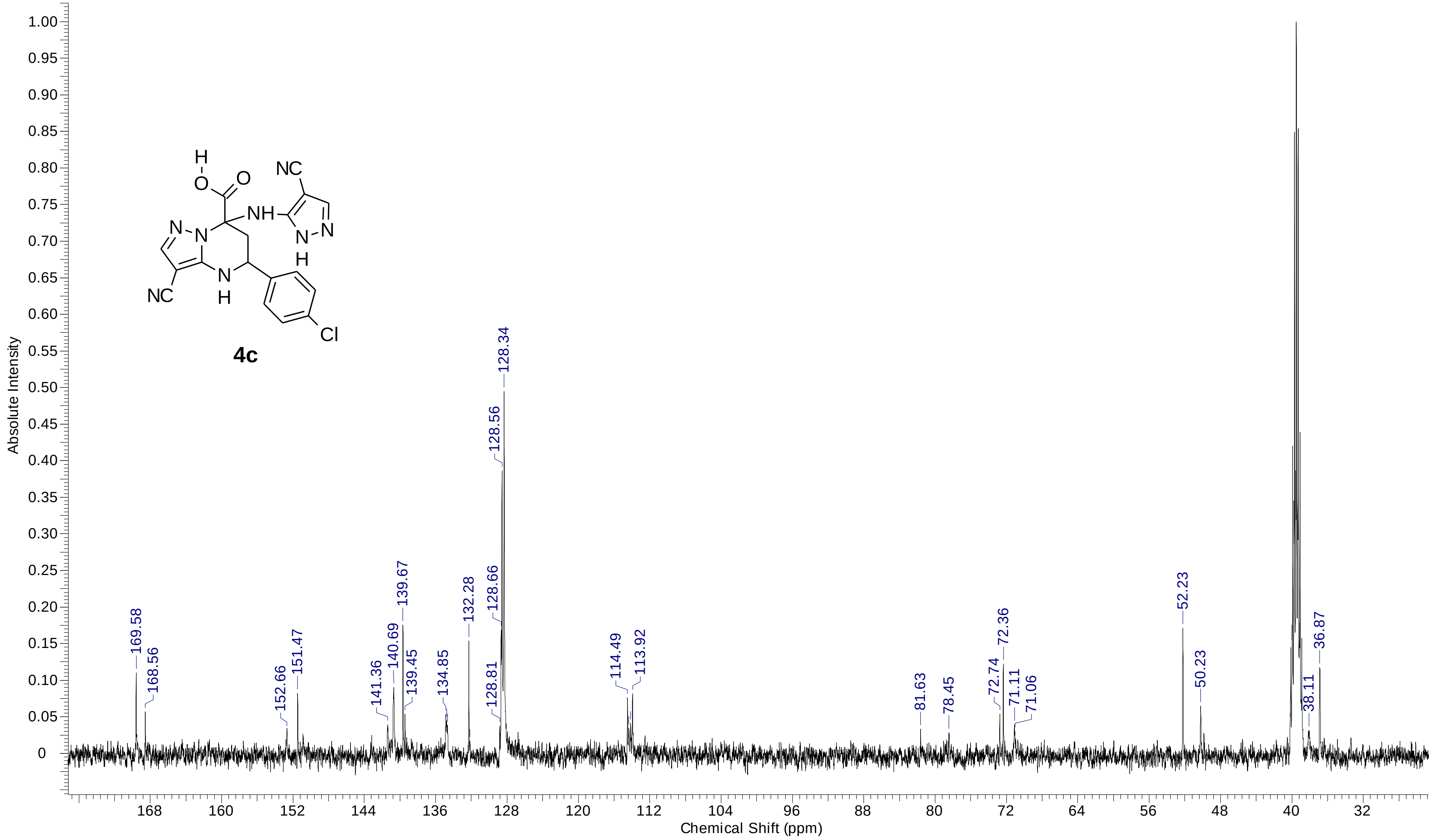
4c
NC
NH
N
H
O
Cl
169.58
168.56
152.66
151.47
141.36
140.69
139.67
139.45
134.85
132.28
128.81
128.66
128.56
128.34
114.49
113.92
81.63
78.45
72.74
72.36
71.11
71.06
52.23
50.23
38.11
36.87
Absolute Intensity
Chemical Shift (ppm)

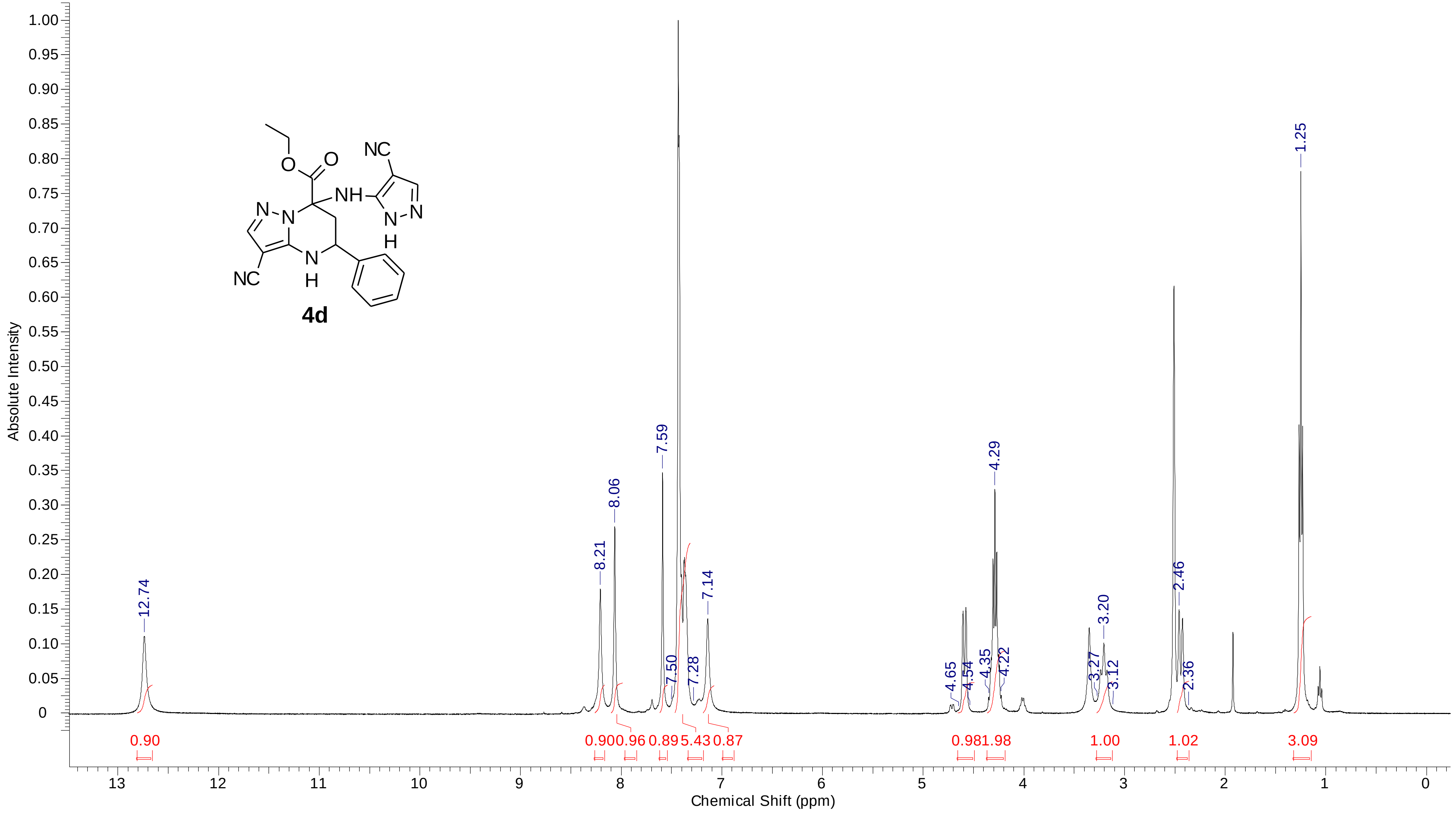
4d
NC
O
O
NH
N
N
N
N
H
NC
N
H
Absolute Intensity
Chemical Shift (ppm)
12.74
8.21
8.06
7.59
7.50
7.28
7.14
4.65
4.54
4.35
4.29
4.22
3.27
3.20
3.12
2.46
2.36
1.25
0.90
0.90
0.96
0.89
5.43
0.87
0.98
1.98
1.00
1.02
3.09

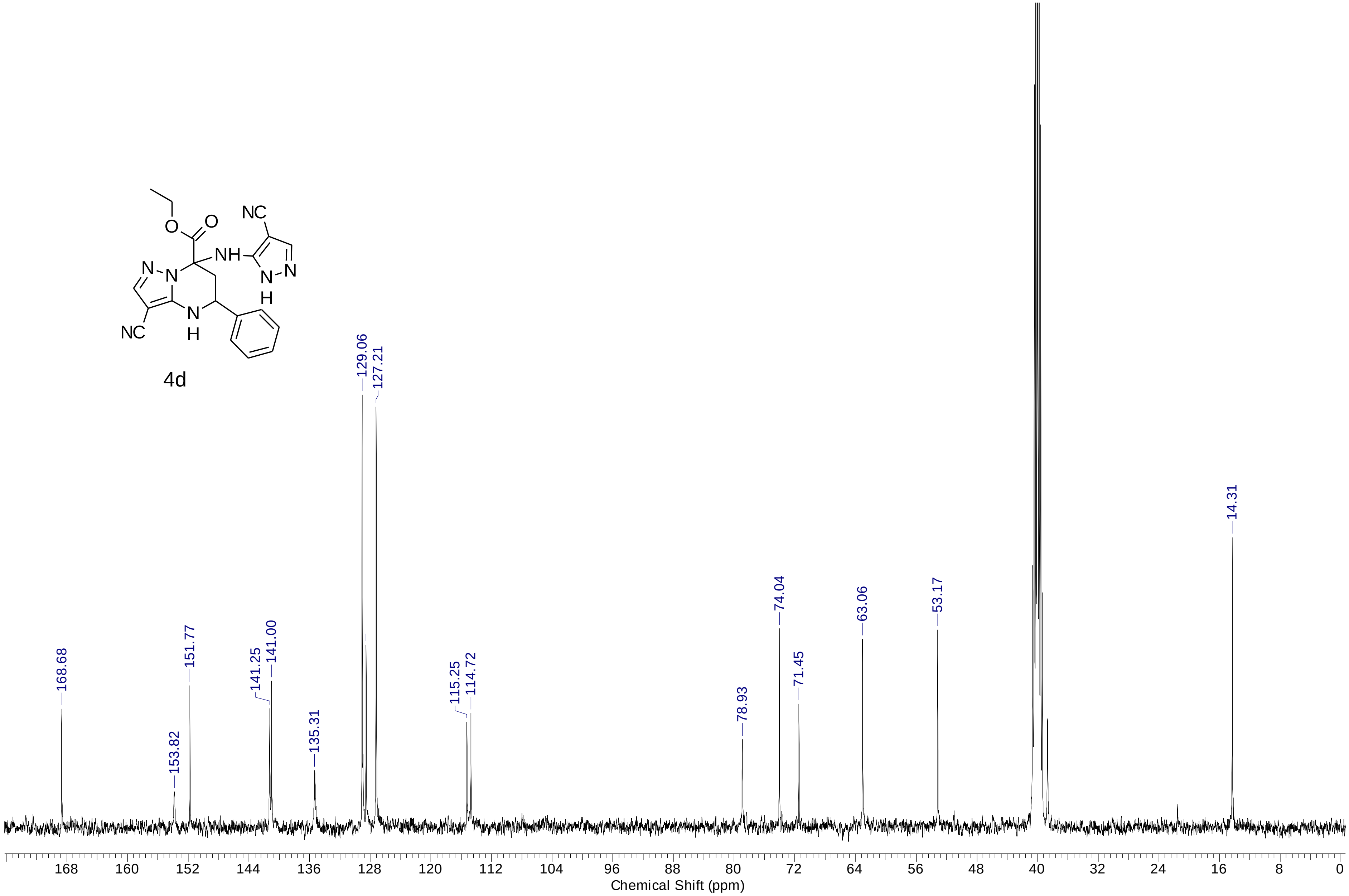
NC
O
O
NH
N
N
N
H
NC
N
H
4d
168.68
153.82
151.77
141.25
141.00
135.31
129.06
127.21
115.25
114.72
78.93
74.04
71.45
63.06
53.17
14.31
168
160
152
144
136
128
120
112
104
96
88
80
72
64
56
48
40
32
24
16
8
0
Chemical Shift (ppm)

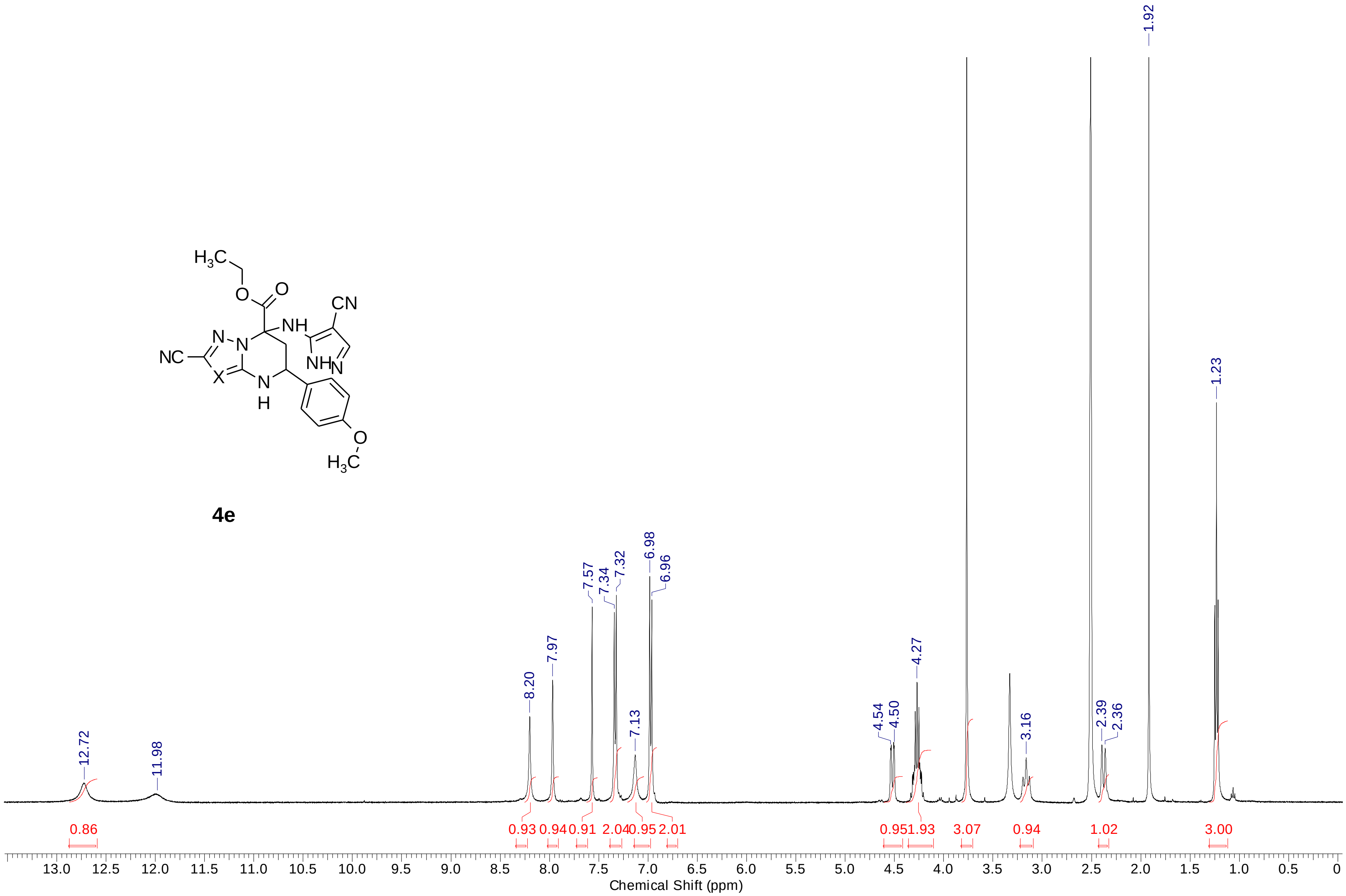
H3C
O
O
NH
CN
N
N
NC
X
N
H
NH
N
O
H3C
12.72
11.98
8.20
7.97
7.57
7.34
7.32
7.13
6.98
6.96
4.54
4.50
4.27
3.16
2.39
2.36
1.92
1.23
0.86
0.93
0.94
0.91
2.04
0.95
2.01
0.95
1.93
3.07
0.94
1.02
3.00
13.0 12.5 12.0 11.5 11.0 10.5 10.0 9.5 9.0 8.5 8.0 7.5 7.0 6.5 6.0 5.5 5.0 4.5 4.0 3.5 3.0 2.5 2.0 1.5 1.0 0.5 0
Chemical Shift (ppm)

4e

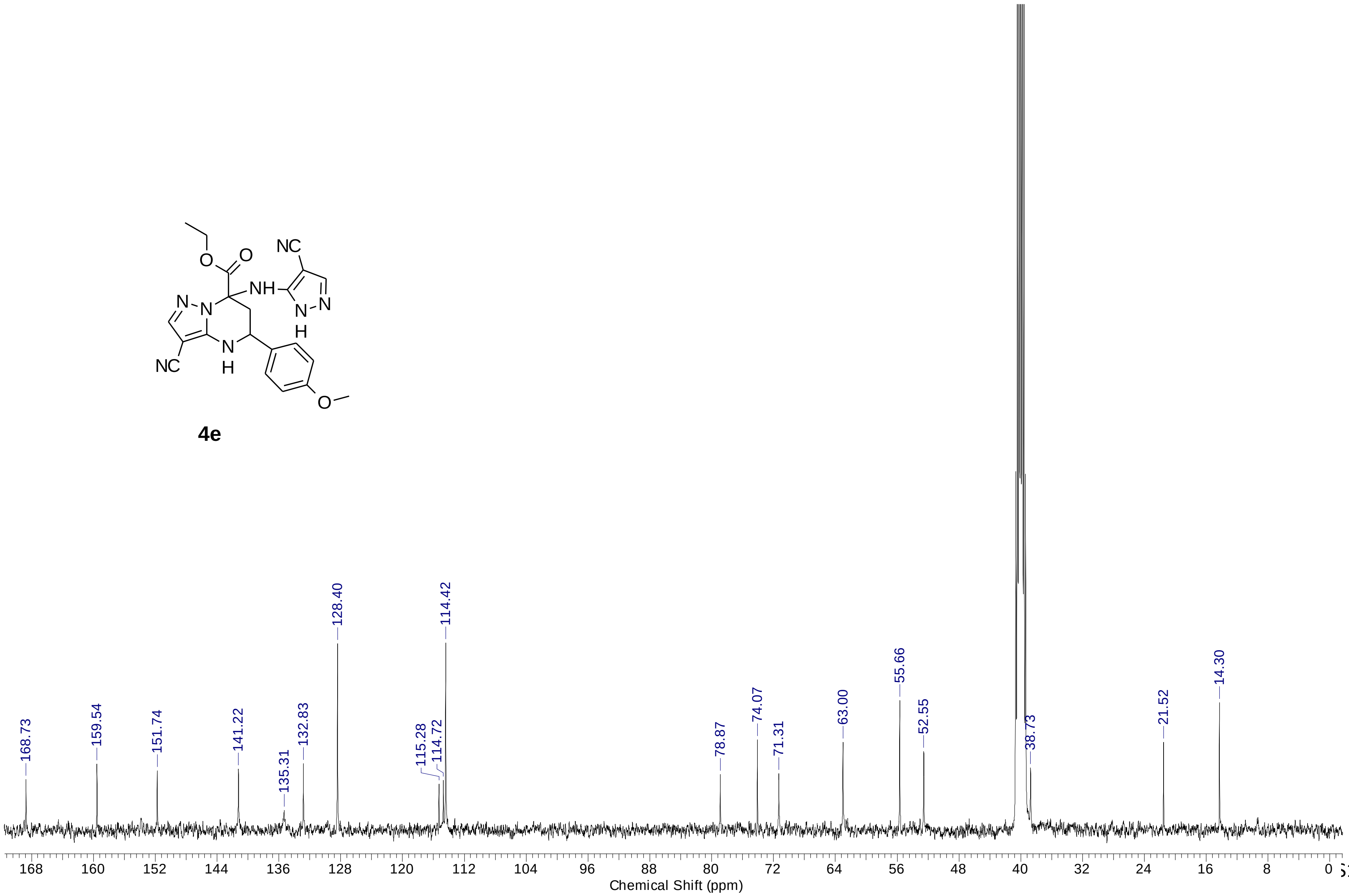

NC
O
O
NH
N
N
N
H
NC
N
N
H
O
4e
168.73
159.54
151.74
141.22
135.31
132.83
128.40
115.28
114.72
114.42
78.87
74.07
71.31
63.00
55.66
52.55
38.73
21.52
14.30
168
160
152
144
136
128
120
112
104
96
88
80
72
64
56
48
40
32
24
16
8
0
Chemical Shift (ppm)

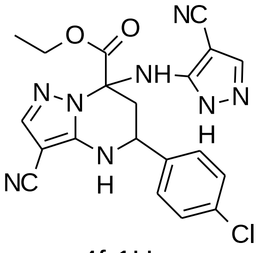
NC
O
O
NH
N
N
N
N
H
NC
N
H
Cl

4f-1H

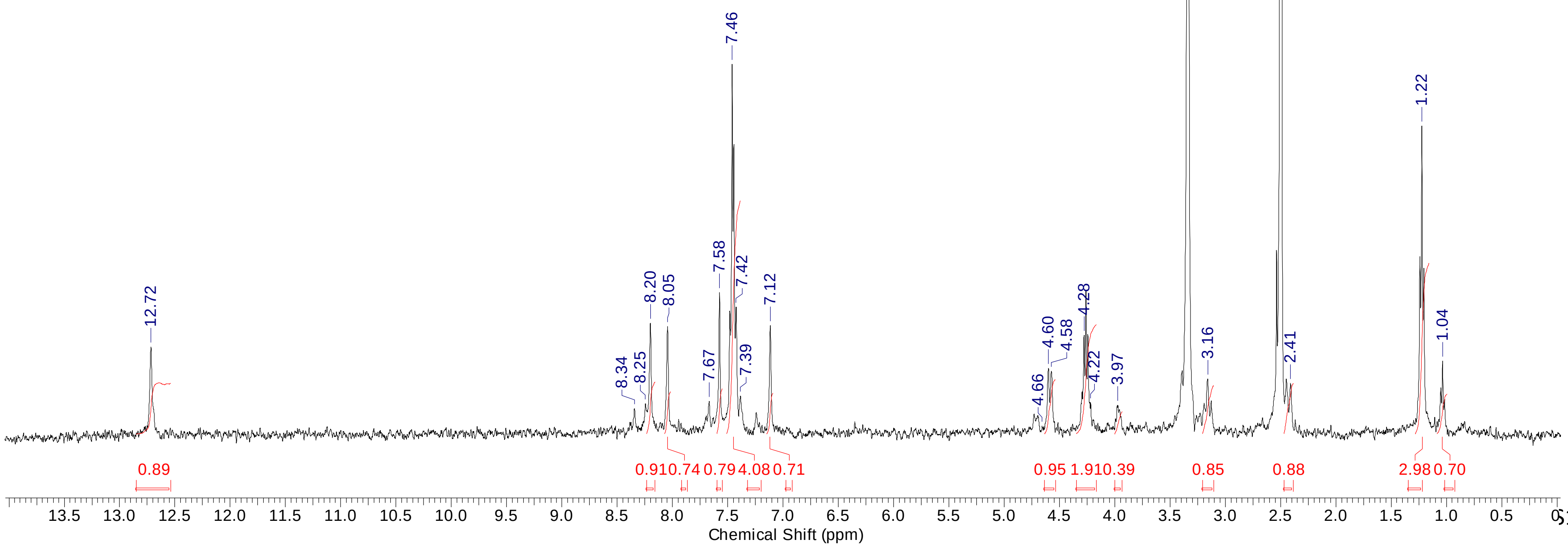
12.72
8.34
8.25
8.20
8.05
7.67
7.58
7.46
7.42
7.39
7.12
4.66
4.60
4.58
4.28
4.22
3.97
3.16
2.41
1.22
1.04
0.89
0.91
0.74
0.79
4.08
0.71
0.95
1.91
0.39
0.85
0.88
2.98
0.70
13.5 13.0 12.5 12.0 11.5 11.0 10.5 10.0 9.5 9.0 8.5 8.0 7.5 7.0 6.5 6.0 5.5 5.0 4.5 4.0 3.5 3.0 2.5 2.0 1.5 1.0 0.5 0
Chemical Shift (ppm)

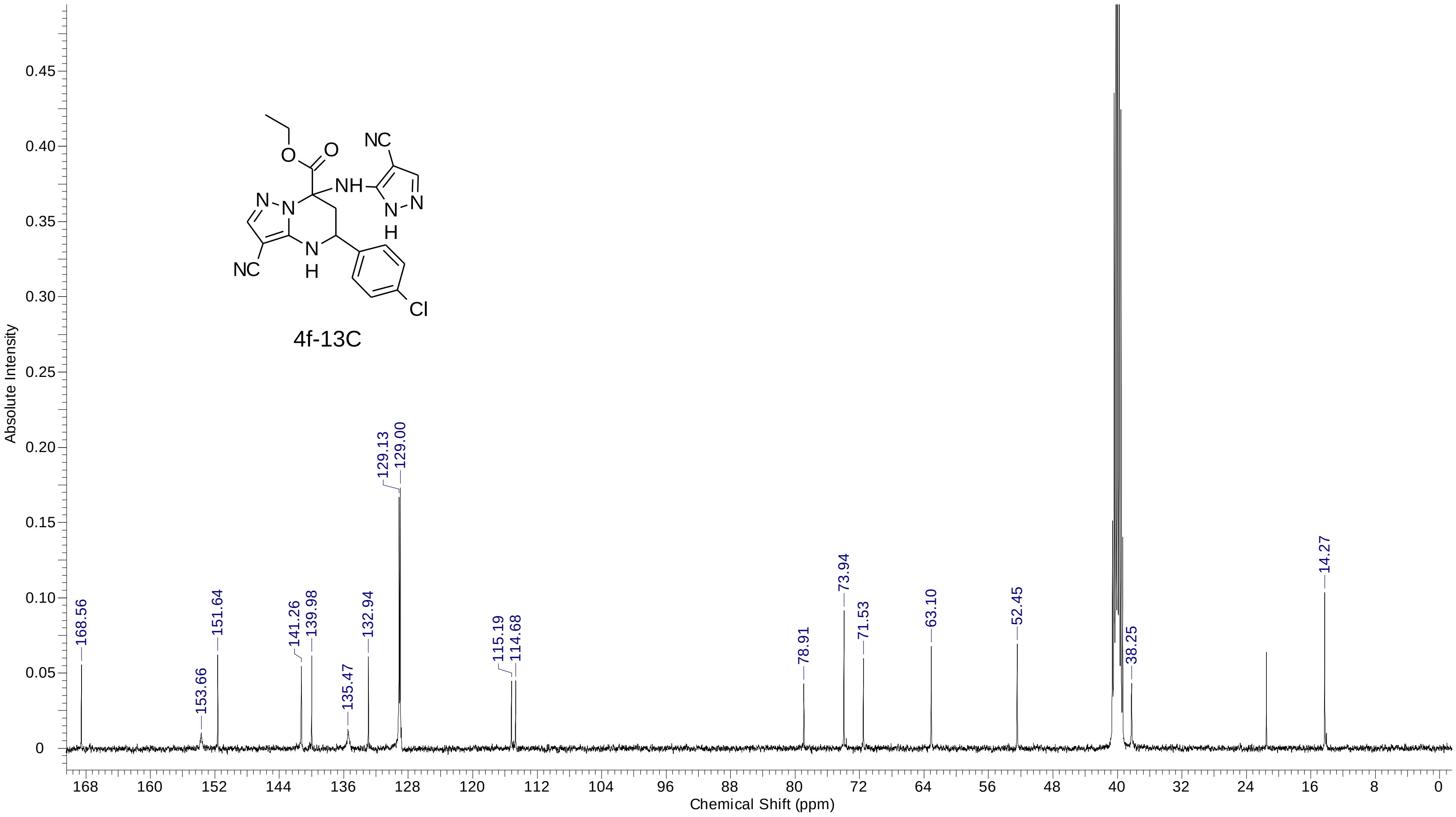
NC
O
O
N
N
NH
N
N
H
NC
N
H
Cl
4f-13C
Absolute Intensity
Chemical Shift (ppm)
168.56
153.66
151.64
141.26
139.98
135.47
132.94
129.13
129.00
115.19
114.68
78.91
73.94
71.53
63.10
52.45
38.25
14.27

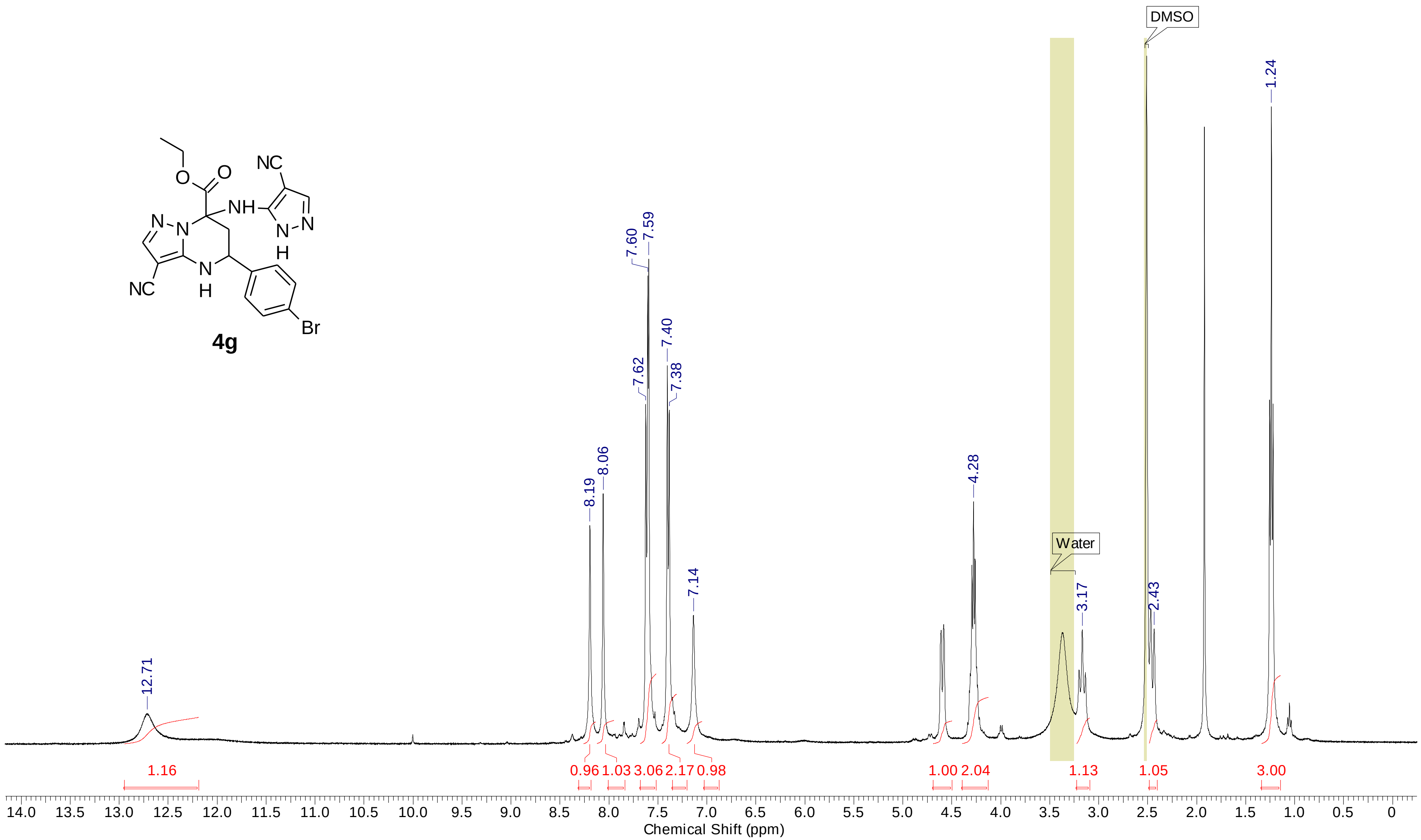
NC
O
O
NH
N
N
N
N
H
N
H
NC
Br
4g
DMSO
Water
12.71
8.19
8.06
7.62
7.60
7.59
7.40
7.38
7.14
4.28
3.17
2.43
1.24
1.16
0.96
1.03
3.06
2.17
0.98
1.00
2.04
1.13
1.05
3.00
14.0
13.5
13.0
12.5
12.0
11.5
11.0
10.5
10.0
9.5
9.0
8.5
8.0
7.5
7.0
6.5
6.0
5.5
5.0
4.5
4.0
3.5
3.0
2.5
2.0
1.5
1.0
0.5
0
Chemical Shift (ppm)

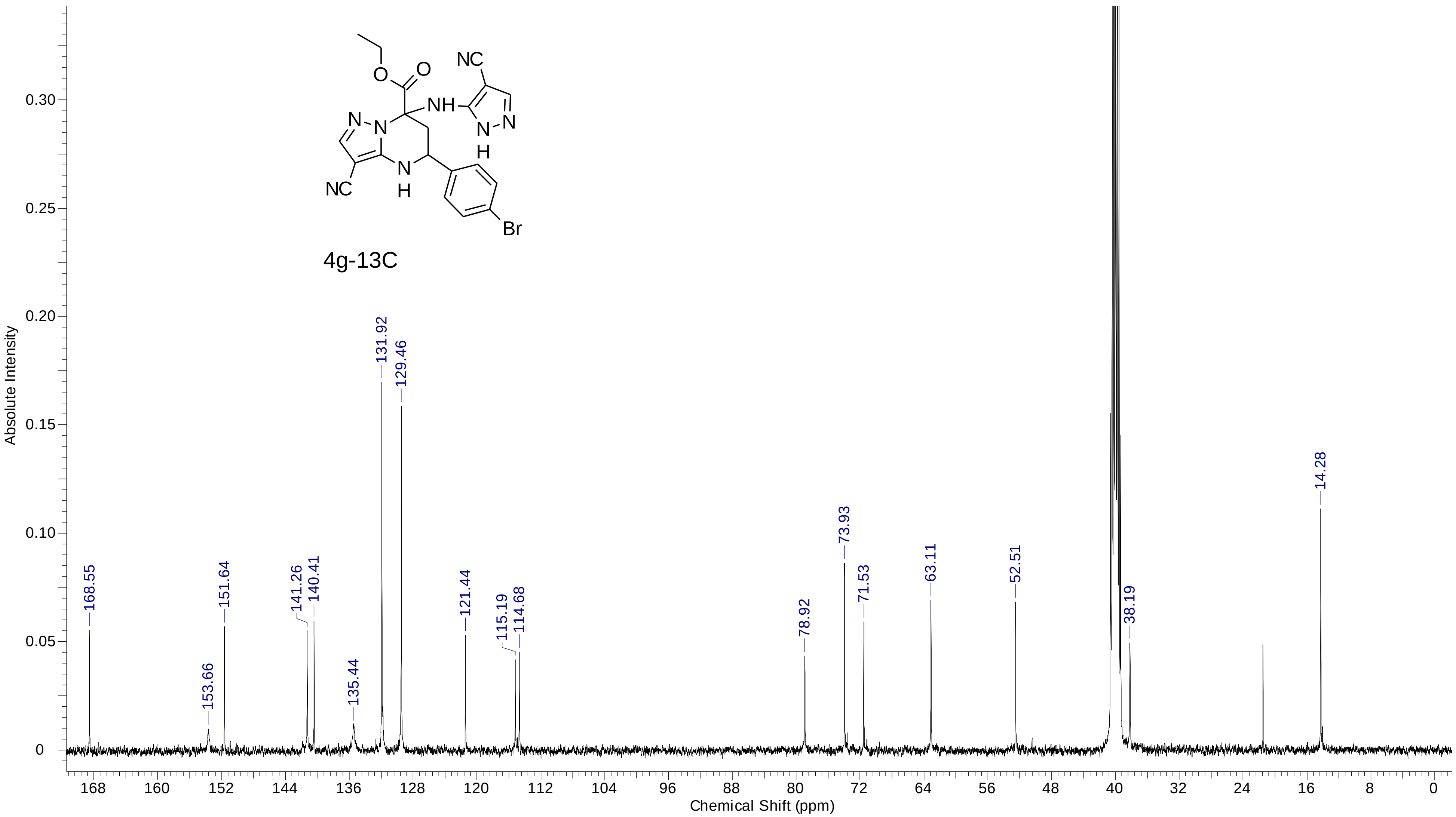

NC
O
O
NH
N
N
N
N
H
NC
N
H
Br
4g-13C
Absolute Intensity
0.30
0.25
0.20
0.15
0.10
0.05
0
168.55
153.66
151.64
141.26
140.41
135.44
131.92
129.46
121.44
115.19
114.68
78.92
73.93
71.53
63.11
52.51
38.19
14.28
168
160
152
144
136
128
120
112
104
96
88
80
72
64
56
48
40
32
24
16
8
0
Chemical Shift (ppm)

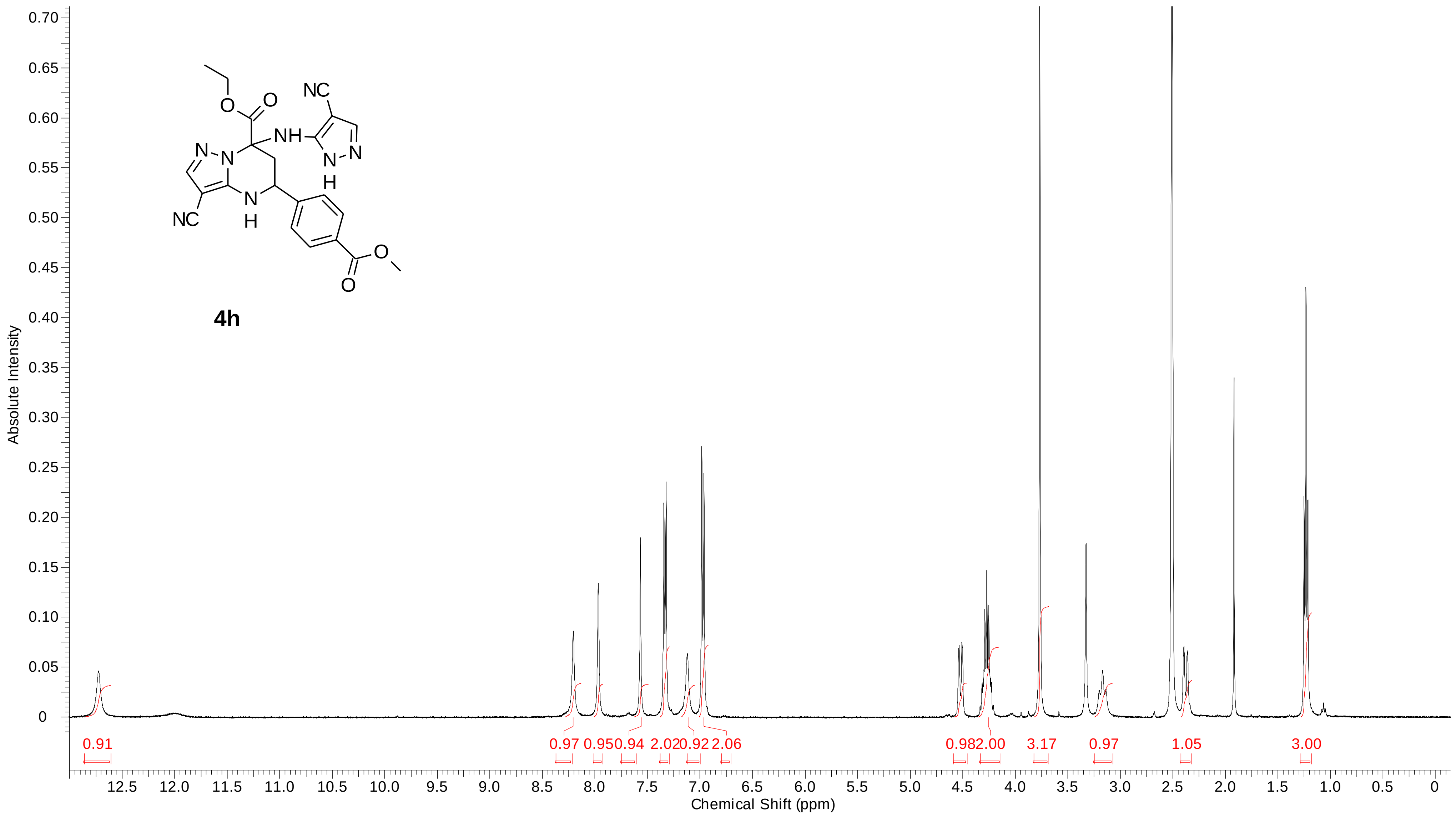
4h
Absolute Intensity
Chemical Shift (ppm)
0.91
0.97
0.95
0.94
2.02
0.92
2.06
0.98
2.00
3.17
0.97
1.05
3.00

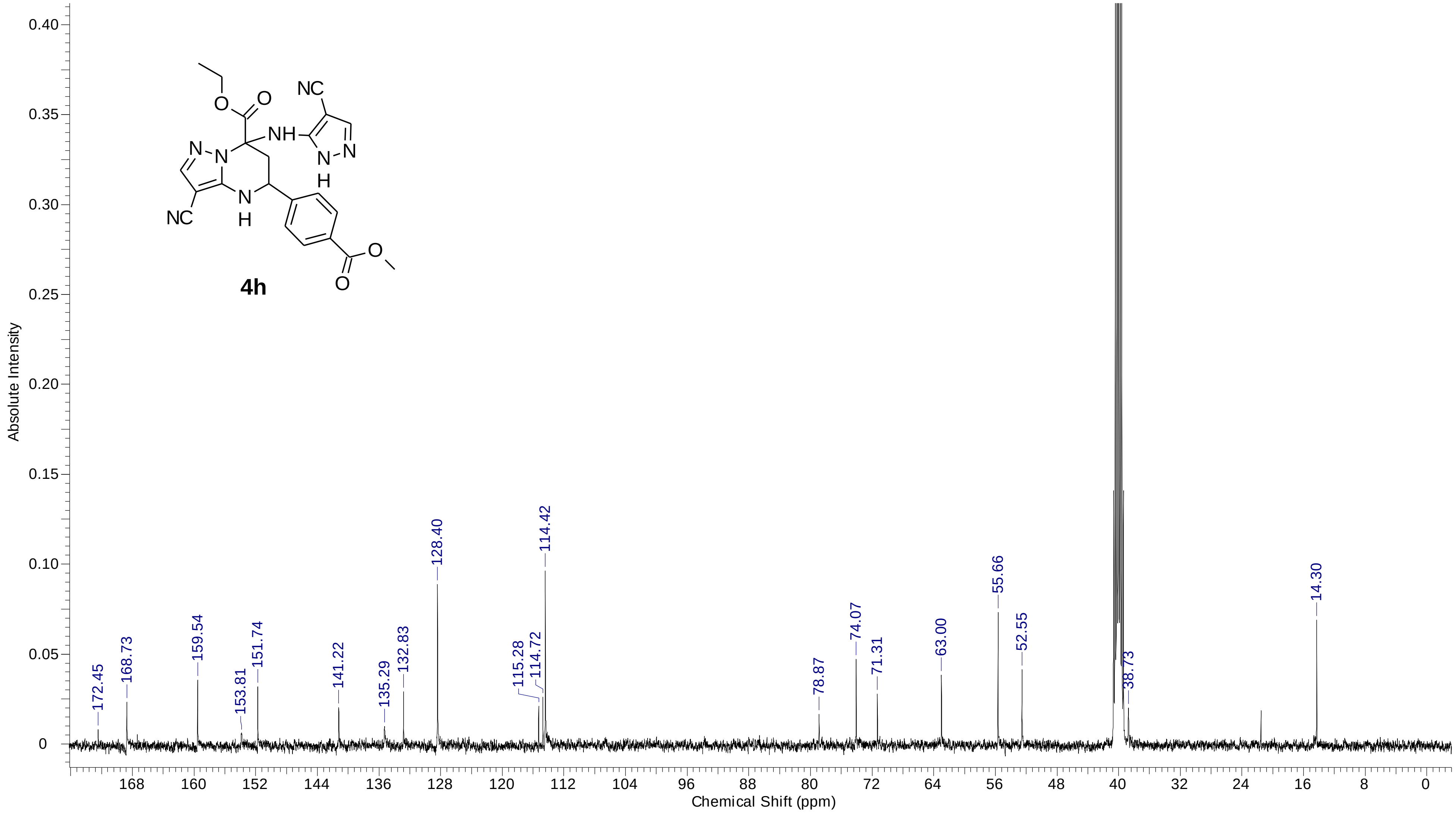
4h
NC
O
O
NH
N
N
N
N
H
NC
N
H
O
O
172.45
168.73
159.54
153.81
151.74
141.22
135.29
132.83
128.40
115.28
114.72
114.42
78.87
74.07
71.31
63.00
55.66
52.55
38.73
14.30
Absolute Intensity
0.40
0.35
0.30
0.25
0.20
0.15
0.10
0.05
0
168
160
152
144
136
128
120
112
104
96
88
80
72
64
56
48
40
32
24
16
8
0
Chemical Shift (ppm)

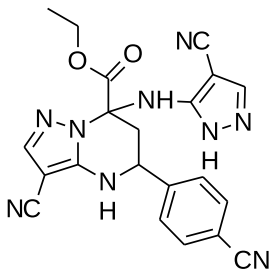

4i

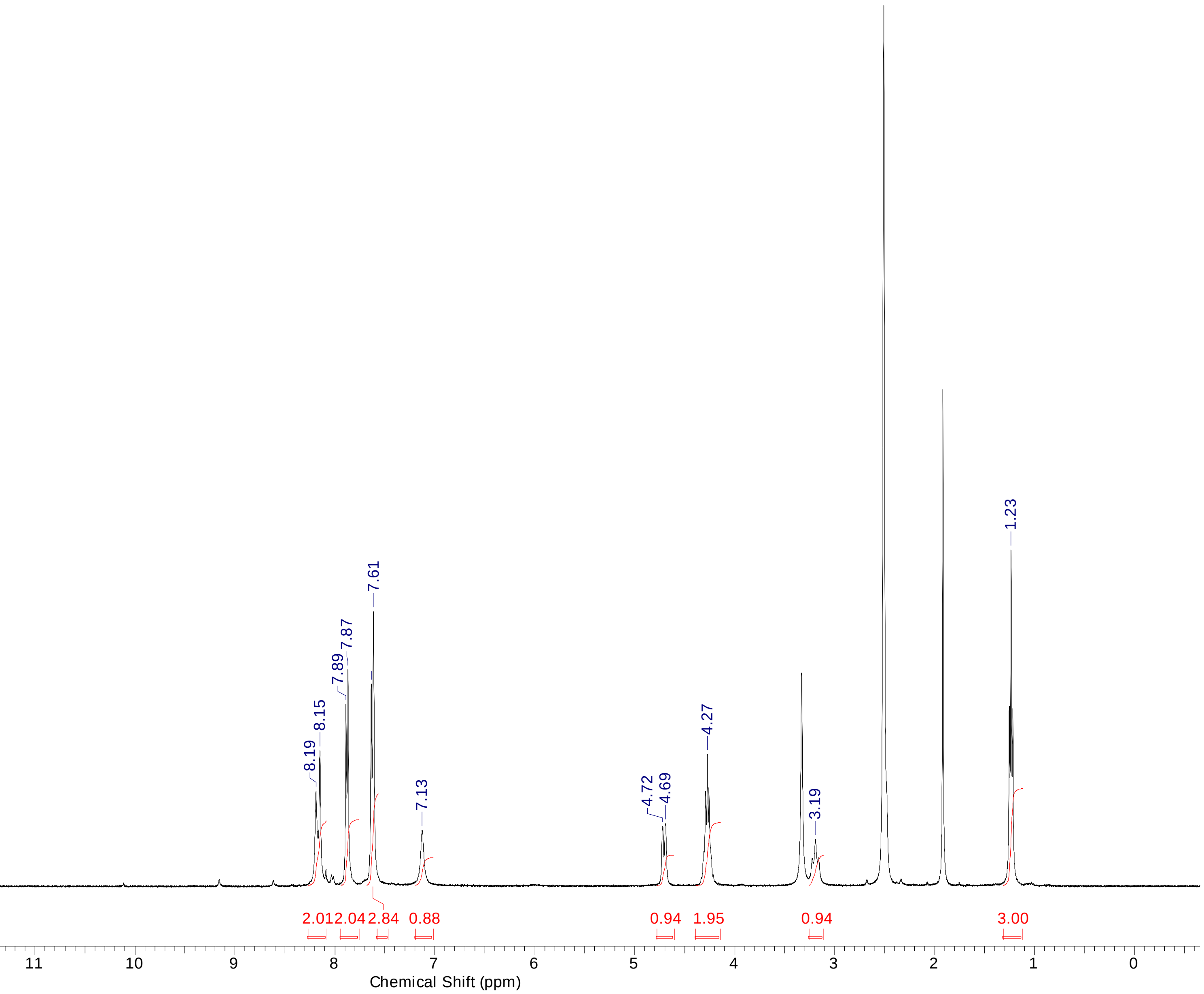

12.71
8.19
8.15
7.89
7.87
7.61
7.13
4.72
4.69
4.27
3.19
1.23
0.79
2.01
2.04
2.84
0.88
0.94
1.95
0.94
3.00
14
13
12
11
10
9
8
7
6
5
4
3
2
1
0
Chemical Shift (ppm)

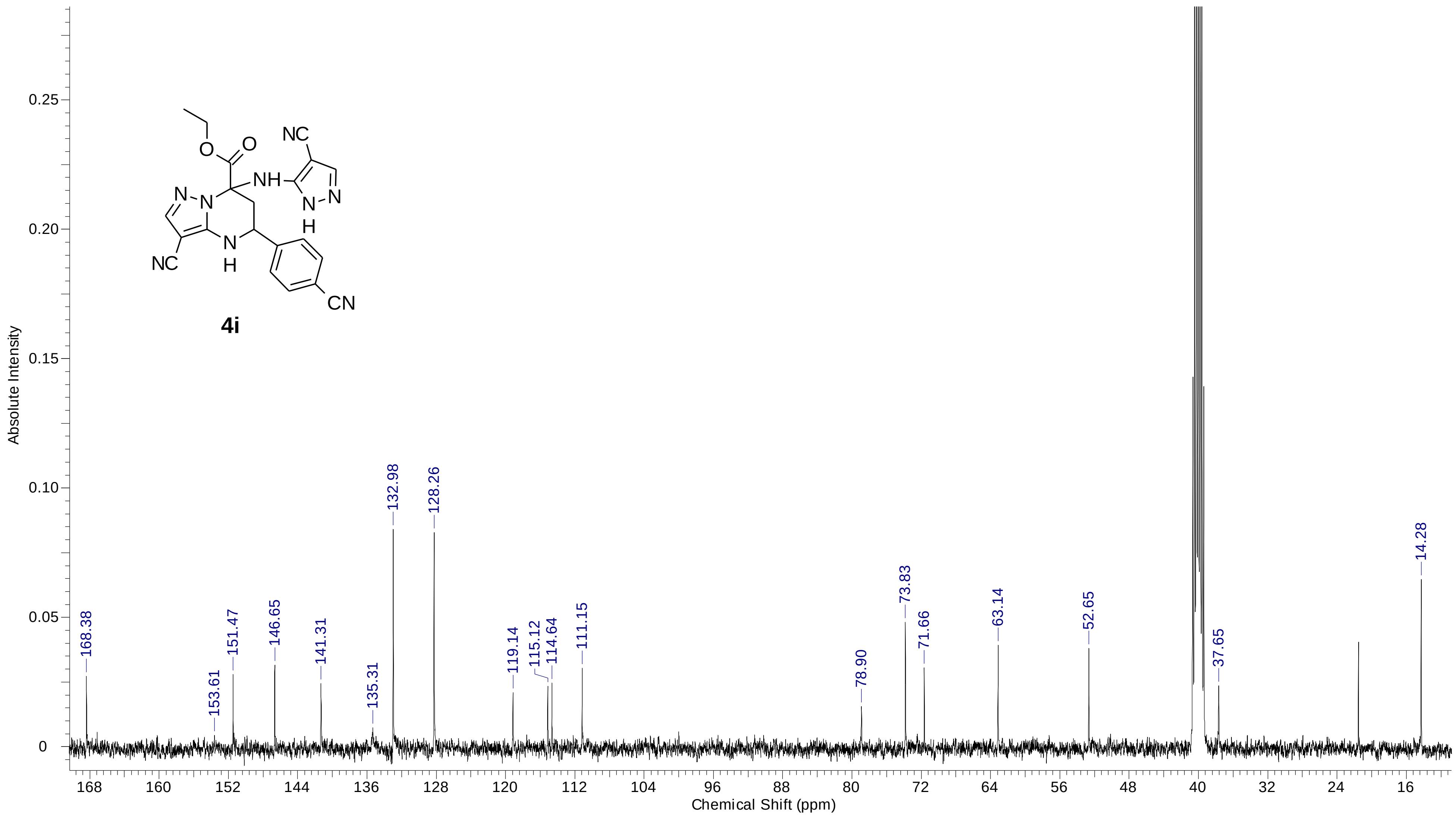

NC
O
O
NH
N
N
N
N
H
NC
N
H
CN
4i
168.38
153.61
151.47
146.65
141.31
135.31
132.98
128.26
119.14
115.12
114.64
111.15
78.90
73.83
71.66
63.14
52.65
37.65
14.28
Absolute Intensity
Chemical Shift (ppm)

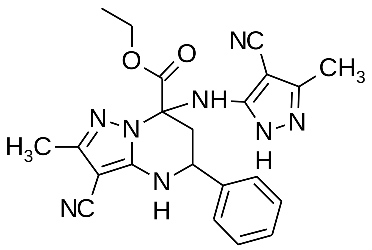
O
O
NC
CH3
NH
N
N
N
N
N
H
H3C
NC
N
H

4j

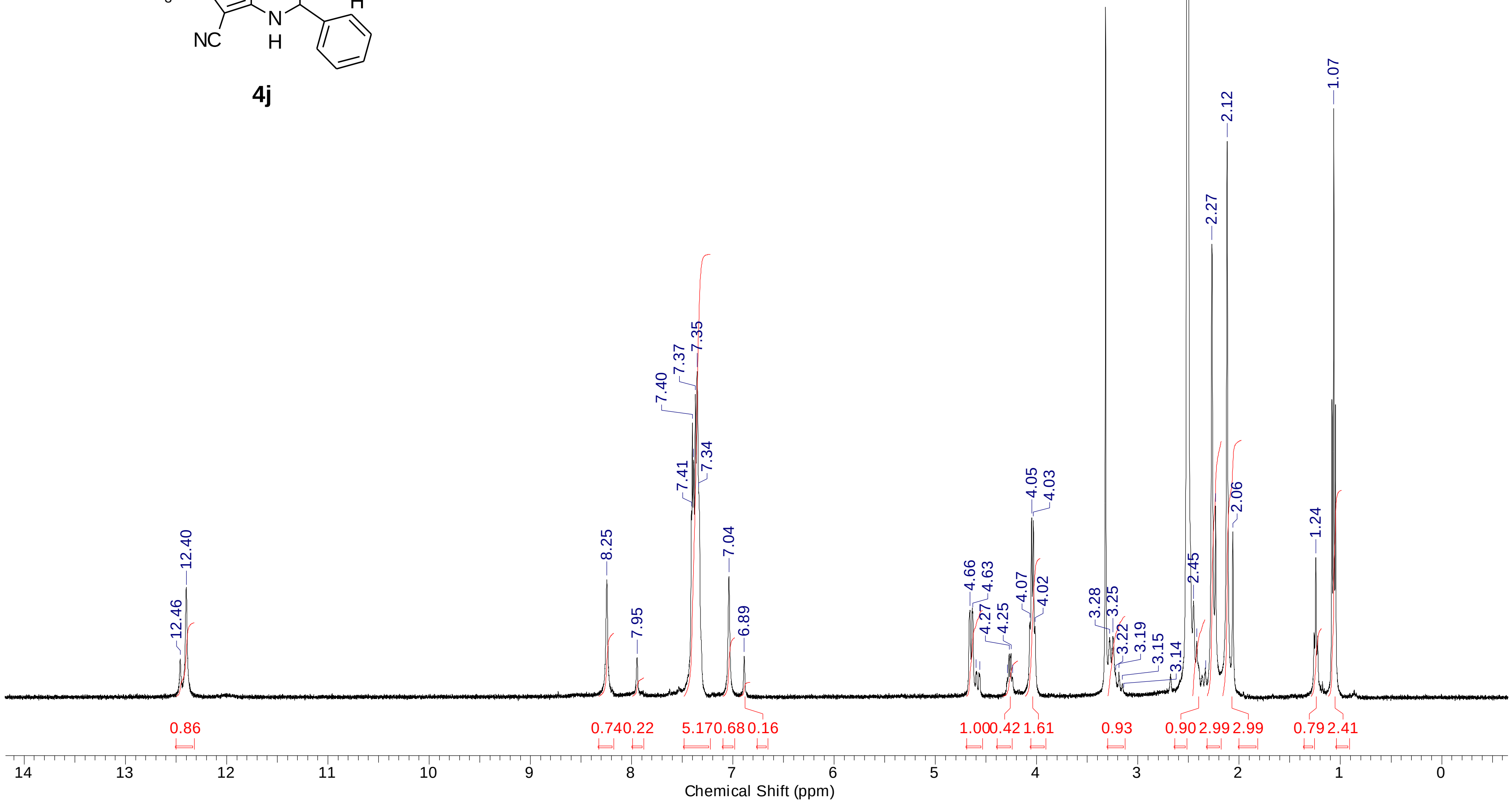
12.46
12.40
8.25
7.95
7.41
7.40
7.37
7.35
7.34
7.04
6.89
4.66
4.63
4.27
4.25
4.07
4.05
4.03
4.02
3.28
3.25
3.22
3.19
3.15
3.14
2.45
2.27
2.12
2.06
1.24
1.07
0.86
0.74
0.22
5.17
0.68
0.16
1.00
0.42
1.61
0.93
0.90
2.99
2.99
0.79
2.41
14
13
12
11
10
9
8
7
6
5
4
3
2
1
0
Chemical Shift (ppm)

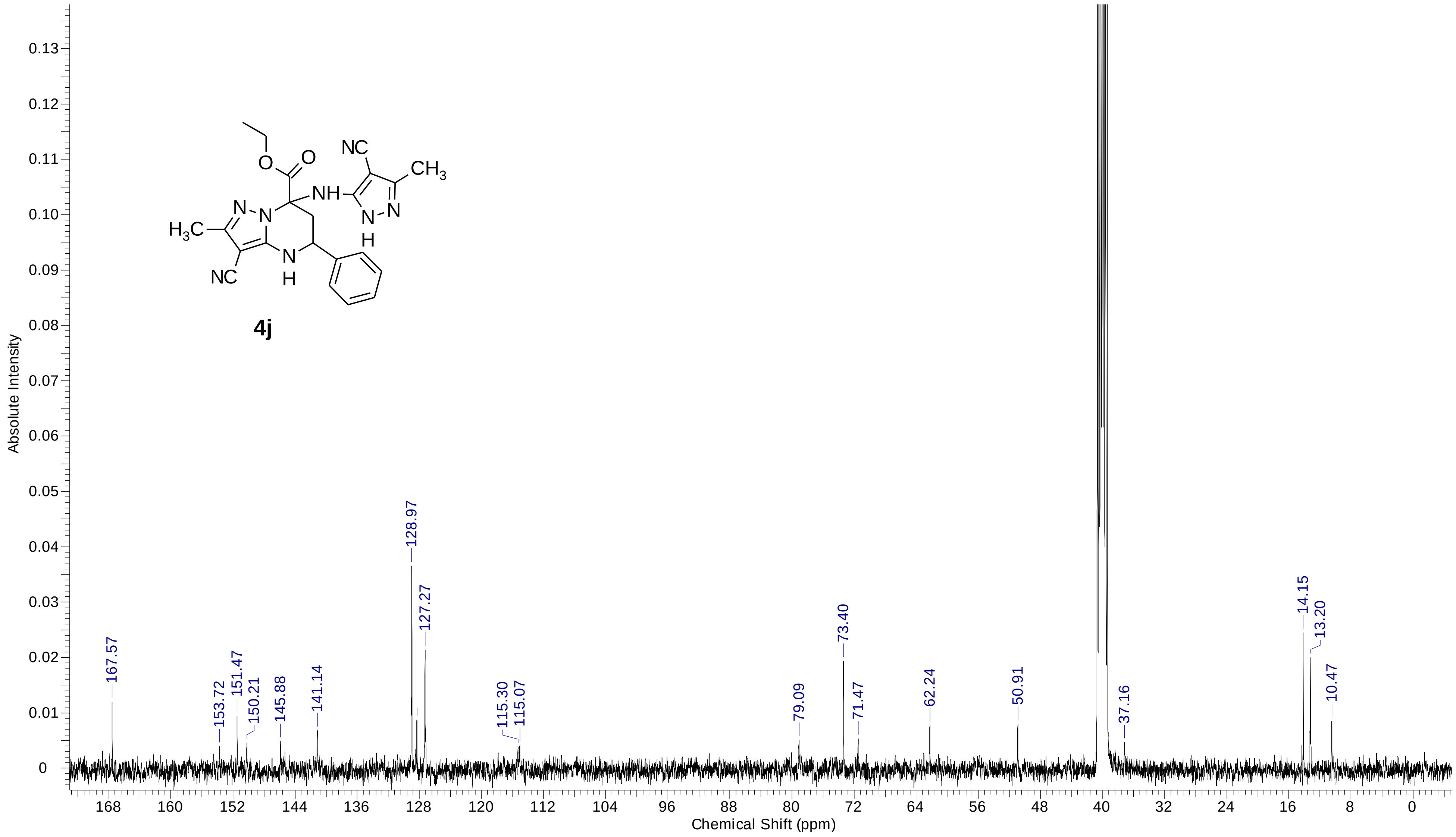

4j
NC
CH3
O
O
NH
N
N
H3C
N
N
H
NC
N
H
167.57
153.72
151.47
150.21
145.88
141.14
128.97
127.27
115.30
115.07
79.09
73.40
71.47
62.24
50.91
37.16
14.15
13.20
10.47
Absolute Intensity
Chemical Shift (ppm)

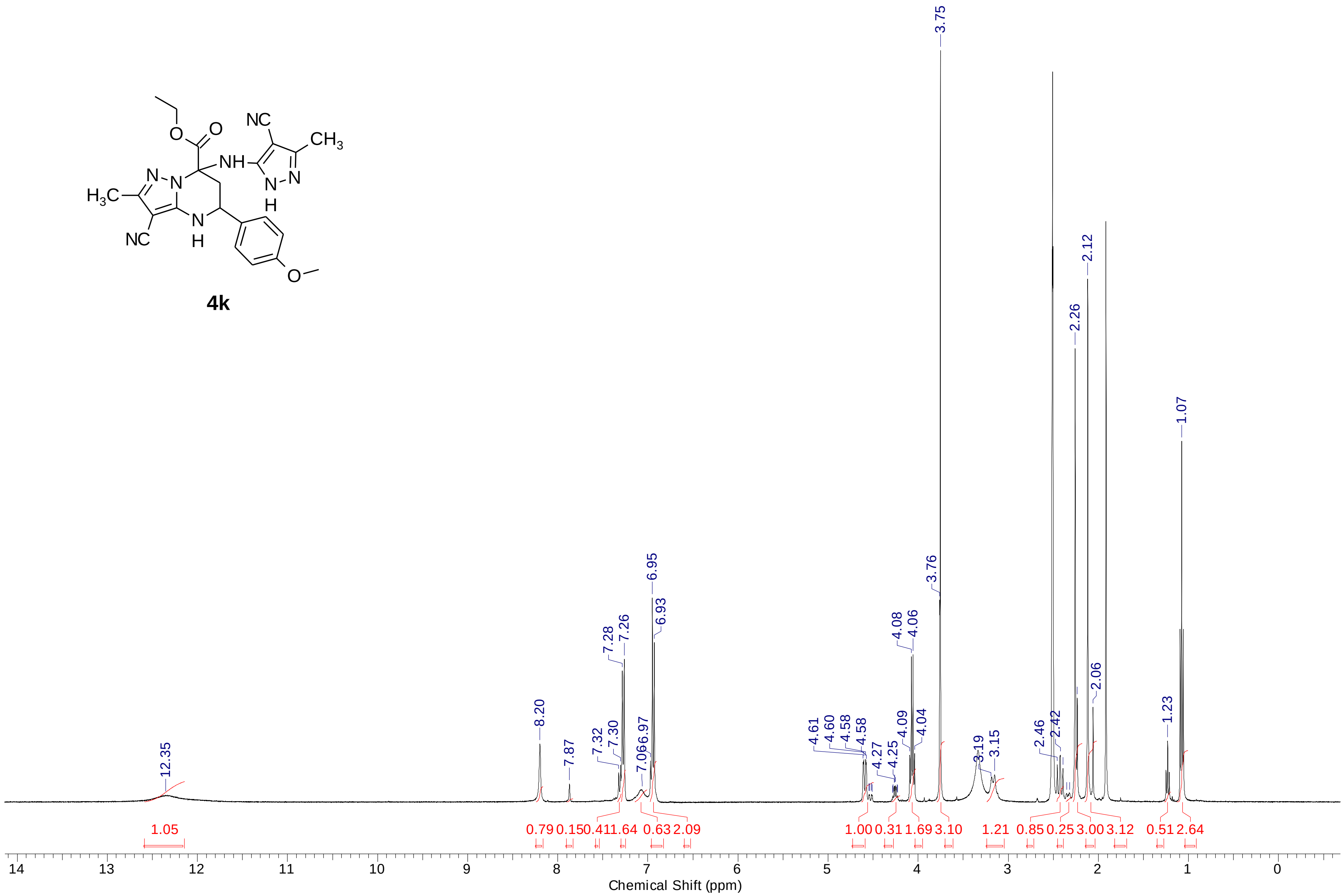

4k
NC
CH3
H3C
O
NH
N
H
Chemical Shift (ppm)

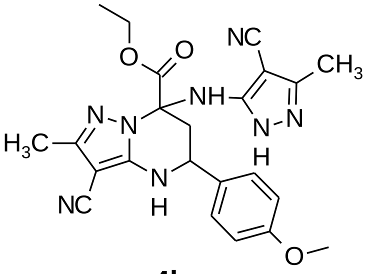
NC
CH_3
O
O
NH
N
N
H
H_3C
N
N
NC
N
H
O

4k

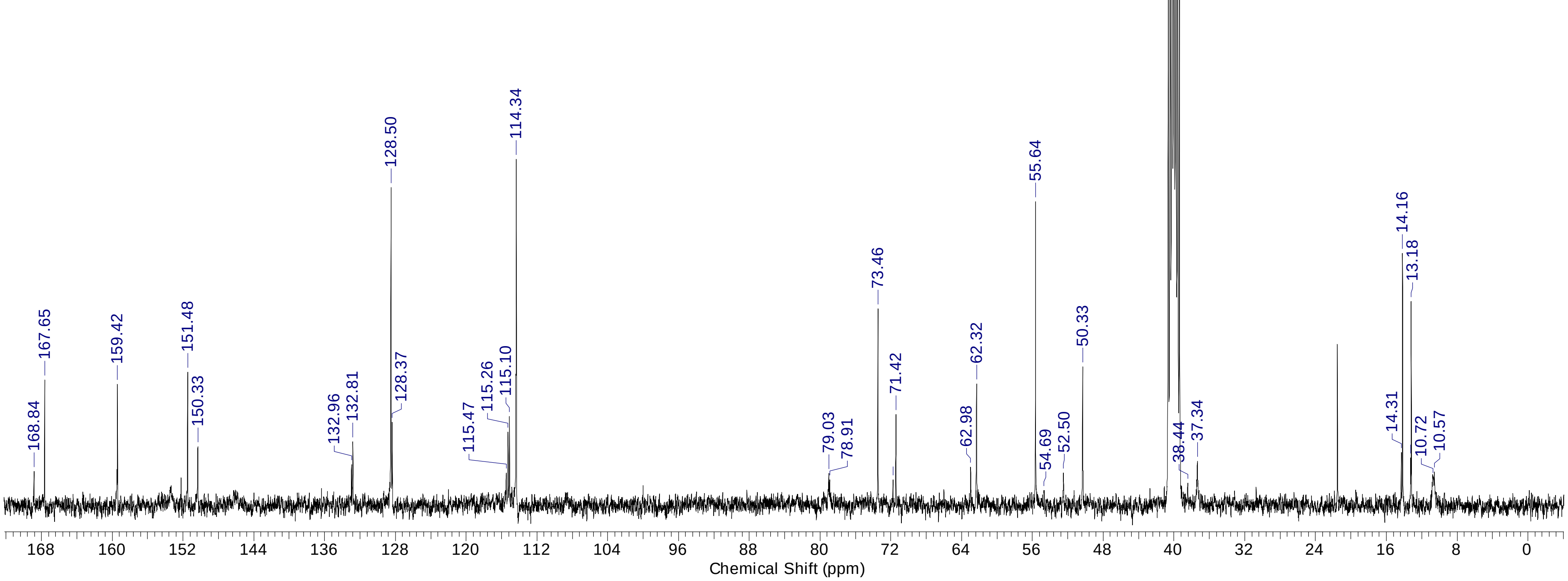
168.84
167.65
159.42
151.48
150.33
132.96
132.81
128.50
128.37
115.47
115.26
115.10
114.34
79.03
78.91
73.46
71.42
62.98
62.32
55.64
54.69
52.50
50.33
38.44
37.34
14.31
14.16
13.18
10.72
10.57
168 160 152 144 136 128 120 112 104 96 88 80 72 64 56 48 40 32 24 16 8 0
Chemical Shift (ppm)

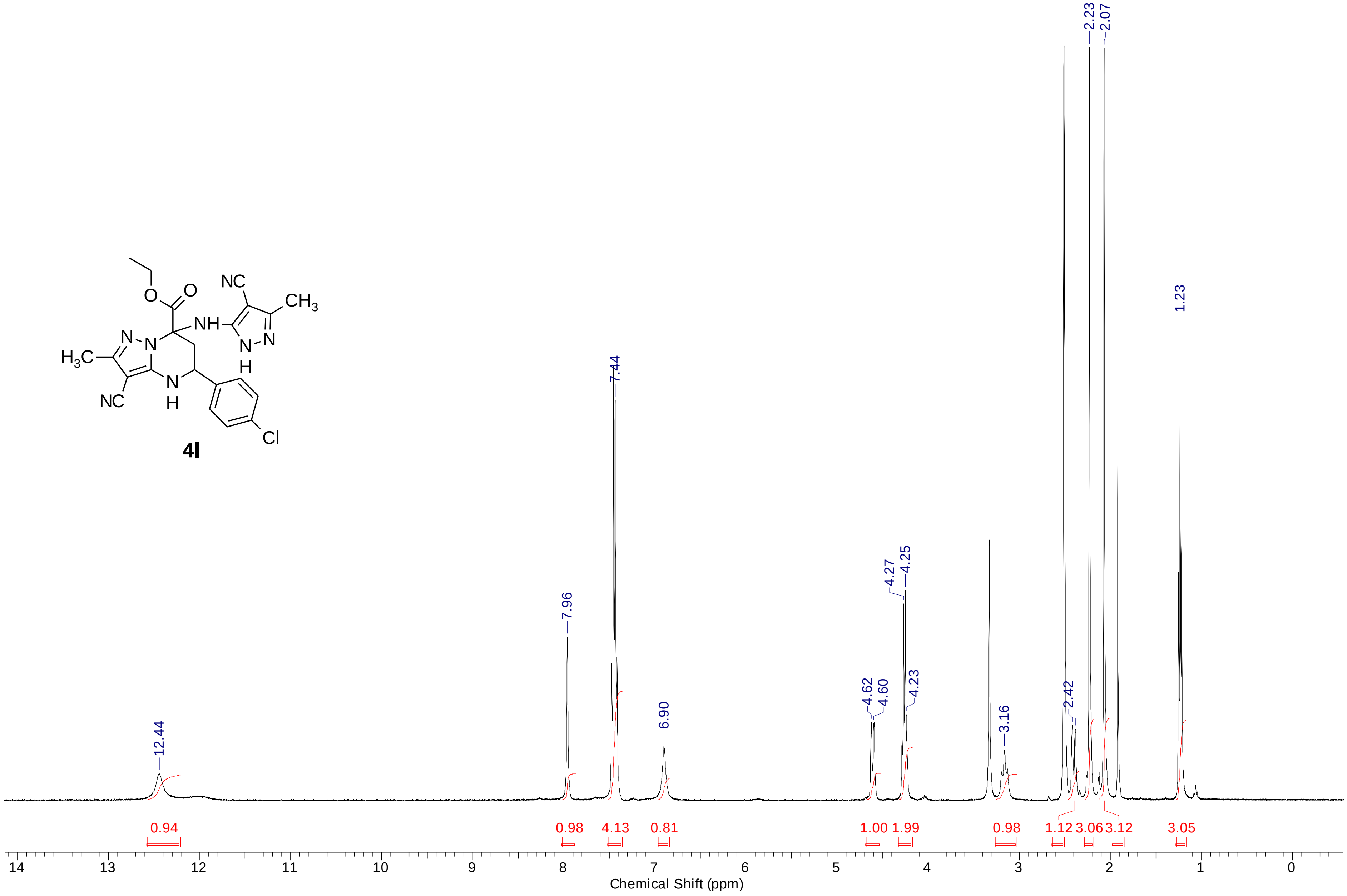
O
O
NC
CH3
NH
N
N
N
N
H
H3C
NC
N
H
Cl
4l
12.44
7.96
7.44
6.90
4.62
4.60
4.27
4.25
4.23
3.16
2.42
2.23
2.07
1.23
0.94
0.98
4.13
0.81
1.00
1.99
0.98
1.12
3.06
3.12
3.05
14
13
12
11
10
9
8
7
6
5
4
3
2
1
0
Chemical Shift (ppm)

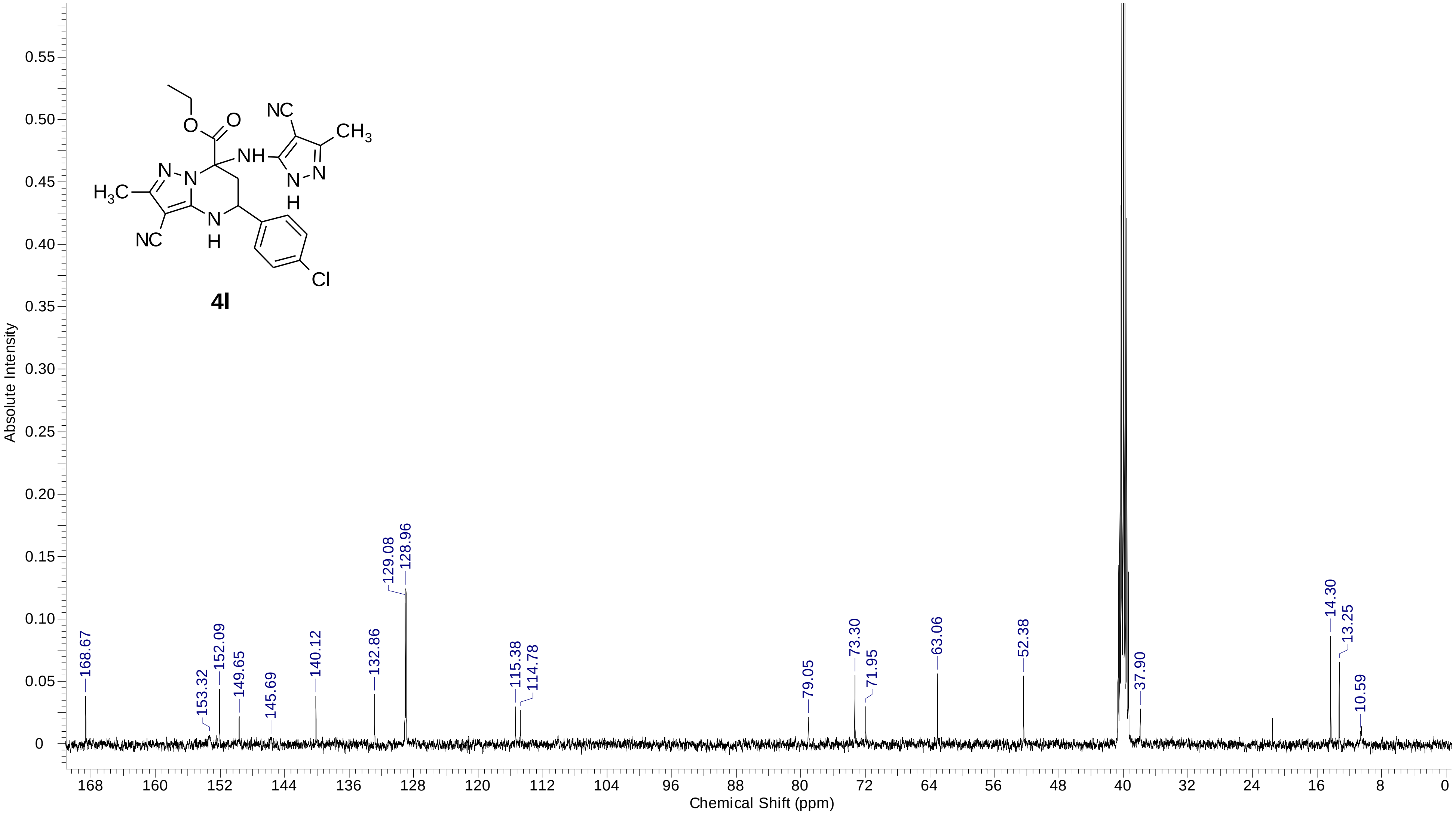
4l
NC
CH3
O
O
NH
N
N
N
N
H
H3C
NC
N
H
Cl
168.67
153.32
152.09
149.65
145.69
140.12
132.86
129.08
128.96
115.38
114.78
79.05
73.30
71.95
63.06
52.38
37.90
14.30
13.25
10.59
Absolute Intensity
Chemical Shift (ppm)
0.55
0.50
0.45
0.40
0.35
0.30
0.25
0.20
0.15
0.10
0.05
0
168
160
152
144
136
128
120
112
104
96
88
80
72
64
56
48
40
32
24
16
8
0

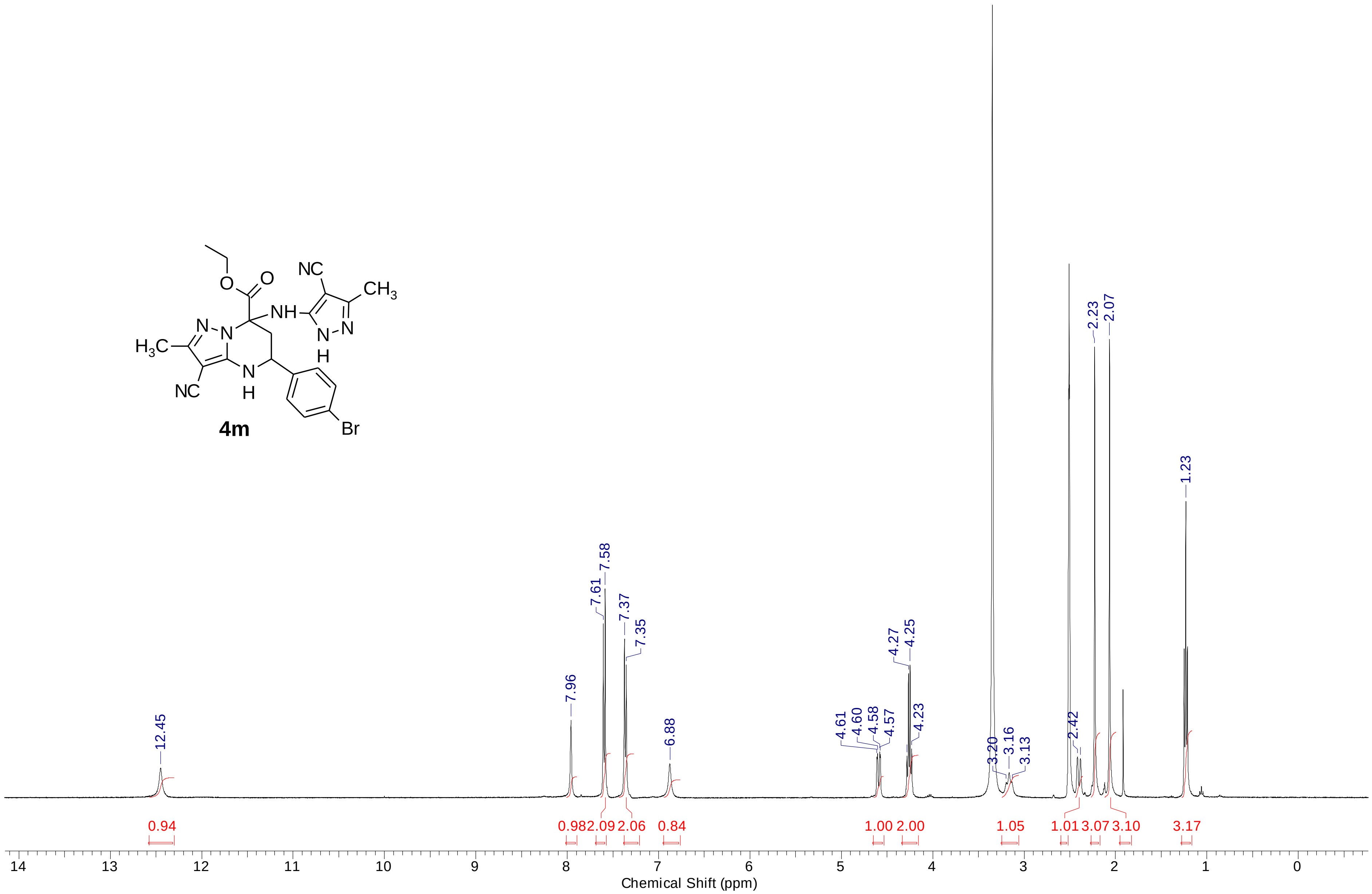
O
O
NC
CH3
NH
N
N
H3C
N
N
N
H
NC
N
H
Br
4m
12.45
7.96
7.61
7.58
7.37
7.35
6.88
4.61
4.60
4.58
4.57
4.27
4.25
4.23
3.20
3.16
3.13
2.42
2.23
2.07
1.23
0.94
0.98
2.09
2.06
0.84
1.00
2.00
1.05
1.01
3.07
3.10
3.17
14
13
12
11
10
9
8
7
6
5
4
3
2
1
0
Chemical Shift (ppm)

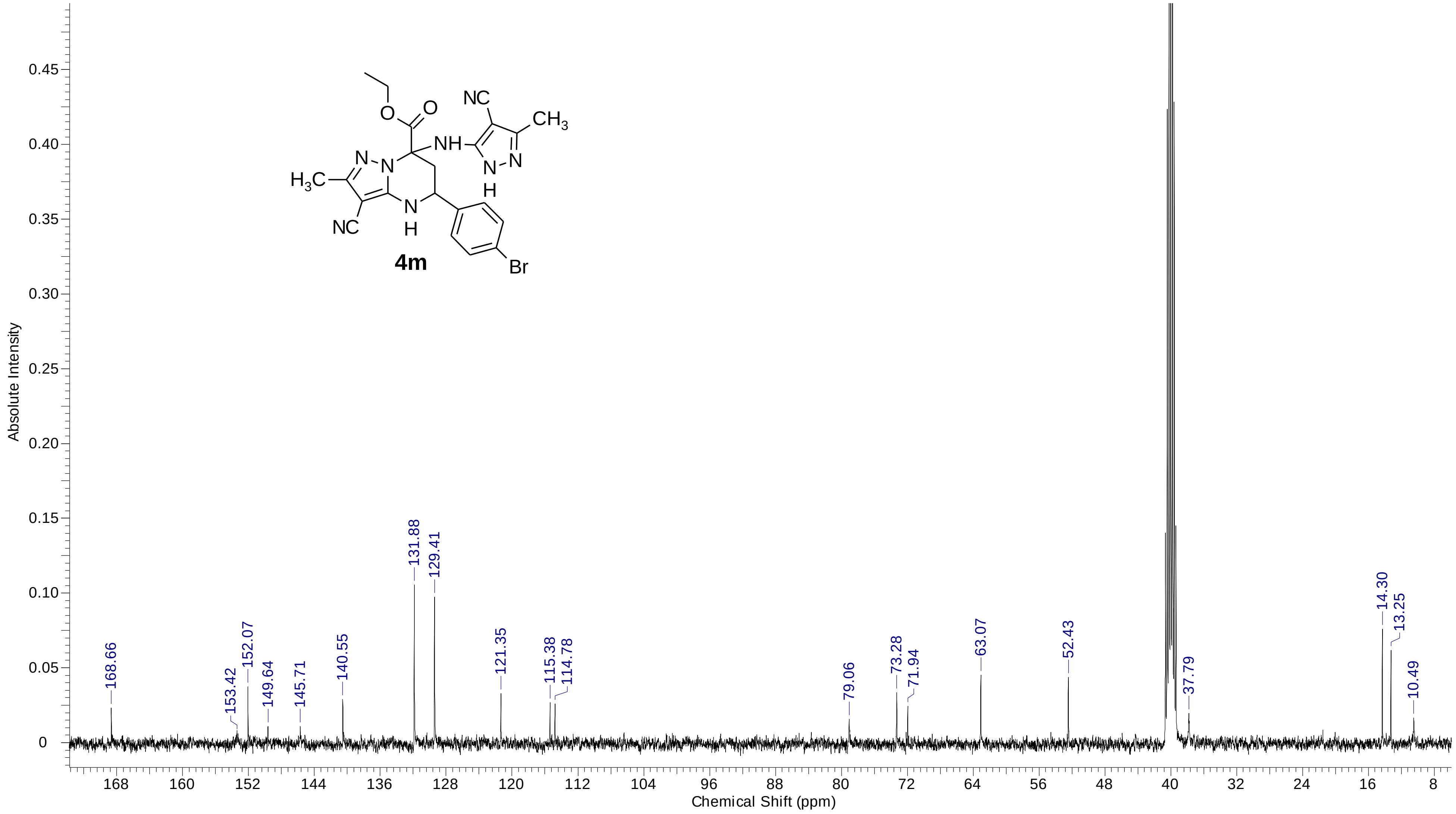
4m
NC
CH3
O
NH
N
H
H3C
NC
Br
168.66
153.42
152.07
149.64
145.71
140.55
131.88
129.41
121.35
115.38
114.78
79.06
73.28
71.94
63.07
52.43
37.79
14.30
13.25
10.49
Absolute Intensity
Chemical Shift (ppm)

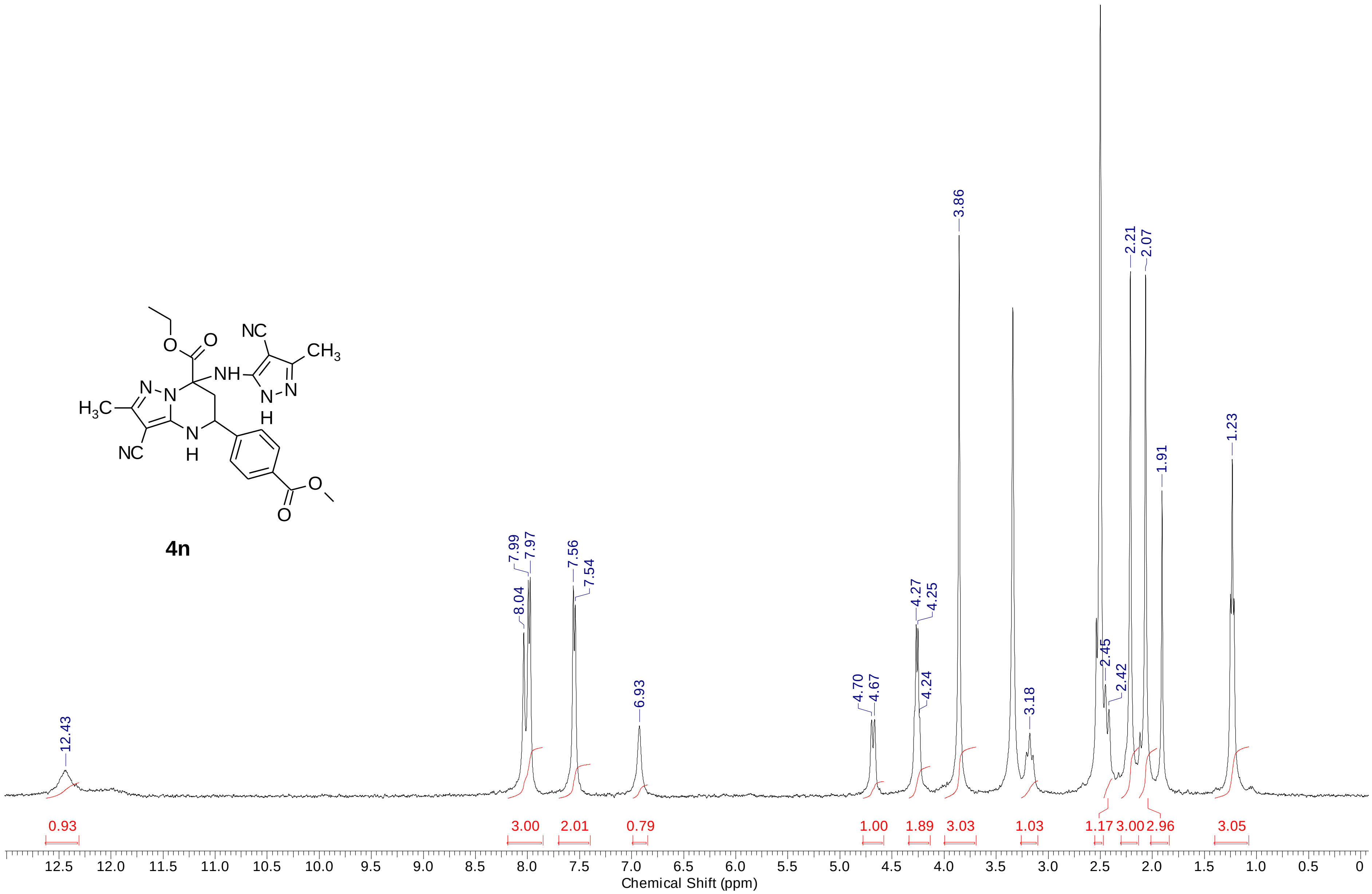

NC
CH3
O
O
NH
N
N
N
N
H3C
N
H
NC
H
O
O

4n

12.43
8.04
7.99
7.97
7.56
7.54
6.93
4.70
4.67
4.27
4.25
4.24
3.86
3.18
2.45
2.42
2.21
2.07
1.91
1.23
0.93
3.00
2.01
0.79
1.00
1.89
3.03
1.03
1.17
3.00
2.96
3.05
12.5 12.0 11.5 11.0 10.5 10.0 9.5 9.0 8.5 8.0 7.5 7.0 6.5 6.0 5.5 5.0 4.5 4.0 3.5 3.0 2.5 2.0 1.5 1.0 0.5 0
Chemical Shift (ppm)

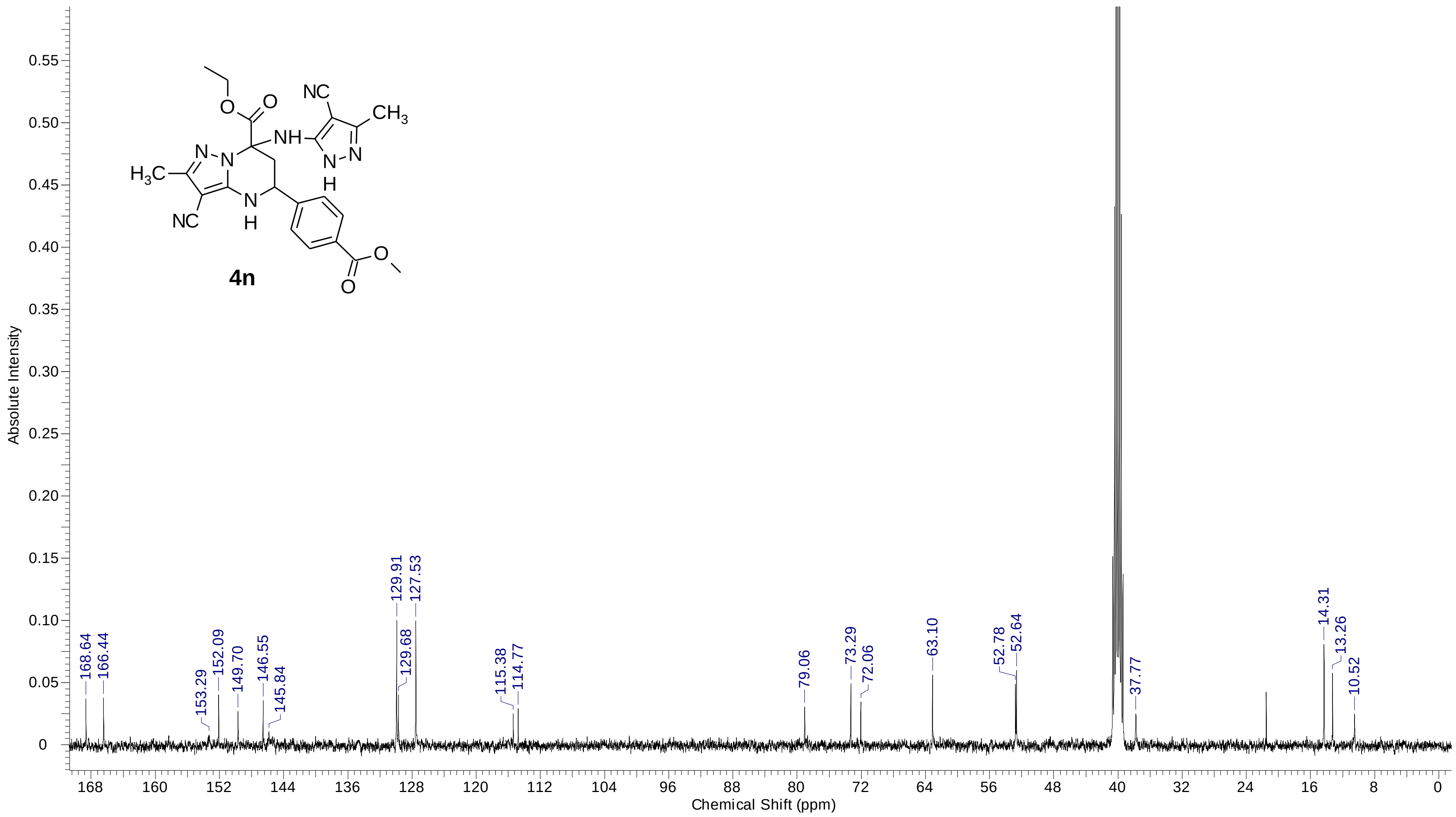
NC
CH3
O
O
NH
N
N
N
N
H3C
N
H
NC
N
H
O
O
4n
Absolute Intensity
0.55
0.50
0.45
0.40
0.35
0.30
0.25
0.20
0.15
0.10
0.05
0
168.64
166.44
153.29
152.09
149.70
146.55
145.84
129.91
129.68
127.53
115.38
114.77
79.06
73.29
72.06
63.10
52.78
52.64
37.77
14.31
13.26
10.52
168
160
152
144
136
128
120
112
104
96
88
80
72
64
56
48
40
32
24
16
8
0
Chemical Shift (ppm)

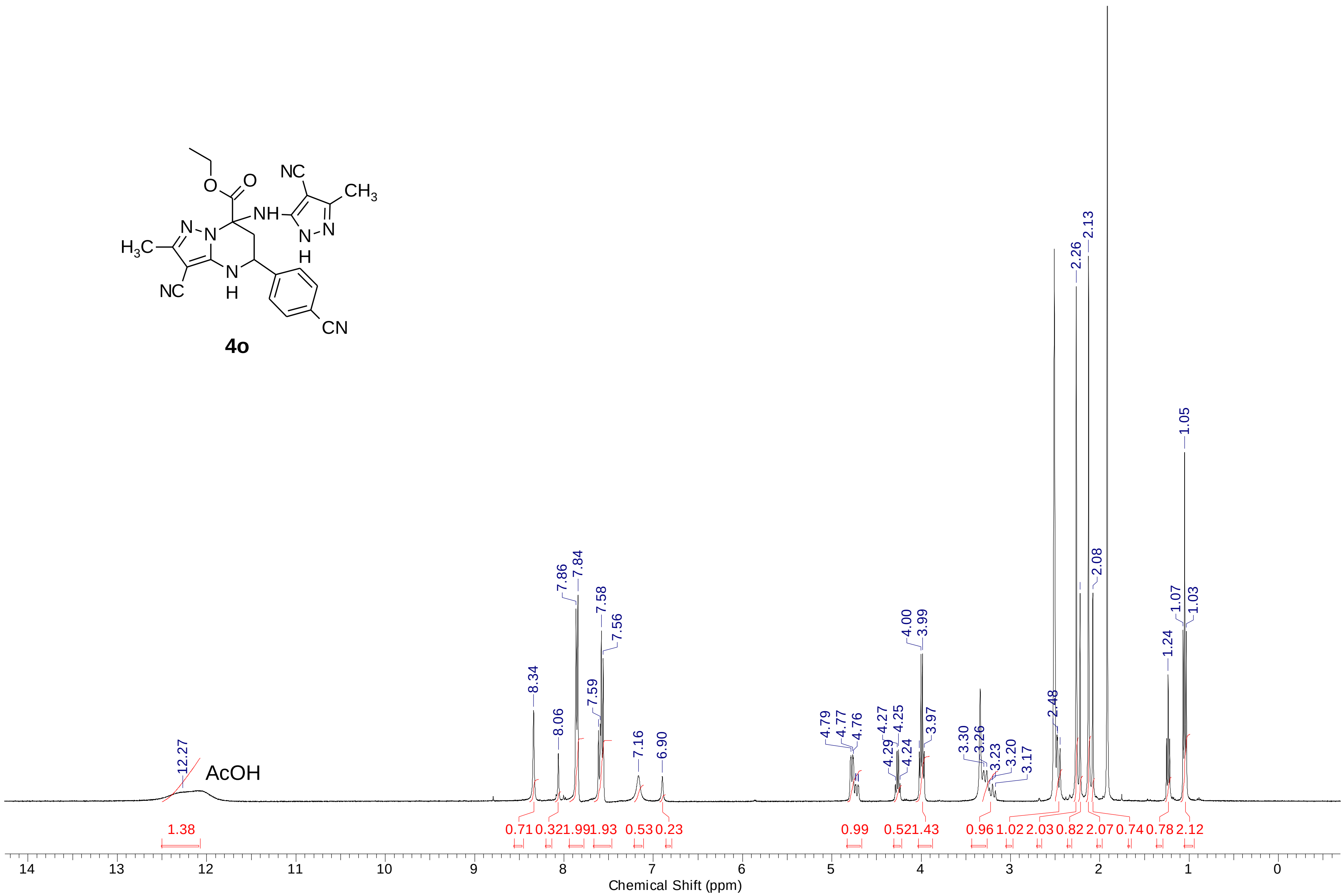
4o
AcOH
12.27
8.34
8.06
7.86
7.84
7.59
7.58
7.56
7.16
6.90
4.79
4.77
4.76
4.29
4.27
4.25
4.24
4.00
3.99
3.97
3.30
3.26
3.23
3.20
3.17
2.48
2.26
2.13
2.08
1.24
1.07
1.05
1.03
1.38
0.71
0.32
1.99
1.93
0.53
0.23
0.99
0.52
1.43
0.96
1.02
2.03
0.82
2.07
0.74
0.78
2.12
Chemical Shift (ppm)

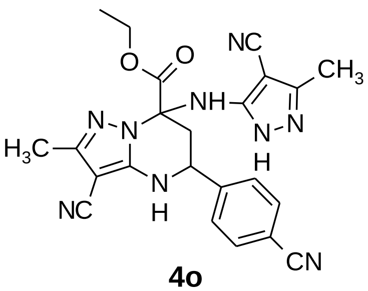
NC
CH3
O
O
NH
N
N
N
H3C
N
H
N
H
NC
CN

4o

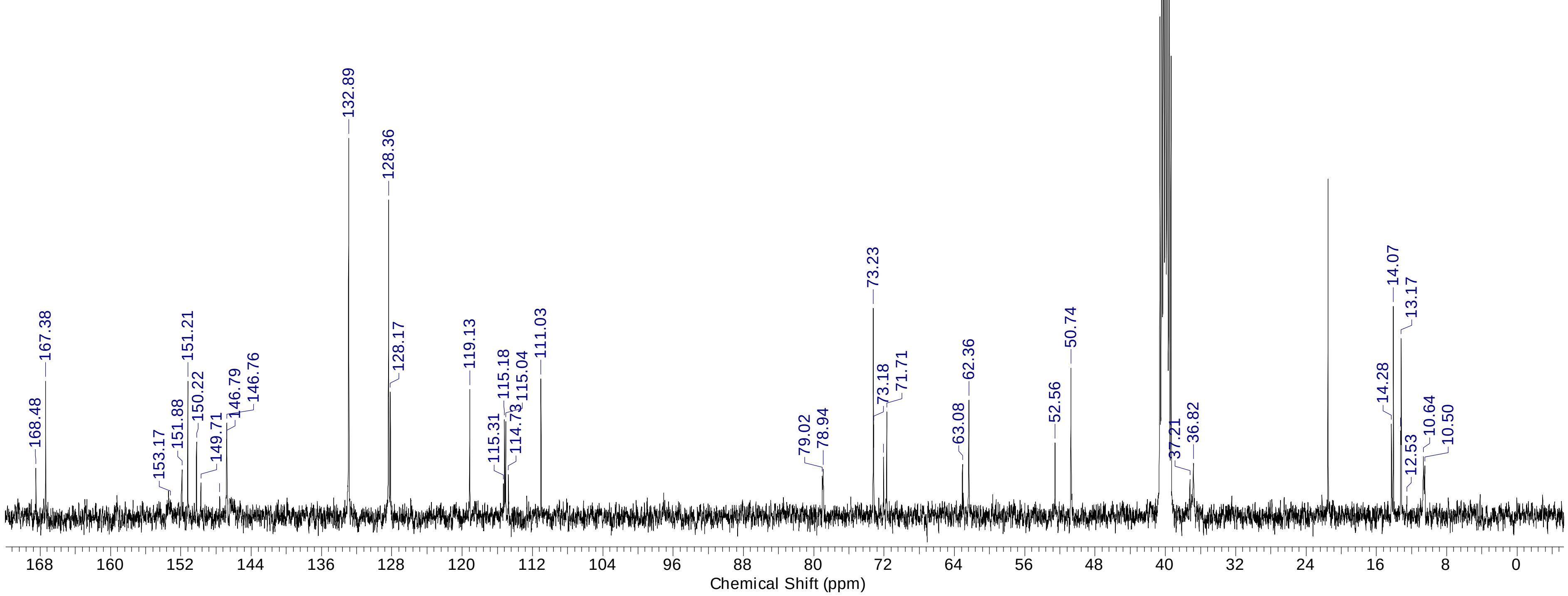
168.48
167.38
153.17
151.88
151.21
150.22
149.71
146.79
146.76
132.89
128.36
128.17
119.13
115.31
115.18
114.73
115.04
111.03
79.02
78.94
73.23
73.18
71.71
63.08
62.36
52.56
50.74
37.21
36.82
14.28
14.07
13.17
12.53
10.64
10.50
168 160 152 144 136 128 120 112 104 96 88 80 72 64 56 48 40 32 24 16 8 0
Chemical Shift (ppm)

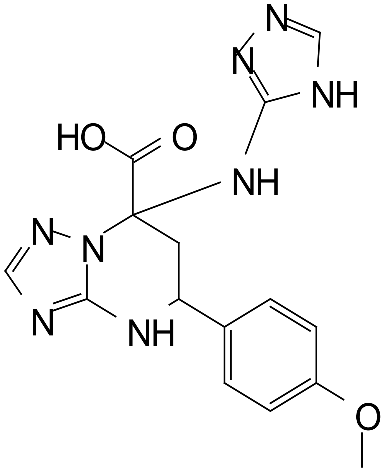
N
N
NH
HO
O
NH
N
N
N
NH
O

4p

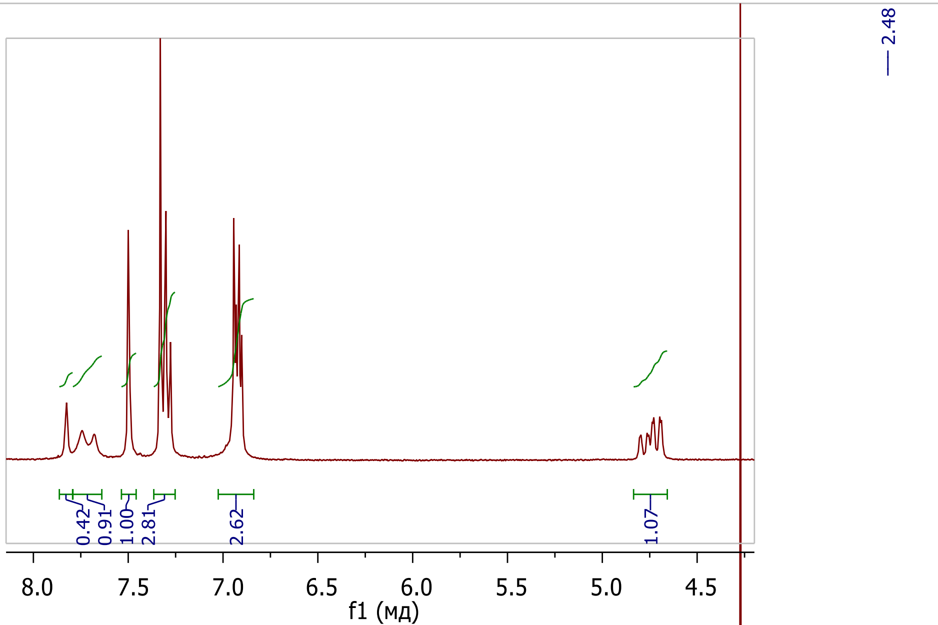
2.48
0.42
0.91
1.00
2.81
2.62
1.07
8.0
7.5
7.0
6.5
6.0
5.5
5.0
4.5
f1 (мд)

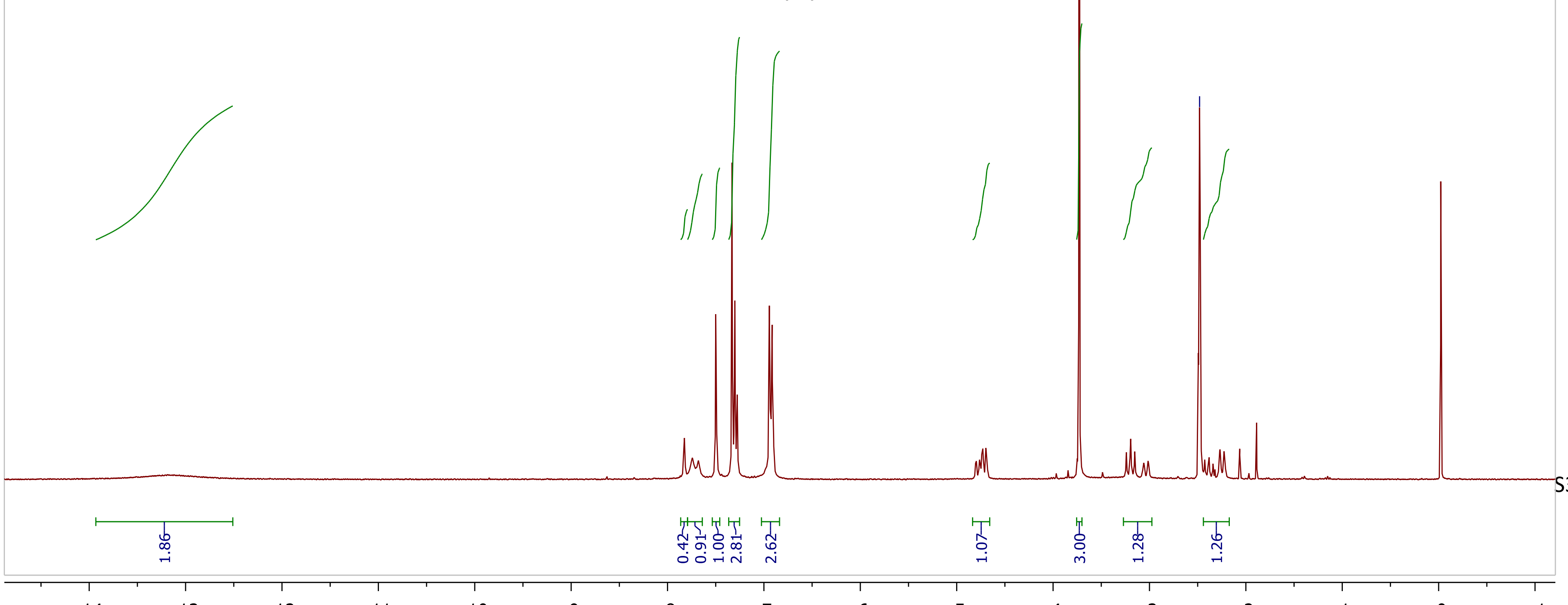
1.86
0.42
0.91
1.00
2.81
2.62
1.07
3.00
1.28
1.26

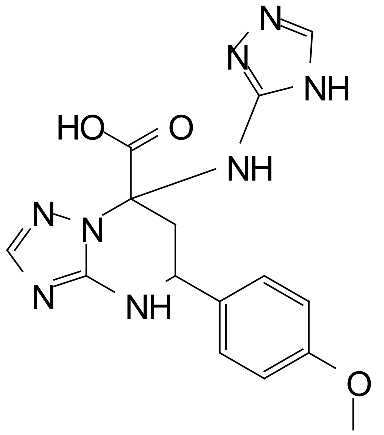

4p

^{13}C NMR (75 MHz, DMSO δ) 170.21, 169.26, 158.82, 155.30, 154.87, 133.16, 132.81, 127.73, 113.89, 72.03, 55.09, 52.37, 50.71.

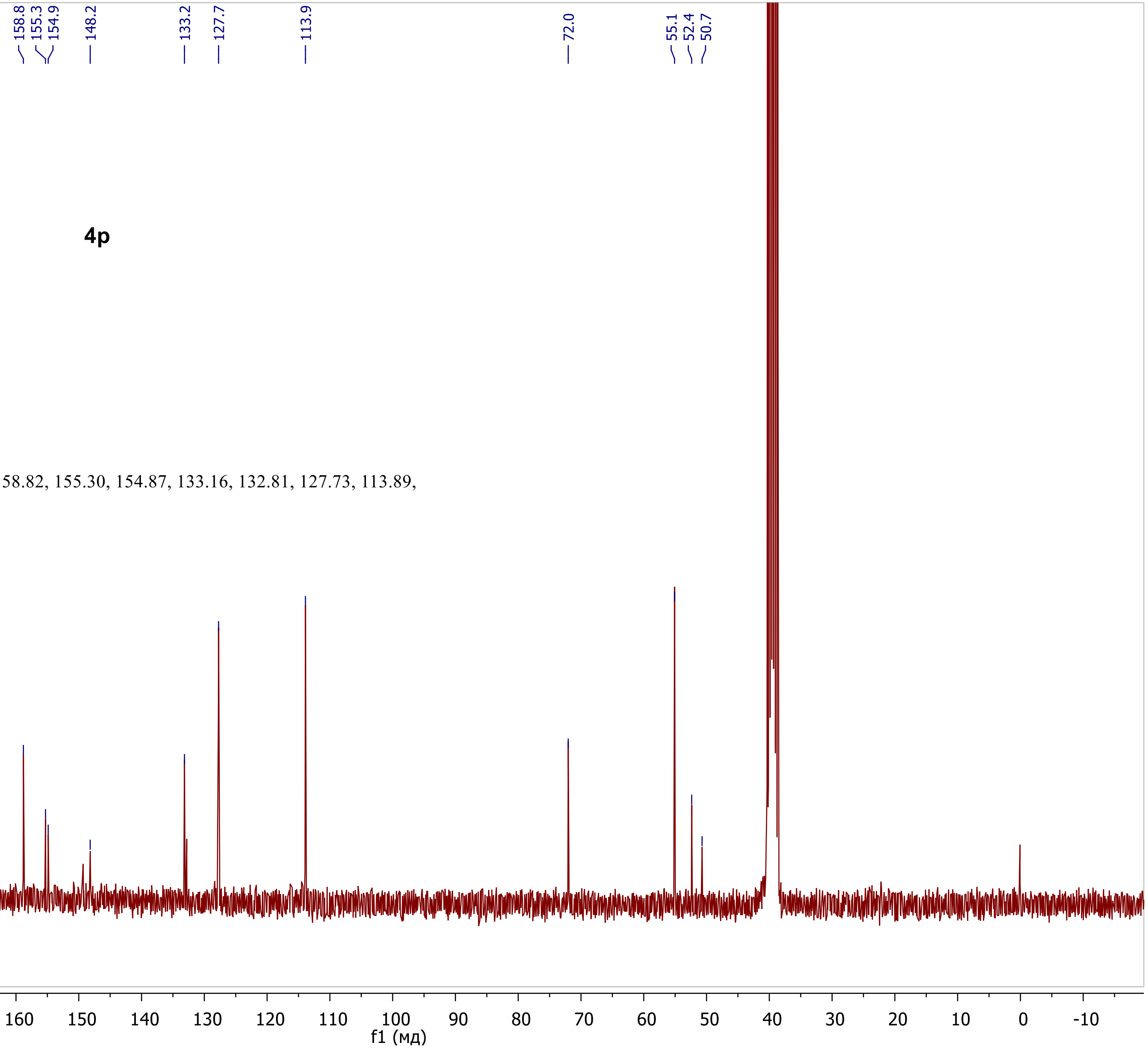

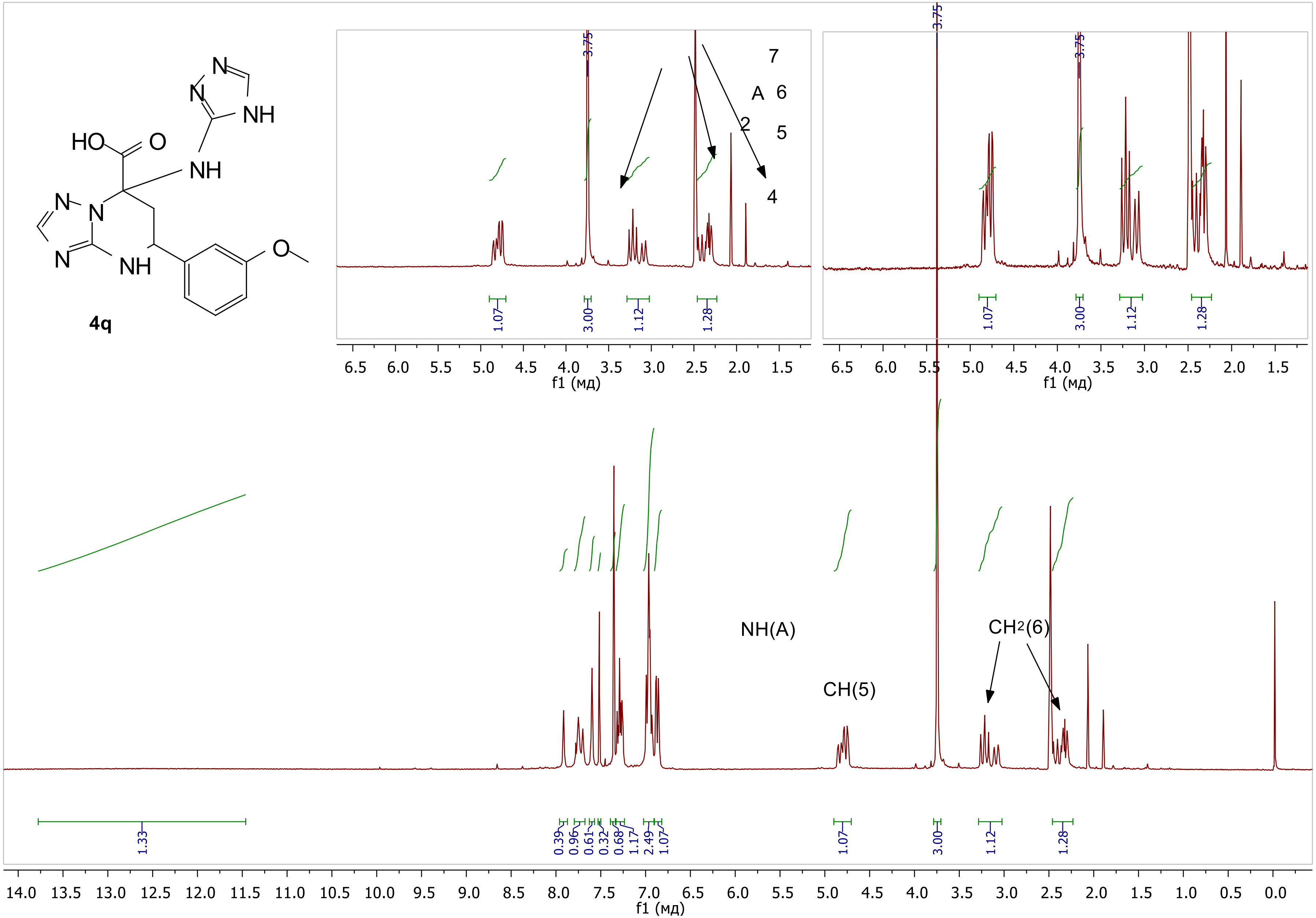
4q
NH(A)
CH(5)
CH2(6)
7
A 6
2
5
4
f1 (мд)

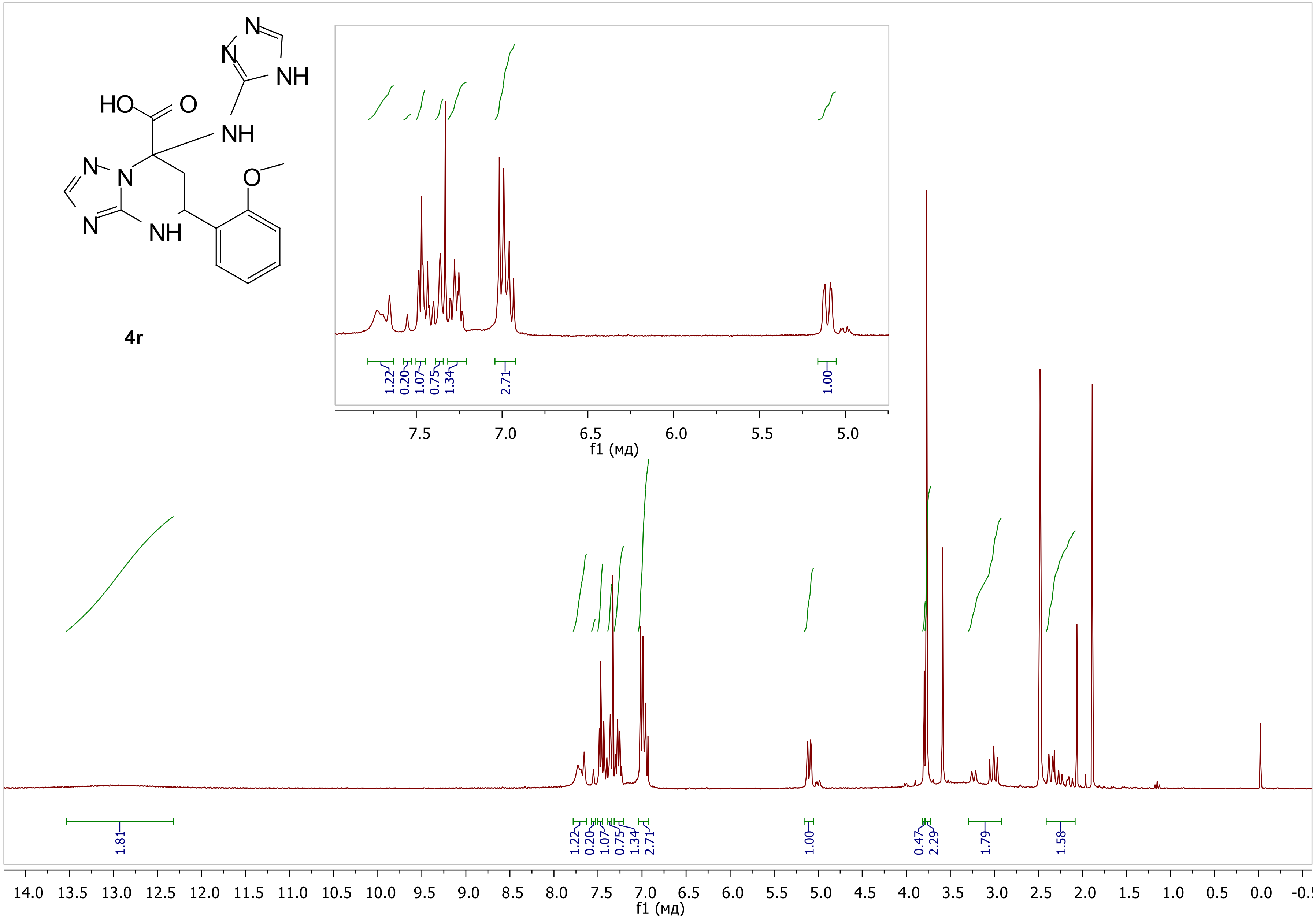

HO
O
NH
N
NH
N
N
N
NH
O
4r
1.22
0.20
1.07
0.75
1.34
2.71
1.00
7.5
7.0
6.5
6.0
5.5
5.0
f1 (мд)
1.81
0.47
2.29
1.79
1.58
14.0 13.5 13.0 12.5 12.0 11.5 11.0 10.5 10.0 9.5 9.0 8.5 8.0 7.5 7.0 6.5 6.0 5.5 5.0 4.5 4.0 3.5 3.0 2.5 2.0 1.5 1.0 0.5 0.0
f1 (мд)

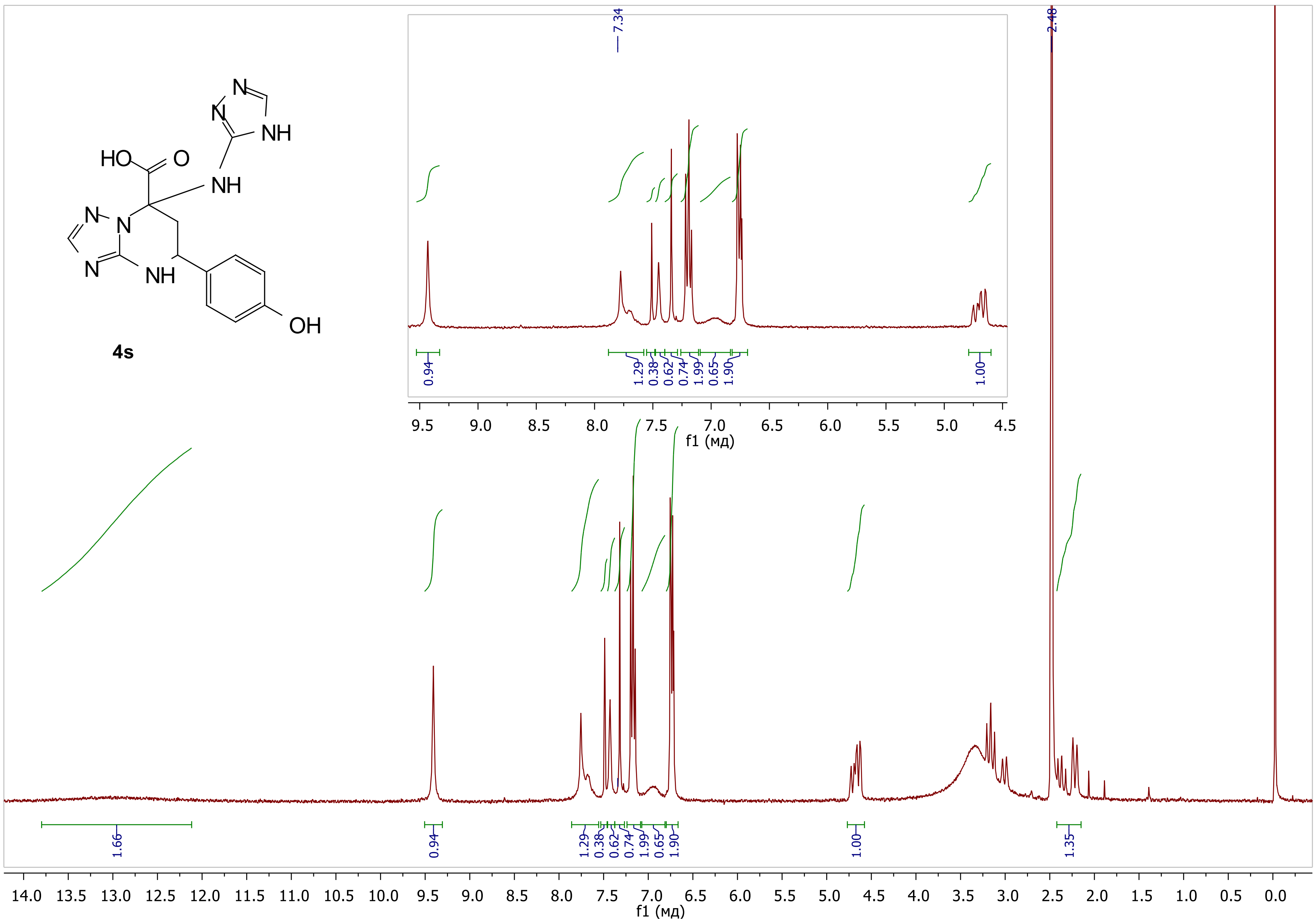

4s
7.34
2.48
0.94
1.29
0.38
0.62
0.74
1.99
0.65
1.90
1.00
1.66
1.35
f1 (мд)

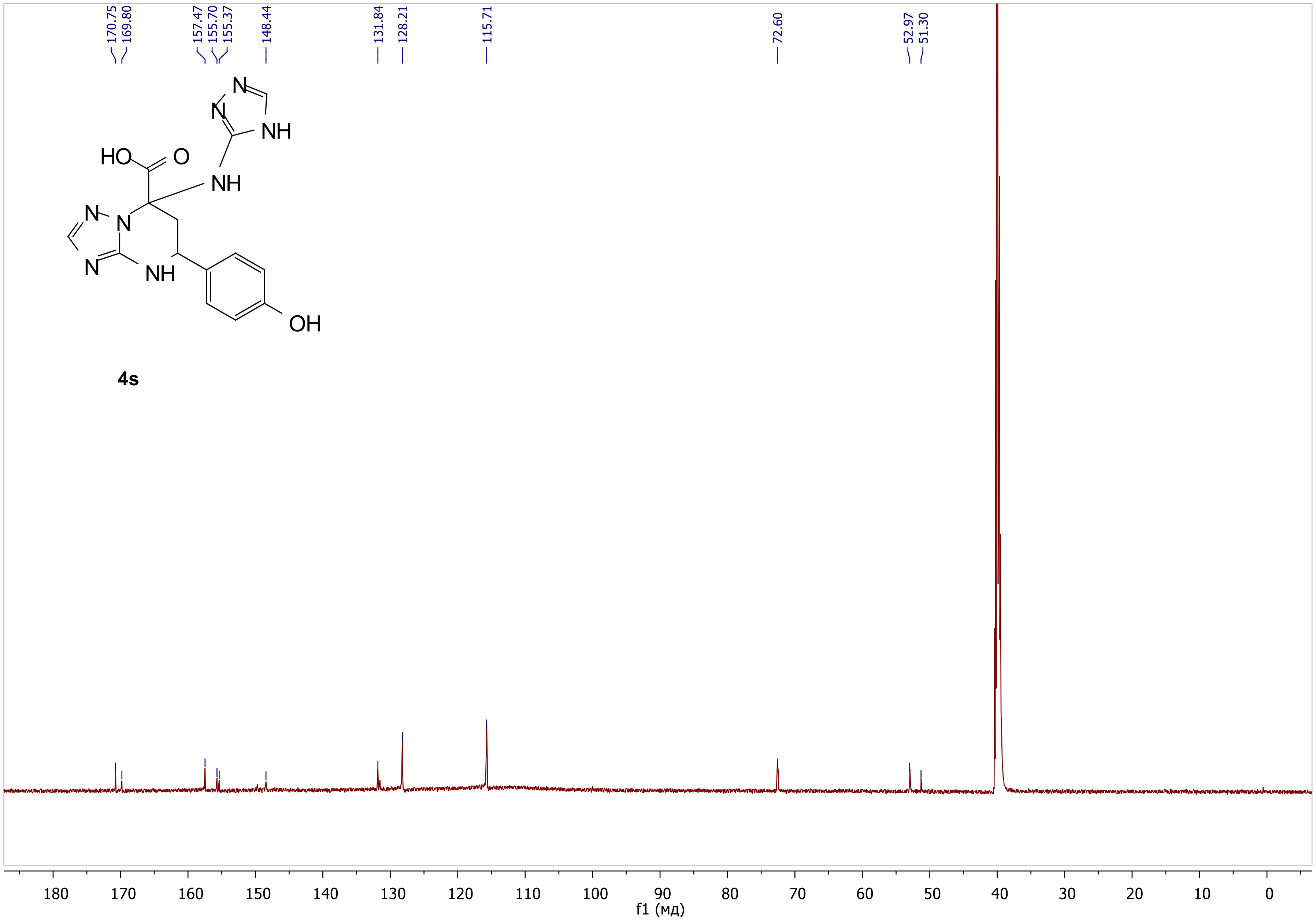
170.75
169.80
157.47
155.70
155.37
148.44
131.84
128.21
115.71
72.60
52.97
51.30
HO
O
NH
N
N
NH
N
N
N
NH
OH
4s
180 170 160 150 140 130 120 110 100 90 80 70 60 50 40 30 20 10 0
f1 (мд)

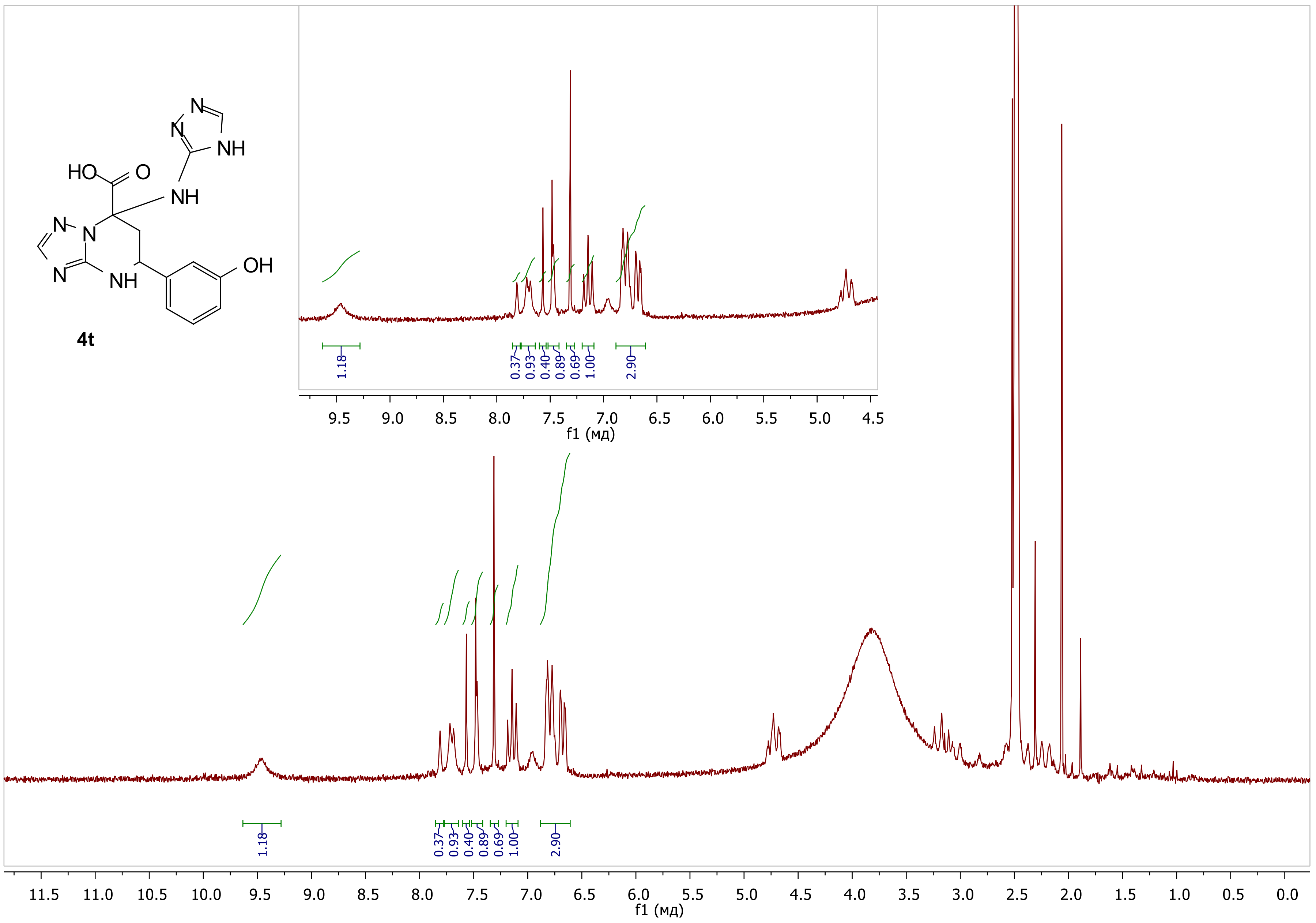
HO
O
N
N
NH
NH
N
N
N
N
NH
OH
4t
1.18
0.37
0.93
0.40
0.89
0.69
1.00
2.90
9.5 9.0 8.5 8.0 7.5 7.0 6.5 6.0 5.5 5.0 4.5
f1 (мд)
11.5 11.0 10.5 10.0 9.5 9.0 8.5 8.0 7.5 7.0 6.5 6.0 5.5 5.0 4.5 4.0 3.5 3.0 2.5 2.0 1.5 1.0 0.5 0.0
f1 (мд)

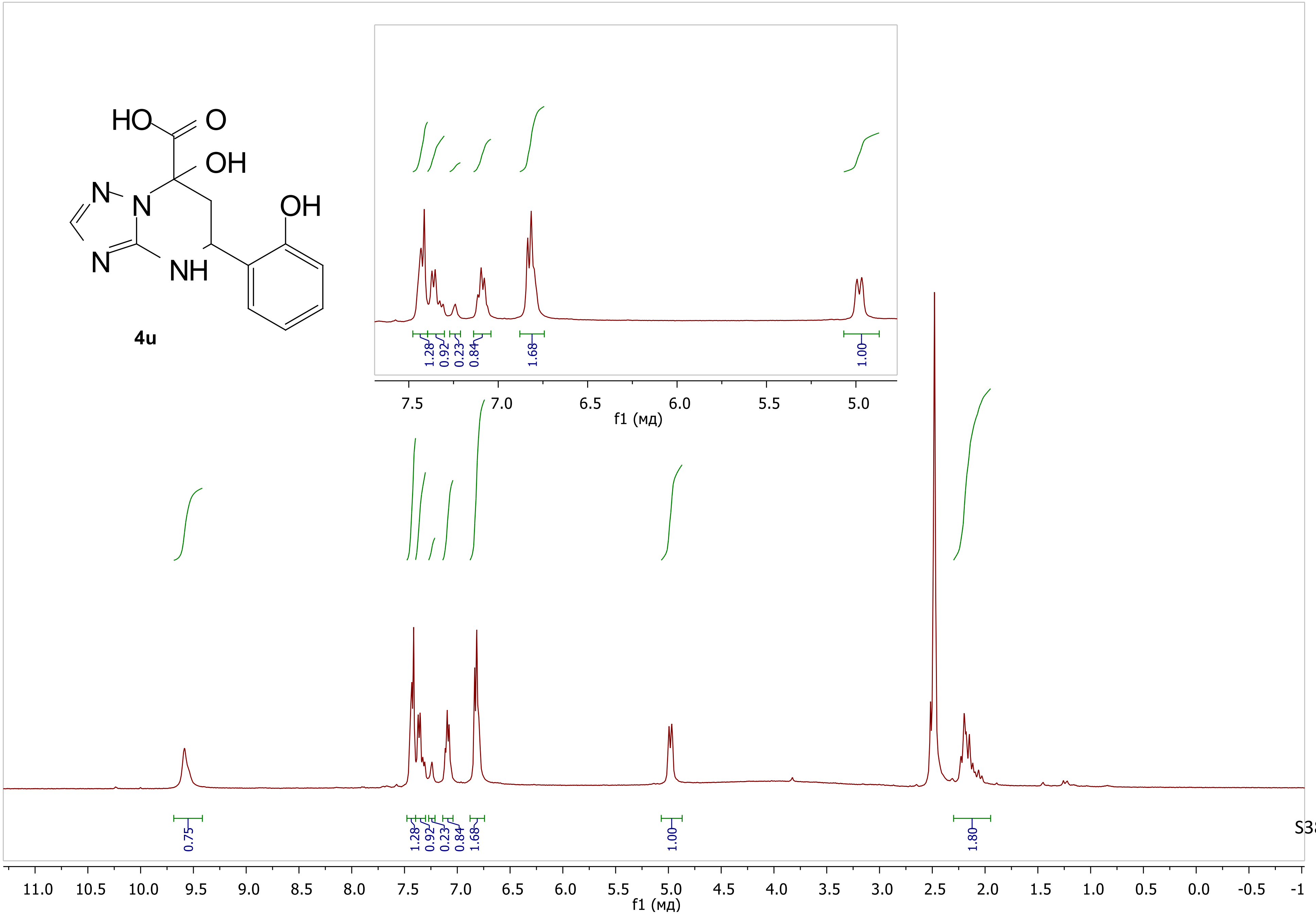
4u
1.28
0.92
0.23
0.84
1.68
1.00
7.5
7.0
6.5
6.0
5.5
5.0
f1 (мд)
0.75
1.28
0.92
0.23
0.84
1.68
1.00
1.80
11.0 10.5 10.0 9.5 9.0 8.5 8.0 7.5 7.0 6.5 6.0 5.5 5.0 4.5 4.0 3.5 3.0 2.5 2.0 1.5 1.0 0.5 0.0 -0.5 -1
f1 (мд)

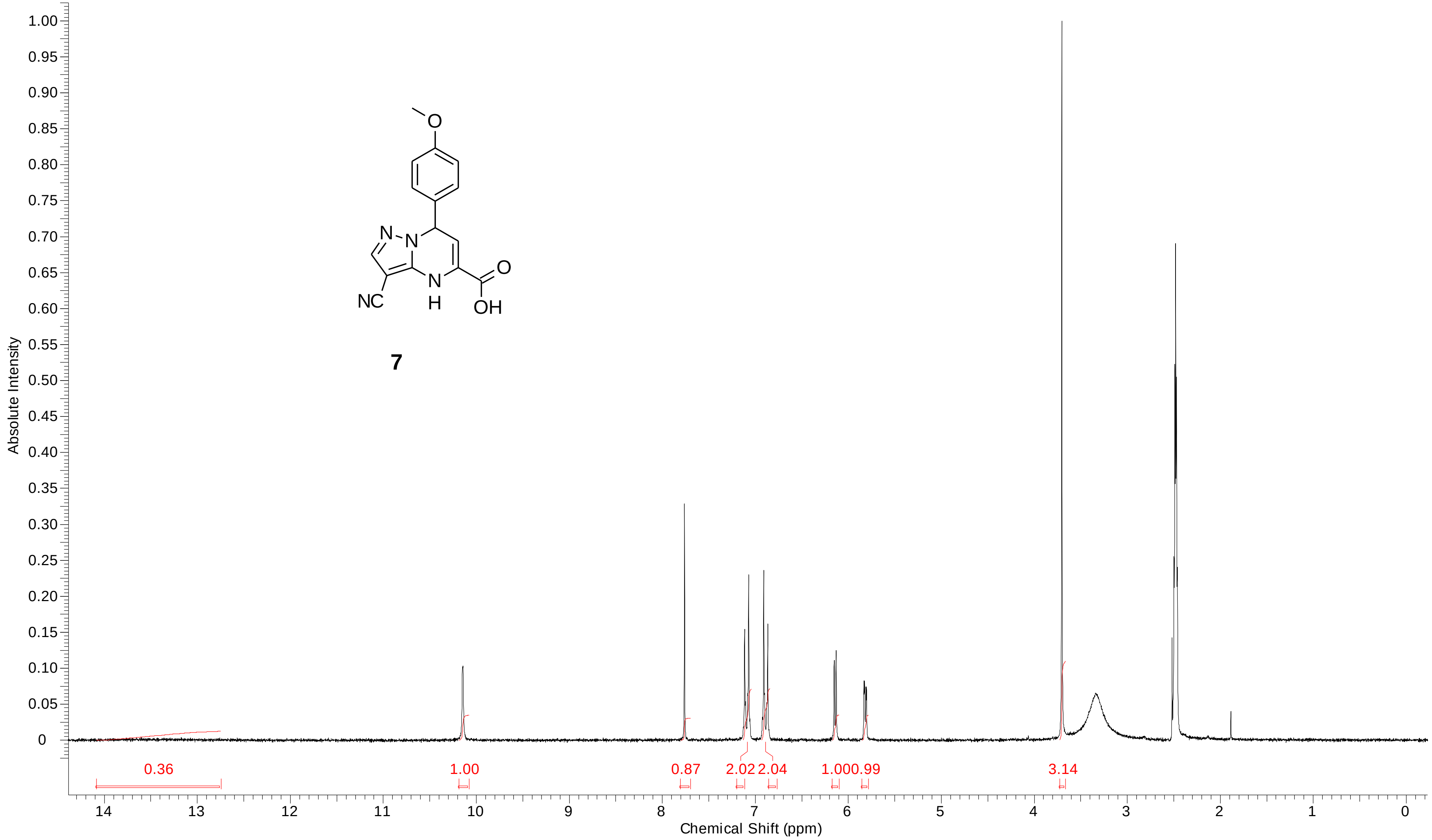
O
N
N
N
H
NC
O
OH
7
Absolute Intensity
1.00
0.95
0.90
0.85
0.80
0.75
0.70
0.65
0.60
0.55
0.50
0.45
0.40
0.35
0.30
0.25
0.20
0.15
0.10
0.05
0
0.36
1.00
0.87
2.02
2.04
1.00
0.99
3.14
14
13
12
11
10
9
8
7
6
5
4
3
2
1
0
Chemical Shift (ppm)

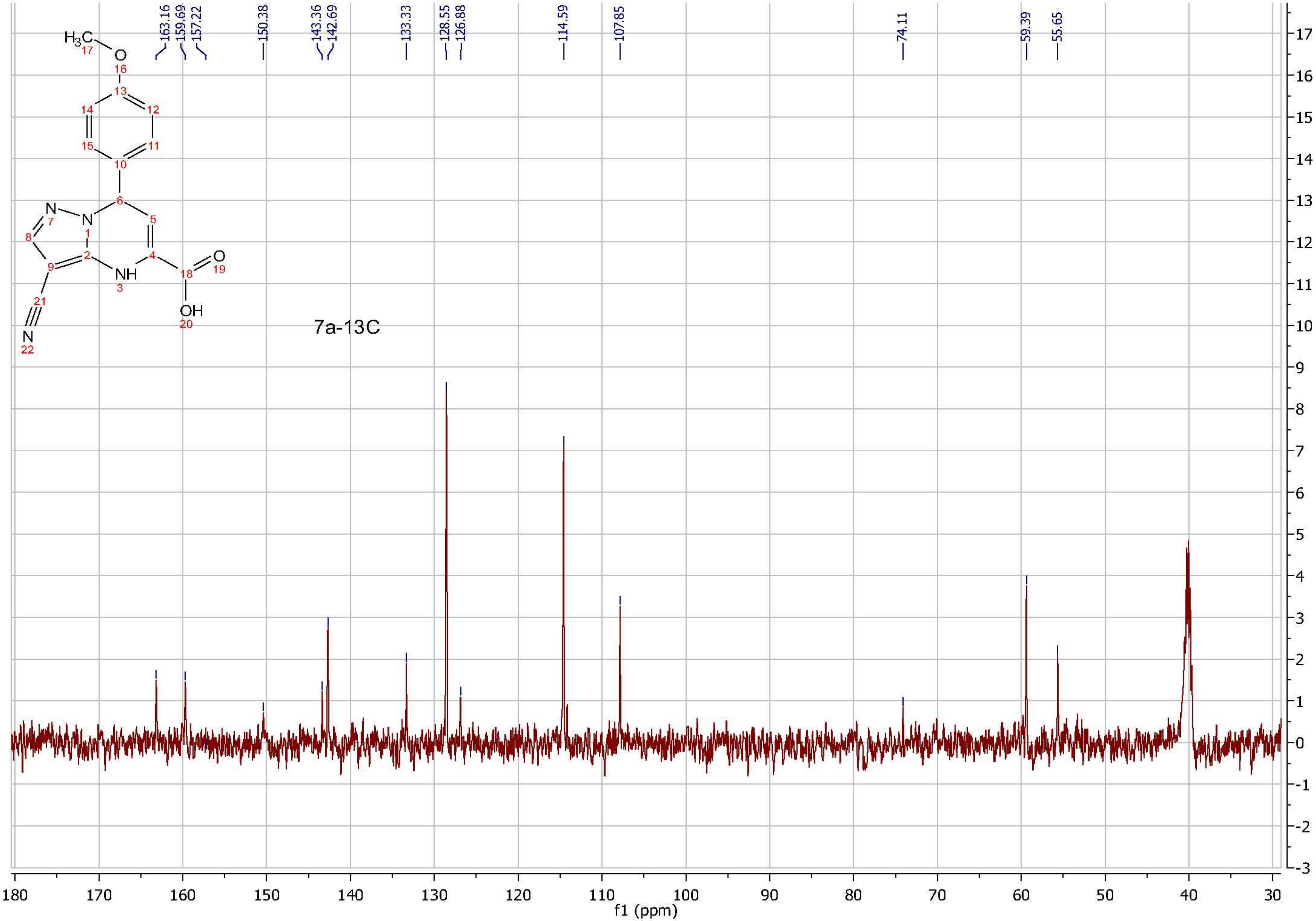

7a-13C
163.16
159.69
157.22
150.38
143.36
142.69
133.33
128.55
126.88
114.59
107.85
74.11
59.39
55.65
f1 (ppm)
